# Localized Hypermutation is the Major Driver of Meningococcal Genetic Variability during Persistent Asymptomatic Carriage

**DOI:** 10.1128/mBio.03068-19

**Published:** 2020-03-24

**Authors:** Luke R. Green, Ali A. Al-Rubaiawi, Mohammad A. R. M. Al-Maeni, Odile B. Harrison, Matthew Blades, Neil J. Oldfield, David P. J. Turner, Martin C. J. Maiden, Christopher D. Bayliss

**Affiliations:** a Department of Genetics and Genome Biology, University of Leicester, Leicester, United Kingdom; b Department of Zoology, University of Oxford, Oxford, United Kingdom; c BBASH, University of Leicester, Leicester, United Kingdom; d School of Life Sciences, University of Nottingham, Nottingham, United Kingdom; GSK Vaccines; GSK Vaccines

**Keywords:** *Neisseria meningitidis*, horizontal gene transfer, localized hypermutation, meningitis, meningococcus, phase variation, whole-genome sequence

## Abstract

Host persistence of bacteria is facilitated by mutational and recombinatorial processes that counteract loss of genetic variation during transmission and selection from evolving host responses. Genetic variation was investigated during persistent asymptomatic carriage of Neisseria meningitidis. Interrogation of whole-genome sequences for paired isolates from 25 carriers showed that *de novo* mutations were infrequent, while horizontal gene transfer occurred in 16% of carriers. Examination of multiple isolates per time point enabled separation of sporadic and transient allelic variation from directional variation. A comprehensive comparative analysis of directional allelic variation with hypermutation of simple sequence repeats and hyperrecombination of class 1 type IV pilus genes detected an average of seven events per carrier and 2:1 bias for changes due to localized hypermutation. Directional genetic variation was focused on the outer membrane with 69% of events occurring in genes encoding enzymatic modifiers of surface structures or outer membrane proteins. Multiple carriers exhibited directional and opposed switching of allelic variants of the surface-located Opa proteins that enables continuous expression of these adhesins alongside antigenic variation. A trend for switching from PilC1 to PilC2 expression was detected, indicating selection for specific alterations in the activities of the type IV pilus, whereas phase variation of restriction modification (RM) systems, as well as associated phasevarions, was infrequent. We conclude that asymptomatic meningococcal carriage on mucosal surfaces is facilitated by frequent localized hypermutation and horizontal gene transfer affecting genes encoding surface modifiers such that optimization of adhesive functions occurs alongside escape of immune responses by antigenic variation.

## Abstract (precis)

Many bacterial pathogens coexist with host organisms, rarely causing disease while adapting to host responses. Neisseria meningitidis, a major cause of meningitis and septicemia, is a frequent persistent colonizer of asymptomatic teenagers/young adults. To assess how genetic variation contributes to host persistence, whole-genome sequencing and hypermutable sequence analyses were performed on multiple isolates obtained from students naturally colonized with meningococci. High frequencies of gene transfer were observed, occurring in 16% of carriers and affecting 51% of all nonhypermutable variable genes. Comparative analyses showed that hypermutable sequences were the major mechanism of variation, causing 2-fold more changes in gene function than other mechanisms. Genetic variation was focused on genes affecting the outer membrane, with directional changes in proteins responsible for bacterial adhesion to host surfaces. This comprehensive examination of genetic plasticity in individual hosts provides a significant new platform for rationale design of approaches to prevent the spread of this pathogen.

## INTRODUCTION

Persistence of colonizing organisms for extended periods in individual hosts perpetuates transmission and contributes to the spread of a diverse spectrum of protozoan, prokaryotic, and viral pathogens and commensals (1–3). Persistence is antagonized by innate and adaptive immune responses of the host, causing frequent reductions in population size of the persisting population, and can lead to evasion of immune responses by rapid evolution of adaptive variants (4). Reductions in population size due to nonselective bottlenecks or selection of fitness variants depletes the persisting population of genetic variation and reduces the capacity to respond to further selective pressures. This restricted genetic diversity is counteracted by *de novo* mutation, horizontal gene transfer (HGT), and localized hypermutation (LH), a descriptor of evolved mechanisms for rapid generation of genetic variation such as site-specific recombination, homologous recombination between multicopy allelic sequences, and simple sequence repeats (5, 6). Understanding the relative contributions of these processes to “within-host” evolution is important for discerning the key host-pathogen interactions that facilitate host persistence of bacterial pathogens and immune evasion.

Multiple repetitive sequences and high levels of genetic variability are notable attributes of Neisseria meningitidis (7, 8). In many high-income countries, asymptomatic carriage of this species in the upper respiratory tract is prevalent in adolescents, with a peak at 19 years of age and rates of 30% to 60% in some subpopulations (9). Disease rates are low (∼1 case/100,000), with infections mainly occurring in infants, except in sub-Saharan Africa, where attack rates during epidemics can reach 1 case/1,000. Lengths of host persistence of particular meningococcal clones range from a few weeks to >12 months (10). Natural transformation, facilitated by a *Neisseria*-specific 12-mer DNA uptake sequence (DUS), is the major determinant of interlocus or genome variation by HGT and is estimated to exceed *de novo* mutation by ∼10-fold (11). The ∼2-Mbp genomes of N. meningitidis contain ∼1 DUS/1,000 nucleotides but are also replete with other repetitive elements and multicopy genes (8). The latter includes four *opa* genes that are dispersed around the genome and loci containing arrays of complete and partial *maf* genes. The type IV pilus exemplifies a recombinatorial mechanism of LH with a G quadraduplex initiating high frequencies of recombination (gene conversion) between *pilE*, the expression locus for the major pilin, and multiple silent *pilS* genes encoding partial copies of this protein (12). Simple sequence repeats (SSR) are also highly prevalent in meningococcal genomes, with an average of 45 tracts per genome (13). These tracts are hypermutable due to a high propensity for slipped-strand mispairing during DNA replication and mediate changes in gene expression due to the SSRs being located in the reading frames or promoters of genes (7, 14). This process, referred to as phase variation (PV), can generate high levels of genetic variation through combinatorial switches in the expression of multiple genes. Thus, N. meningitidis has multiple mechanisms for rapid generation of genetic variation that are thought to support the adaptation and evolution of meningococci during within-host persistence of individual clones.

Herd immunity increases the protection and cost effectiveness of meningococcal immunization programs by reducing circulation of disease-causing strains. Recent introduction of a conjugate MenACWY vaccine into UK teenagers and university entrants aims at reducing infections by cutting circulation of hyperinvasive MenW strains (15). Similar ideas have been proposed for reducing circulation of MenB strains by immunization of teenagers and young adults with recombinant protein-based vaccines (16). However, utilization of vaccines in the major carriage population may perturb the meningococcal population structure and drive evolution of vaccine escape mutants, as observed with pneumococcal immunization programs (17, 18). To investigate the potential for within-host strain evolution, we have studied *de novo* mutation, recombination (gene conversion and HGT), and LH of meningococci during persistent meningococcal carriage in university students. This exploration of natural carriage provides the first comprehensive evidence that LH is the major driver of within-host evolution of this bacterial species and that directional genetic variation is biased toward genes affecting surface antigens.

## RESULTS

### Low levels of genic variation occur during persistent carriage of meningococci.

**FIG 1:**
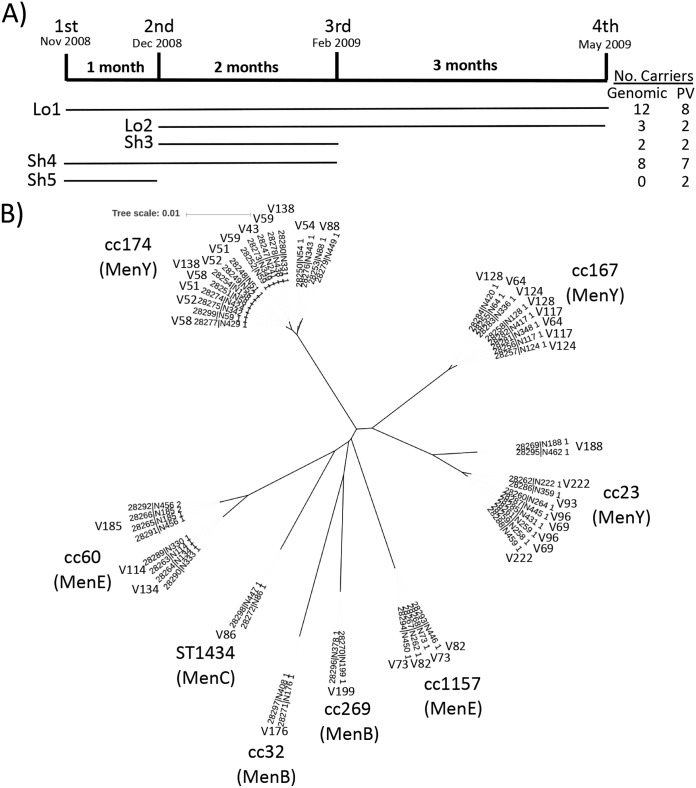
Core genome phylogenetic tree for 25 pairs of short- and long-term persistent carriage isolates. Persistent carriage isolates were chosen from volunteers, all first-year students that were part of a longitudinal carriage study at the University of Nottingham. For 25 volunteers, one isolate was chosen from the first and last time point where persistent carriage was observed and subject to WGS by Illumina HiSeq. Analysis of the PV states of 14 SSR-containing genes was also performed on multiple isolates for 21 volunteers. (A) Sampling times, the distribution of carriers with respect to long-term (Lo; 5 to 6 months) and short-term (Sh; 1 to 3 months) carriage, and the number of carriers whose isolates were subject to WGS (genomic) and/or PV analysis. (B) Phylogenetic tree derived using a subset of the *Neisseria* core genes (*n* = 215) and iTOL from whole-genome sequences generated on an Illumina HiSeq platform. The specific volunteers (see Table 1 for isolate names for each volunteer) in each group are: Lo1, V51, V58, V59, V73, V86, V88, V117, V128, V176, V185, V188, and V222; Lo2, V69, V82, and V96; Sh3, V43 and V93; Sh4, V52, V54, V64, V114, V124, V134, V138, and V199; Sh5, V113, and V115. Individual branches of the tree are labeled with the clonal complex (cc) and genogroup (capsule type). The volunteer for each isolate is also indicated (there is a single label where two or more isolates are on adjacent branches).

Although asymptomatic carriage is the natural state for N. meningitidis, there is a limited understanding of the levels of genetic variation associated with persistence in individual carriers. High-coverage genome sequences were generated using Illumina HiSeq for two isolates of the same strain from each of 25 carriers and representing 1 to 6 months asymptomatic carriage of a range of N. meningitidis clonal complexes (Fig. 1). Allelic variation within the coding regions of single-copy genes was detected in an average of five variable genes per carrier with an average genic variation rate per month of carriage of 7 × 10^−4^ (i.e., a meningococcus of ∼2,000 genes will accumulate 1.4 variable genes for each month of carriage) (Table 1). Only one difference (single nucleotide or indel) was detected in the majority of variable genes (69% [95/138]), suggestive of *de novo* mutation (Table 1). However, the two isolates from carrier V54 contained one nucleotide allelic differences in 24 genes, indicative of multiple recombination events (estimated to between 19 and 24) with a highly homologous meningococcus. If carrier V54 is excluded, then the *de novo* mutation rate is 1.02 mutations/genome/month of carriage, of which 19% was due to indels (18/94). These single nucleotide changes exhibited a 3:1 bias toward nonsynonymous changes, suggestive of either diversifying selection or insufficient time for purifying selection to remove mutations with reduced fitness.

**TABLE 1:** Allelic variation during persistent meningococcal carriage

Clonal complex	Volunteer no. (mo of carriage)^*a*^	Isolates (genomes)^*b*^	No. of genes		Mutability/mo of carriage (×10^−4^)	No. of genes	
			Total^*c*^	Variable^*d*^		With 1 difference (indel)	With >1 difference
cc174	V51 (6)	N51.1 (28248), N424.1 (28274)	1,854	3	2.7	3 (0)	0
	V43 (2)	N241.1 (28247), N349.1 (28273)	1,845	1	2.7	1 (1)	0
	V52 (3)	N52.1 (28249), N342.1 (28275)	1,856	0	0	0	0
	V58 (6)	N58.1 (28251), N429.1 (28277)	1,846	0	0	0	0
	V59 (6)	N59.1 (28252), N438.1 (28278)	1,855	4	3.6	4 (0)	0
	V88 (6)	N88.1 (28253), N449.1 (28279)	1,860	5	4.5	5 (2)	0
	V138 (3)	N138.1 (28254), N331.1 (28280)	1,865	4	7.1	4 (0)	0
	V54 (3)	N54.1 (28250), N343.1 (28276)	1,840	24	43.4	24 (2)	0
cc167	V64 (3)	N64.1 (28255), N348.1 (28281)	1,852	2	3.6	2 (1)	0
	V117 (6)	N117.1 (28256), N417.1 (28282)	1,850	4	3.6	4 (1)	0
	V124 (3)	N124.1 (28257), N336.1 (28283)	1,863	4	7.2	4 (1)	0
	V128 (6)	N128.1 (28258), N420.1 (28284)	1,846	34	30.7	9 (2)	25
cc23	V69 (5)	N258.1 (28259), N431.1 (28285)	1,820	0	0	0	0
	V93 (2)	N264.1 (28260), N359.1 (28286)	1,816	6	16.5	6 (1)	0
	V96 (5)	N259.1 (28261), N445.1 (28287)	1,833	7	7.6	7 (3)	0
	V222 (6)	N222.1 (28262), N459.1 (28288)	1,732	8	7.7	1	7
	V188 (6)	N188.1 (28269), N462.1 (28295)	1,863	0	0	0	0
cc60	V114 (3)	N114.1 (28263), N330.1 (28289)	1,797	8	14.8	7 (1)	1
	V134 (3)	N134.1 (28264), N333.1 (28290)	1,791	2	3.8	1 (0)	1
	V185 (6)	N185.1 (28265), N185.2 (28266), N456.1 (28291), N456.2 (28292)	1,823	5	4.6	4 (1)	1
cc1157	V73 (6)	N73.1 (28268), N450.1 (28294)	1,838	3	2.7	3 (0)	0
	V82 (5)	N262.1 (28267), N446.1 (28293)	1,843	10	9.0	2 (0)	8
cc269	V199 (3)	N199.1 (28270), N378.1 (28296)	1,947	1	1.7	1 (1)	0
cc32	V176 (6)	N176.1 (28271), N408.1 (28297)	1,974	1	0.8	1 (1)	0
	V86 (6)	N86.1 (28272), N447.1 (28298)	1,794	2	1.9	2 (0)	0

a Note that each pair of isolates is of the same sequence type and that carriage spanned between 2 and 6 months.

b Isolate number (number allocated to the genome sequence of that isolate in the PubMLST/neisseria database).

c Total number of analyzed genes excluding truncated genes (average of 41/genome) and duplicated or multicopy genes (e.g., insertion elements [IS], *pilE*/*pilS*, and multigene families).

d Gene sequences having one or more nucleotide differences between the two paired isolates.

We also observed a relatively high occurrence of HGT with three carriers (V82, V128, and V222) containing 93% (40/43) (Table 1) of the variable genes with >1 difference per coding region, suggesting that HGT between meningococci of different clonal complexes (ccs) occurred in 12% of carriers (3/25). The putative number of HGT events/carrier for V82, V128, and V222 ranges between 3 and 10 with an average recombination patch size of 2,905 bp (see https://leicester.figshare.com/s/24e2782f537d2c77af60). Another strain (N292.1, ST823, cc198) was isolated at an intermediate time point from carrier V128 and shown to be the donor for HGT into the cc167 isolates (N128.1 and N420.1) from this carrier (see https://leicester.figshare.com/s/24e2782f537d2c77af60). For V82, all recombination fragments had 100% homology to sequences from N64.1 (present in carrier V64), indicating that a cocolonizing cc167 strain was the most likely donor strain to the cc1157/MenE strains of this carrier (see https://leicester.figshare.com/s/24e2782f537d2c77af60). For V222, the recombination fragment carrying *tbp* sequences had very high homology to sequences from cc23 isolates obtained from carriers in 2015 and 2016 or patients in 2011 and 2012 (19, 20), whereas homologous sequences in other strains could not be found for the other fragments (see https://leicester.figshare.com/s/24e2782f537d2c77af60). The average synonymous-to-nonsynonymous mutation (dS/dN) ratio for the genes with multiple allelic differences was 10.1, indicating that sequences transferred by HGT have been subject to purifying selection in the donor genomes.

### No differences between functional groups in the extent of allelic variation.

No significant differences were detected in nucleotide diversity for variable genes with nonsynonymous allelic variation among five broad functional groups (see Fig. S1 in the supplemental material). There was a significant deviation in the numbers of variable membrane-associated genes for all carriers and for mutation-only carriers (Table 2), suggesting that the mutations were occurring by chance within genes of greater than the average length for this functional group. There was also a lower level of significance for mutation only for genes involved in DNA metabolism. A statistically significant association was detected for indels in genes with membrane-associated functions (8/17; *P* value < 0.0001) with three carriers having identical 15-nucleotide deletions in *porB*, encoding a major outer membrane protein (OMP). Many indels were large (9 to 33 bp), suggestive of evolved indel-susceptible sequences in these genes. No significant differences were detected in genic variability between short (1 to 3 months) versus long (>3 months) persistent carriage; however, there was a significant difference for the four isolate pairs with putative HGT versus the other 21 strain pairs (Fig. S1), indicating that within-host HGT, but not host persistence *per se*, is the main driver of allelic variation. A high proportion (39% [14/36]) of the HGT events were associated with allelic variation in genes encoding an OMP or a surface-modifying enzyme (e.g., pilin glycosyl transferase), indicating a bias in selection for fixation of HGT events involving modifiers of surface-exposed molecules.

**TABLE 2:** Distribution of variable genes with nonsynonymous allelic variation by function^*a*^

Functional group (no. in class per genome)	No. (%) of variable genes^*b*^		Nucleotide diversity (×10^−5^)^*b*^^,^^*d*^		
	All carriers	Without HGT^*c*^	All carriers	Without HGT	HGT only
Membrane associated (180)	25 (0.56)^*e*^	12 (0.32)^*e*^	5.1^*f*^	0.19^*f*^	27.5^*f*^
Metabolic functions (663)	31 (0.19)^*f*^	12 (0.07)^*f*^	2.0^*f*^	0.12^*f*^	11.9^*f*^
Regulation (325)	8 (0.1)^*f*^	3 (0.04)^*f*^	2.0^*f*^	0.08^*f*^	11.8^*f*^
DNA metabolism (104)	7 (0.27)^*f*^	6 (0.23)^*g*^	0.2^*f*^	0.16^*f*^	0.1^*f*^
Unknown function (855)	39 (0.18)^*f*^	13 (0.06)^*f*^	3.1^*f*^	0.22^*f*^	13.8^*f*^
Total (2,127)	110 (0.21)	46 (0.09)	2.3	0.15	12.0

a A list of variable genes with nonsynonymous changes (i.e., with an altered amino acid sequence) was derived by analysis of alignments of derived amino acid sequence for each variable gene detected in the 25 pairs of persistent meningococcal isolates (Table 1). These genes were classified into one of five functional groups based on known functions or a homology-based analysis. Variable genes were also separated by occurrence in a carrier with or without high levels of HGT, with these classifications predicted to separate genes whose variation occurred by recombination from mutation.

b The denominator for calculating the percentage of variable genes was determined by multiplying the total number of genes in the class by the number of carriers.

c Numbers of carriers in each group: all carriers, *n* = 25; without HGT, *n* = 21; HGT only, *n* = 4 (V54, V82, V128, and V222).

d Nucleotide diversity was calculated by using the average diversity for all genes to estimate the number of variants in a gene of 1,000 bp and then dividing this number by the total length of genes in a class (calculated by multiplying the number of genes by 1,000).

e *P*, <0.001; chi-square test of observed versus expected.

f Not significant by a chi-square test of observed versus expected for the number of variable genes or in one-way analysis of variance (ANOVA) for nucleotide diversity.

g *P*, <0.01, chi-square test of observed versus expected.

10.1128/mBio.03068-19.2 FIG S1 Comparison of the nucleotide diversity of genes with nonsynonymous and synonymous allelic diversity between paired meningococcal carriage isolates representing 2 to 6 months persistent asymptomatic carriage in one individual. Variable genes were identified by comparison of genic sequences. Genes were examined for differences in amino acid sequences and separated into those with 1 or more nonsynonymous differences (A) or only synonymous differences (B). Genes were then separated into one of four functional gene classes. Nucleotide diversity was calculated by dividing the numbers of differences in a gene by the total gene length, and an average was calculated for all variable genes in that class. This average number was then adjusted to account for the number of genes in each functional class and number of carriers analyzed. The average nucleotide diversity is presented for various classifications: all, all 25 pairs of isolates; all (no HGT), 21 carriers where no horizontal gene transfer was observed; all (only HGT), the four carriers were HGT was observed; short, 10 carriers representing 2 to 3 months persistent carriage; long, 15 carriers representing 5 to 6 months persistent carriage. Download FIG S1, PDF file, 0.2 MB. Copyright © 2020 Green et al. 2020 Green et al. This content is distributed under the terms of the Creative Commons Attribution 4.0 International license.

### Allelic variation within a meningococcal carriage population.

**FIG 2:**
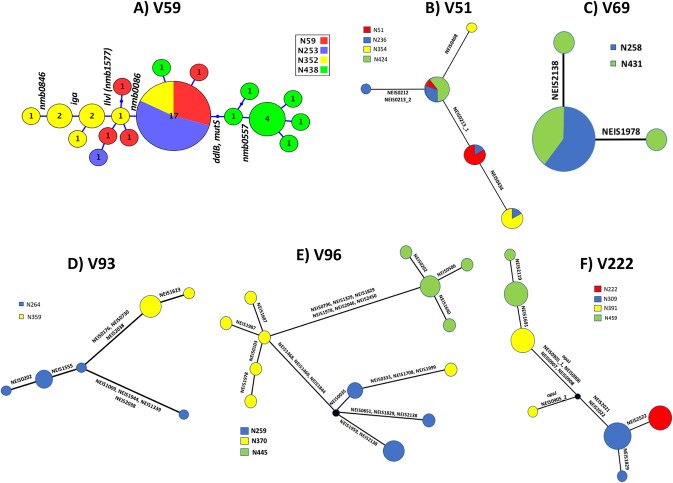
Whole-genome variation during persistent carriage. Allelic variation in coding regions was derived from the whole-genome sequences of 12 to 39 isolates per carrier for six carriers. The distribution of the allelic variation and relatedness of isolates was visualized using GrapeTree and manual manipulation of branch positions. Isolates are color coded for time point of isolation (Fig. 1): red, first; blue, second; yellow, third; green, fourth. Genes subject to variation between each node are indicated using gene names or NEIS numbers (see https://pubmlst.org/neisseria/) with alternate types of variation in the same gene indicated by a low dash and number. The clonal complexes of isolates were either cc174 (V51 and V59) or cc23 (V69, V93, V96, and V222).

To examine within-time-point allelic variation, whole-genome sequencing (WGS) was performed on 9 to 10 isolates/time point for carrier V59. Allelic variation was detected in 16 genes, of which seven occurred in only one isolate (see https://leicester.figshare.com/s/24e2782f537d2c77af60). A network diagram shows how partial sweeps of 90% (i.e., 9/10 colonies) were observed for variants in two genes (*ddlB* and *mutS*) in the fourth time point and sweeps of 60 to 70% for three other genes in the third and fourth time points, while one genotype, or near-identical variant thereof, persisted across all 6 months of carriage (Fig. 2A).

WGS on multiple colonies for five other carriers detected similar levels of allelic variation, with two showing stable persistence of an identical core-genome genotype for 5 to 6 months of carriage and transient appearance of variants (Fig. 2B to F). Two carriers (V96 and V222) exhibited evidence of directional changes in multiple genes (Fig. 2E and F). For V96, there was deletion of two genes (*NEIS1464* and *NEIS1469*) encoding the lactoferrin binding protein combined with allelic changes in *pilF*, followed by subsequent, concomitant single-nucleotide changes in five other genes with a variety of functions. In V222, there were three HGT events involving multiple genes, of which two were linked to variations in OMPs (i.e., OpaJ and TbpB, part of the transferrin binding protein). Allele-specific PCR on a further 12 variable genes for eight carriers (see https://leicester.figshare.com/s/24e2782f537d2c77af60) identified seven variant alleles occurring in only one isolate and four variant alleles in all isolates (*n* = 6) for either an intermediate or final time point. Examination of five putative HGT events in V128 indicated that only three were associated with sweeps for all isolates of the final time point. Finally, the 15-bp deletion in PorB produced near complete sweeps in all three carriers where this mutation was observed after 3 months of persistent carriage.

These results indicated that allelic variation was split into four forms (Table 3): (i) sporadic, i.e., occurring in only 1 tested isolate of 6 or more, 43% (35/82 variable genes); (ii) transient, i.e., occurring at an intermediate time point or only in a subset of isolates, 17% (14/82); (iii) putative fixation, i.e., occurring in all isolates of the final observed time point, 27% (22/82); and (iv) potential fixation, i.e., the majority of isolates of the final two time points, 13% (11/82). Of the latter category, three encode OMPs (PorB, LbpA, and LbpB), four are on a recombination fragment associated with allelic variation of an Opa protein in strain V222, and another encodes a pilin modifier (PilF); thus, 73% (8/11) of the “fixed” variation causes alterations in surface molecules, suggesting that adaptive variation is focused on surface structures during persistent meningococcal carriage.

**TABLE 3:** Analysis of the types and distribution of allelic variation observed for multiple meningococcal isolates and multiple time points during persistent carriage

Allelic variation type^*a*^	Incidence	Variable genes (carriers)
Sporadic	35	*NEIS0212* (V51), *pilQ* (V51), *pglA* (V51), *uvrA* (V54), *gltX* (V59), *rpoC* (V59), *NMB0830* (V59), *NMB1114* (V59), *NMB122*0 (V59), *sspA* (V59), *polA* (V59) *mutS* (V69), *NEIS1978* (V69), NEIS1339 (V93), NEIS0202 (V93), NEIS1009 (V93), *pabBC* (V93), *NEIS1623* (V93), *NEIS0202* (V96), *secG* (V96), *iga* (V96) *mafB1* (V96), *NEIS1640* (V96), *pldA* (V96), *NEIS1708* (V96), *hrpA* (V96), *yccA* (V96), *NMB0669* (V114), *ligA* (V114), *NMB0409* (V117), *NMB1288* (V138), *NMB0424* (V138), *pilG* (V138), *tspA* (V222), *NEIS2119* (V222)
Transient	14	*pilU* (V59, V96), *NEIS007*1 (V59), *iga* (V59, V96), *clpA* (V59), *NMB0846* (V59), *ilvL* (V59), *MTFMT* (V96), *mutS* (V96), *NMB1485* (V114), *NMB0114* (V128), *suhB* (V128), *NMB0274* (V138)
Putative fixation	22	*argC* (V51), *argF* (V54), *ddl* (V59), *NEIS0498* (V59), *mutS* (V59), *porB* (V64, V124), *NEIS0176* (V93), *hemD* (V93), *NEIS1555* (V93), *rplM* (V93), *NEIS0796* (V96), *NEIS1329* (V96), *tspA* (V96), *NEIS1978* (V96), *hemK* (V96), *NEIS2450* (V96), *NMB0920* (V114), *dnaB* (V117), *NMB0439* (V128), *aspC* (V128), *tbpB* (V222)
Potential fixation	11	*lbpA* (V96), *lbpB* (V96), *pilF* (V96), *porB* (V117), *pip* (V222), *bamC* (V222), *dapA* (V222), *NEIS0908* (V222), *thiC* (V222), *NEIS2022* (V222), *NEIS2522* (V222)

a Sporadic, occurring in only 1 tested isolate of 6 or more; transient, occurring at an intermediate time point or only in a subset of isolates; putative fixation, occurring in all isolates of the final time point; potential fixation, occurring in the majority of isolates of the final two time points.

### Extensive intragenomic recombination occurs in the major pilin subunit but not the multicopy *opa* genes.

**FIG 3:**
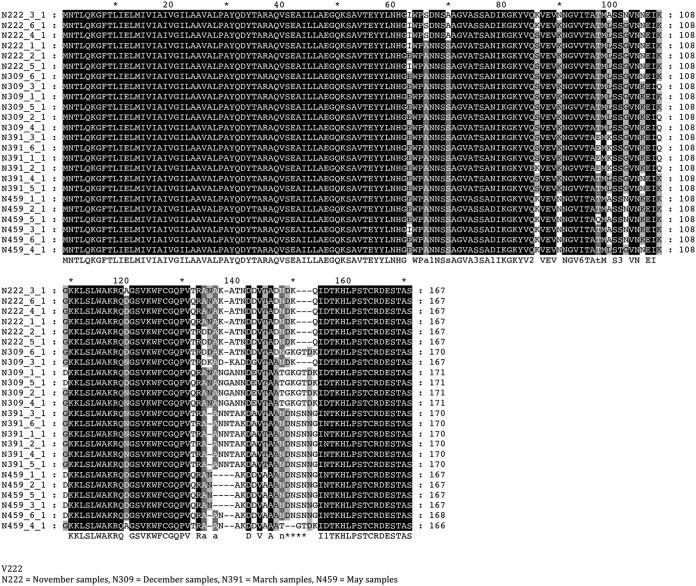
High-level temporal and spatial allelic variation in the meningococcal PilE protein deduced from *pilE* genes. An alignment of the amino acid sequences of the PilE protein for 24 isolates from carrier V222 are shown. Sequences were derived by Sanger sequencing of PCR products spanning the *pilE* gene. Isolates were derived from four different time points representing up to 6 months of carriage, with 6 isolates per time point (Fig. 1).

Variation in the hypervariable *pilE* gene was examined for multiple carriers by either bioinformatic comparisons of WGS data or sequencing of PCR products encompassing the *pilE* gene. No allelic variation was observed in *pilE* of cc174 isolates due to separation of the *pilE* gene and G quartet into different genomic regions. All other strains exhibited allelic variation in *pilE*, even after only 1 month of carriage (see Table S1). Analysis of the nucleotide and amino acid sequences of multiple isolates per time point from two carriers (V117 and V222) indicated that every time point contained multiple PilE alleles and that complete switches in the PilE type between two time points were frequent (Fig. 3; see also Fig. S2). These data clearly demonstrate that allelic variation of PilE occurs at very high frequencies during persistent meningococcal carriage, as previously observed for gonococcal infections (21).

10.1128/mBio.03068-19.1 TABLE S1 Allelic variation in PilE amino acid sequences for longitudinal carriage isolates. Download Table S1, DOCX file, 0.03 MB. Copyright © 2020 Green et al. 2020 Green et al. This content is distributed under the terms of the Creative Commons Attribution 4.0 International license.

10.1128/mBio.03068-19.3 FIG S2 Allelic variation in the derived PilE amino acid sequences of multiple meningococcal isolates from a single carrier. The *pilE* gene was amplified from 19 meningococcal carriage isolates obtained from volunteer V117. The isolates were obtained at the first (2, N117), second (6, N284), third (5, N332), and fourth (6, N417) time points and represent initial, 1, 3, and 6 months persistent carriage. Gene sequences were translated and aligned using Clustal Omega. Download FIG S2, PDF file, 2.1 MB. Copyright © 2020 Green et al. 2020 Green et al. This content is distributed under the terms of the Creative Commons Attribution 4.0 International license.

Intragenomic recombination in the multicopy *opa* genes is presumed to occur by RecA-mediated recombination (22). Genomic sequences were utilized to locate and derive specific primers for each *opa* gene. Four Opa-containing loci (termed *opaA*, *opaB*, *opaD*, and *opaJ*, herein) (Table 4) were found in every isolate, with multiple isolates having one or more identical Opa alleles (A. A. Al-Rubaiawi, unpublished data). Only one pair of *opa* loci in one isolate had undergone intragenomic recombination (A. A. Al-Rubaiawi, unpublished), while another had undergone gene conversion (V222) (A. A. Al-Rubaiawi, unpublished).

**TABLE 4:** PV gene modules

Module	Genes
LOS	*lgtG* (*NEIS2011*), *NMB1255*^*a*^
Pilin modification	*pglA* (*NEIS0213*), *pglE* (*NEIS0568*), *pglH* (*NEIS0400*), *pglI* (*NEIS0380*)
Pilin modulation	*pilC1* (*NEIS0371*)*, pilC2* (*NEIS0033*)
Multicopy OMP-Opa	*opaA* (*NMB0442*), *opaB* (*NMB1636*), *opaD* (*NMB1465*), *opaJ* (*NMB0926*)
Single-copy OMPs^*b*^	*fetA* (*NEIS1963*), *hmbR* (*NEIS1586*), *hpuA* (*NEIS1946*), *mspA* (*NEIS1974*), *nadA* (*NEIS1969*), *nalP*, *opc* (*NEIS2198*), *porA* (*NEIS1364*)
RM	*modA* (*NEIS1310*), *modB* (*NEIS1194*)

a NMB, gene identifier for the MC58 genome sequence.

b Data reported previously by Alamro et al. (23).

### Variable frequencies of switching in pilin, LOS, and RM phase variation modules during persistent meningococcal carriage.

Previous analyses had detected frequent PV of eight single-copy (sc) phase-variable OMPs (23, 24). These studies were extended to another 14 phase-variable genes in five PV modules whose PV is controlled by SSRs located within the reading frame (Table 4). These phase-variable genes were selected as major components of the N. meningitidis core phasome (13) and as genes with a known or high propensity to undergo PV (i.e., we excluded genes with only 3 to 4 repeats). The colocated *lgt* genes (e.g., *lgtA*, *lgtB*, etc. [25]) were not analyzed due to problems with designing repeat-specific primers for these highly homologous genes.

**FIG 4:**
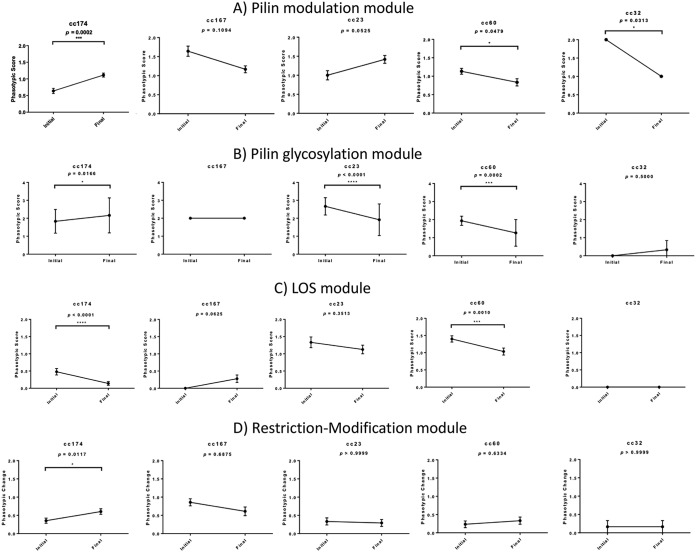
Combinatorial changes in PV modules during persistent meningococcal carriage. Repeat numbers were derived for each phase-variable gene by fragment analysis of PCR products spanning the relevant repeat tract. Expression states were derived from the repeat numbers and associations between repeat number and translational state (“on” and “off” coded as 1 and 0, respectively). Individual phasotypic scores for the initial and final point of observed carriage for each carrier were derived from analysis of ∼6 isolates per time point. Average phasotypic scores and standard deviations were derived from the following numbers of carriers for each clonal complex: 8, cc174; 3, cc167: 4, cc23; 5, cc60; and 1, cc32. Statistical differences between the initial and final times were determined using a Wilcoxon rank sum test in Prism. The PV modules consisted of the following genes: (A) pilin modulation, *pilC1* and *pilC2*; (B) pilin glycosylation, *pglA*, *pglE*, *pglH*, and *pglI*; (C) LOS, *lgtG* and *NMB1255*; (D) restriction modification, *modA* and *modB*. Note that the maximum phasotypic score occurs when all genes within a module are in an “on” state (thus a module of four phase-variable genes has a maximum score of 4).

The pilin modulation module consists of two highly homologous *pilC* genes whose PV is mediated by poly(C) tracts located close to the ATG initiation codon (26) and encode large 110-kDa proteins that modulate type IV pilus surface expression (27). Using unique PCR primers for each *pilC* gene, repeat numbers were found to range from 8 to 15 with a modular length of 10 for both genes (see Data File S1), while the average mutation rate was 0.085 mutations/gene/month. Unexpectedly, some cc174 strains had a second poly(C) tract of 10 to 14 repeats located in the central portion of *pilC1*. Following conversion of repeat numbers into predicted expression states, we observed that 62% and 24% of isolates express one or two PilC proteins, respectively, with a significant increase in expression during persistent carriage in cc174 isolates but stable or decreasing expression in other ccs (Fig. 4A). An important trend was for switching toward PilC2-only expression states in four of the lineages as a function of persistent carriage (see Fig. S3). Notably, five carriers exhibited some degree of opposing switching of the two *pilC* genes, while there was an overall switching rate of 31% for this module (i.e., 13/42 *pilC* genes exhibited an 80% to 100% switch in expression state) (Fig. S3). Variation within many of the time points, as measured using a Shannon factor-based diversity score, was between 0.2 and 0.7, suggesting that these changes are due to a mix of random drift and selection (Fig. S3).

10.1128/mBio.03068-19.4 FIG S3 Phasotypes for the pilin modulation module. (A) Proportions of isolates containing different combinations of expression of the *pilC1* and *pilC2* phase-variable genes (1 and 0 indicate “on” and “off” states, respectively). Percentages were calculated from total number of isolates analyzed at each time point: cc174, 47, 39, 37, 23; cc167, 14, 18, 18, 6; cc23, 6, 24, 18, 18; cc60, 30, 30, 12, 6; cc32, 6, 6, 6, 6. (B) Change in expression for *pilC1* and *pilC2* in each carrier between the initial and final time point. Values of 1 and 0, all observed isolates in “on” or “off” state, respectively. (C) Shannon diversity scores. Diversity scores were calculated from the combined expression scores of the *pilC1* and *pilC2* genes for multiple isolates per time point; 1, all isolates were different from each other; 0, all isolates had the same score. Download FIG S3, PDF file, 0.4 MB. Copyright © 2020 Green et al. 2020 Green et al. This content is distributed under the terms of the Creative Commons Attribution 4.0 International license.

**FIG 5:**
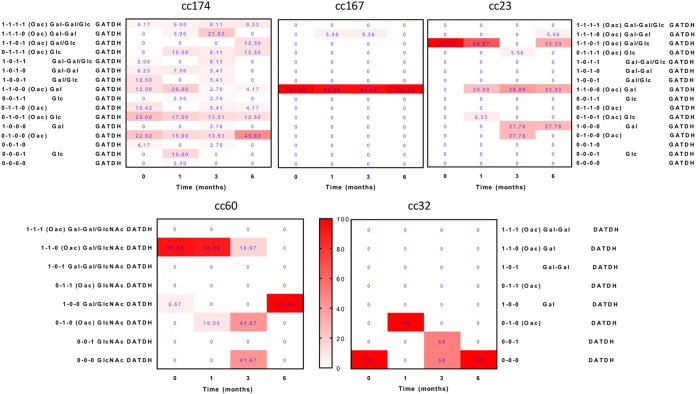
Putative phenotypic effects of phase-variable changes in the pilin glycosylation module. The longitudinal changes in the proportions of carriage isolates associated with the putative glycan structures of each phasotype are shown. The four-gene phasotype represents the expression states of the *pglA*, *pglI*, *pglE*, and *pglH* genes, the products of which are responsible for the addition of the first galactose, an acetyl group, a second galactose, and a glucose moiety, respectively, to the basal sugar. Addition of the second galactose by PglE requires prior addition of the first galactose by PglA, while PglH competes with PglA for addition of glucose to the same acceptor position. The basal sugar, GATDH or DATDH, is determined by PglB2 or PglB, respectively, and differs among clonal complexes. The percentage values were calculated by determining the number of isolates across multiple carriers with a specific phasotype and dividing by the total number of analyzed isolates for each time point. Boxes are colored according to the heat map shown by the bar between the lower panels and ranges from white (0% of isolates) to bright red (100% of isolates). Note that the cc60 and cc32 meningococci lacked the *pglH* gene, resulting in a three-gene phasotype for these lineages. The total numbers of isolates analyzed for each clonal complex for the four time points were as follows: cc174 (48, 40, 37, 24); cc167 (14, 14, 18, 6); cc23 (6, 24, 18, 18); cc60 (30, 30, 12, 6); and cc32 (6, 6, 6, 1).

For the pilin glycosylation module, we analyzed four phase-variable genes (*pglA*, *pglE*, *pglI*, and *pglH*) whose products vary glycans on the type IV pilus and other surface proteins between three different glycan extensions with and without acetylation (28). Initiating glycan residues were predicted to vary, as isolates have either a *pglB* or *pglB2* gene (see reference 29) (Fig. 5). PV is controlled by a heptanucleotide repeat in *pglE* and by poly(G) tracts in the other genes. Phasotypes and linked phenotypes, derived from analysis of expression states, were confirmed for a subset of isolates by immunoblotting with glycan-specific antibodies (see Fig. S4). Comparative analyses indicated that all 16 possible phasotypes were detected in the eight carriers of cc174 isolates but only 2 to 7 phasotypes for the 13 carriers of cc23, cc167, cc60, and cc32 isolates, with the 1-1-0-0 and 1-1-0-1 phasotypes dominating in the early time points (Fig. 5). This diversity in cc174 isolates was due to differences between carriers and time points, as diversity within individual time points was low (see Fig. S5). Persistent carriage was associated with differential patterns of behavior; combinatorial *pgl* phasotypic scores exhibited significant increases in cc174 isolates but decreases in cc23 and cc60 isolates (Fig. 4B). The cc174 phenotypic pattern was complex (Fig. 5); thus, Gal-Gal-containing phasotypes rose over the first 3 months from 12% to 42% before falling back to 8% after a further 3 months of carriage. This fall coincided with a switch to glycoforms lacking Gal moieties (0-1-0-0; 46% isolates) or having a Glu extension (0-1-1-1 and 0-1-0-1; 24%). An explanation of this pattern is that the diGal motif initially masks an antigenic epitope and then becomes the main antigenic target within this clonal complex. Notably, the Gal-Gal extension produced by the combined action of *pglA* and *pglE* (phasotypes 1-1-1-1, 1-1-1-0, 1-0-1-1, and 1-0-1-0) was rarely observed, occurring in 0% to 6% of isolates across the four time points for the cc167/cc23/cc60/cc32 isolates (Fig. 5). These findings suggest that there is selection against this extended glycan form in most carriers.

10.1128/mBio.03068-19.5 FIG S4 Western blot to determine the expression states of a subset of persistent carriage isolates. Whole-cell lysates were prepared from 15 meningococcal isolates obtained as part of this study. Lysates were subject to separation on a PAGE gel and then transferred to a nitrocellulose membrane. These membranes were probed with monoclonal antibodies specific for a range of different glycan extensions. Download FIG S4, PDF file, 0.4 MB. Copyright © 2020 Green et al. 2020 Green et al. This content is distributed under the terms of the Creative Commons Attribution 4.0 International license.

10.1128/mBio.03068-19.6 FIG S5 Shannon diversity scores for each time point for the pilin glycosylation module. The genes in this module and method for calculation of expression score is indicated in Fig. 5. The diversity was calculated as described for Fig. S3. Download FIG S5, PDF file, 0.1 MB. Copyright © 2020 Green et al. 2020 Green et al. This content is distributed under the terms of the Creative Commons Attribution 4.0 International license.

For the LOS PV module, we focused on two genes, *lgtG* and *NMB1255*. Repeat tracts ranged between 7 and 16 for these genes (Data File S1). The cc23 and cc60 isolates exhibited higher levels of “on” expression states than other isolates (see Fig. S6). A significant decrease in “on” expression states of these genes in cc174 and cc60 carriers is indicative of selection against certain immunotypes (Fig. 4). PV of *NMB1255* was higher than for *lgtG*, while diversity within time points was generally low, suggesting that these LOS epitopes are subject to infrequent but strongly selective shifts in expression (see Fig. S6).

10.1128/mBio.03068-19.7 FIG S6 Phasotypes for the LOS module (*lgtG* and *NMB1255*). (A) Percentages were calculated as described for Fig. S3 for the following numbers of isolates: cc174, 48, 40, 37, 23; cc167, 14, 18, 18, 15; cc23, 6, 24, 18, 18; cc60, 30, 30, 12, 6; cc32, 6, 6, 6, 6. (B) Switching states for each volunteer for the LOS module. See the legend for Fig. S3 for method for determination of expression states. (C) Shannon diversity scores for each time point. See the legend for Fig. S3 for how diversity scores were derived. Download FIG S6, PDF file, 0.2 MB. Copyright © 2020 Green et al. 2020 Green et al. This content is distributed under the terms of the Creative Commons Attribution 4.0 International license.

An unexpected finding for PV was of phase-variable restriction modification (RM) systems (14, 30). The cc174 isolates contained phase-variable *modA* and *modB* genes, while only the *modA* gene of cc167 and cc23 isolates contained an SSR. Both tetra- and pentanucleotide SSRs were present and contained 3 to 37 repeats (Data File S1). Phase-variable *mod* genes were in “off” states in most isolates, with only cc174 isolates exhibiting a significant shift in expression (Fig. 4D). However, a complete shift in expression of four *mod* genes (4/31 [13%]) (see Fig. S7) is suggestive of strong but infrequent selection for switches in expression of these genes. Conversely, high levels of heterogeneity in expression states (i.e., a Shannon diversity score of >0.4) were observed at multiple time points for several carriers (Fig. S7). Heterogeneity may arise due to selection for mixed populations or as result of high mutability in SSRs combined with nonspecific bottlenecks or mutational drift.

10.1128/mBio.03068-19.8 FIG S7 Phasotypes for the Mod module (*modA* and *modB*). (A) Percentages were calculated as described for Fig. S3 for the following numbers of isolates: cc174, 48, 40, 37, 24; cc167, 14, 18, 18, 6; cc23, 6, 24, 18, 18; cc60, 30, 30, 12, 6; cc32, 6, 6, 6, 5. (B) Switching states for each volunteer for the Mod module. See the legend for Fig. S3 for how expression states were determined. Note that the cc167 and cc23 strains did not contain the *modB* gene. (C) Shannon diversity scores. See the legend for Fig. S3 for how diversity scores were derived. Download FIG S7, PDF file, 0.2 MB. Copyright © 2020 Green et al. 2020 Green et al. This content is distributed under the terms of the Creative Commons Attribution 4.0 International license.

### Persistent meningococcal carriage is associated with frequent directional switching in the multicopy *opa* genes.

Pentanucleotide repeats of five or more were found in 83% (63/76) of *opa* genes (Data File S1). Multiplex PCRs/GeneScan assays were designed to detect variation in SSRs of all four Opa proteins (A. A. Al-Rubaiawi, unpublished). The average Opa mutation rate per month of carriage was 0.11 with variability increasing with repeat number: 0.007 for ≤5, 0.09 for 6 to 8, and 0.16 for ≥9 (https://leicester.figshare.com/s/24e2782f537d2c77af60).

**FIG 6:**
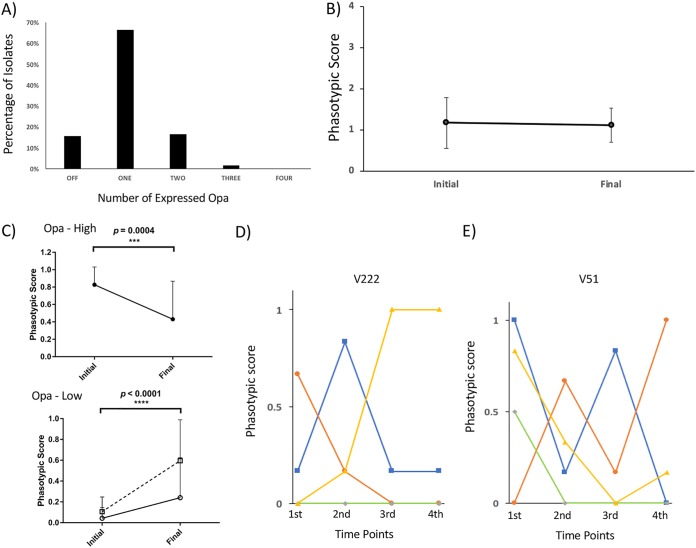
Directional and opposing PV of Opa proteins during longitudinal meningococcal carriage. The expression states of the Opa proteins were derived from analyses of gene sequences and pentanucleotide repeat numbers for all four loci of each isolate. (A) Percentages of 392 isolates from 19 carriers that had an “on” expression state in none (“off”) to one or more loci. (B) Average phasotypic scores for the Opa module for the initial and final time points of observed carriage for these carriers (note that the maximum score is 4; error bars are standard deviations). (C) Change in phasotypic scores for individual *opa* genes across multiple carriers for loci starting with a high level of isolates in either an “on” or “off” state. Loci were split by the overall expression state in the initial time point for each carrier into three categories: (i) 50% or more of isolates in the “on” state (Opa-high, 24 loci) (top); (ii) <50% of isolates in the “on” state (Opa-low, black line/open circles, 52 loci) (bottom); (iii) <50% of isolates in the “on” state but excluding carriers where there was no change in expression between the initial and final time points (Opa-low, dotted line/open squares, 21 loci). (D and E) Changes in expression of individual Opa loci for multiple time points of two carriers. Phasotypic scores are the averages from multiple isolates per time point. Blue line, OpaA; orange line, OpaB; green line, OpaD; yellow line, OpaJ.

Genome sequence analysis and resequencing of each SSR/flanking sequence enabled association of repeat number to expression state and derivation of four-gene Opa phasotypes (see https://leicester.figshare.com/s/24e2782f537d2c77af60). Variable patterns were detected between Opa alleles in a run of C and T residues located between the ATG and SSR plus insertion/deletion of a codon on either side of the repeat tract. Expression states for the phasotypes were confirmed by Western blotting using an Opa antiserum specific for a conserved domain (see Fig. S8). The phasotypes and individual gene expression states were examined for carriage-associated patterns. Expression of a single Opa was the dominant pattern occurring in 66% (260/392) of isolates (Fig. 6A). Contrastingly, only 16% of isolates did not express any Opa proteins, and no isolates exhibited expression of all four Opa proteins (Fig. 6A). Examination of phasotypic scores for four-gene Opa phasotypes indicated that expression of a single gene was maintained during persistent carriage (Fig. 6B). However, there was significant switching from low to high and high to low average Opa expression states (Fig. 6C). Furthermore, opposing switches in expression of individual Opa genes (i.e., “on” to “off” in one locus and “off” to “on” in another locus) were observed in 63% (12/19) of carriers. Two examples of opposing switching are for V222 wherein OpaJ switches “on” while OpaB switches “off” over 6 months carriage (Fig. 6D), whereas in V51 OpaA and OpaJ switch “off” while OpaB switches “on” (Fig. 6E). These overall and individual PV patterns are indicative of immune selection against an Opa variant(s) expressed by initial colonizing isolates accompanied by selection for expression of alternate functionally equivalent Opa alleles in order to maintain an adhesion phenotype.

10.1128/mBio.03068-19.9 FIG S8 Confirmation of *opa* gene expression states by Western blotting. Proteins from whole-cell lysates of carriage isolates of isolates with different repeat numbers and predicted expression states for the *opa* genes were separated by electrophoresis on 12% SDS-PAGE gels and transferred to polyvinylidene difluoride (PVDF) membranes. These membranes were probed with primary antibodies as follows: rabbit polyclonal anti-Opa antibodies, mouse monoclonal anti-PorA antibodies (cc174, P1.16; cc60, P1.2; cc167, P1.5), and mouse polyclonal anti-fHbp V1 antibodies. Numbers on the Opa blots refer to the repeat number of the four *opa* genes in the order *opaA*-*opaB*-*opaD*-*opaJ*, with red and black indicating genes in the phase “on” and “off” states, respectively. (A) cc174 isolates: lane 1, N59.3; 2, N54.1; 3, N369.1; 4, N88.1; 5, N352.2; 6, N424.1; 7, N354.1; 8, N51.1; 9, N138.1; 10, N272.1; 11, N253.1. (B) cc60 isolates: lane 1, N114.1; 2, N333.1; 3, N330.1; 4, N113.1; 5, 134.2. (C) cc167 isolates: lane 1, N332.2; 2, N348.1; 3, N117.1; 4, N284.1. (D) cc23 isolates: lane 1, N309.1; 2, N222.1; 3, N459.6; 4, N370.4; 5, N370.5. Download FIG S8, TIF file, 0.8 MB. Copyright © 2020 Green et al. 2020 Green et al. This content is distributed under the terms of the Creative Commons Attribution 4.0 International license.

10.1128/mBio.03068-19.10 DATA FILE S1 This file contains lists of the phase variation and GeneScan data for all isolates for pilin modulation, pilin glycosylation, restriction modification, and Opa modules of phase-variable genes. The following information is listed for every gene for each isolate: size of PCR product as determined by GeneScan, derived repeat number (as determined from GeneScan relative to a product of known repeat number), and derived expression state (as determined from a bioinformatics analysis of the open reading frame or relative to other isolates of known expression state). Two additional items of information are provided for the Opa genes: size of G tract and size of A tract (these two tracts are adjacent to the repetitive sequence and influence the expression state). Download DATA SET S1, PDF file, 2.4 MB. Copyright © 2020 Green et al. 2020 Green et al. This content is distributed under the terms of the Creative Commons Attribution 4.0 International license.

## DISCUSSION

Mutators, genetic exchange, and LH have evolved in several bacterial species in response to specific pressures encountered during persistence in or transmission between host organisms (5, 31, 32). To examine the relative contributions of different mutational processes to asymptomatic carriage, we analyzed genetic variation in multiple meningococcal isolates obtained from persistently colonized asymptomatic carriers. Samples were from a University of Nottingham carriage study and hence are representative of natural carriage of this species in the age group that is associated with the highest rates of asymptomatic carriage. Our sampling was performed ∼6 weeks after the start of the university term, such that most of the carriers were likely to have acquired carriage within that time period (note that carriers V69, V93, and V96 were negative for carriage at time point one) and have developed or were developing an immune response to surface antigens (see Alamro et al. [23] for data on the immune responses and further discussion of acquisition times). Thus, our study is likely to be representative of the adaptive events that occur a few days after initial colonization of carriers by meningococci and, more importantly, when these organisms are subject to adaptive immune responses.

### Examination of multiple isolates distinguishes between sporadic and nonsporadic genetic variation.

Meningococcal epidemiology has focused on measuring genetic variation over periods of months and years. However, this variation arises from processes occurring in individual carriers, which is poorly characterized. Recent studies by Pandey et al. (33) and Borud et al. (34) detected evidence of transient and fixed genetic variants during persistent neisserial carriage but were limited by having only one isolate per time point. Using samples from a 2008–2009 meningococcal carriage study wherein nasopharyngeal swabs were deliberately plated to yield multiple single colonies (see Alamro et al. [23]), we performed both WGS and allele-specific PCR on 6 to 10 isolates/time point from multiple sequential time points from several volunteers. We found that genetic variants can be classified into one of four classes (sporadic, transient, putative fixation, and potential fixation) and that WGS of four isolates/time point differentiates sporadic from transient/fixed variants. Furthermore, transient variants were separable from fixed variants with three time points, while detection of fixation was enhanced by four time points. Thus, we recommend collection of ≥4 isolates for each of ≥4 time points for future longitudinal carriage studies.

### How frequent are mutation and HGT during persistent meningococcal carriage?

While meningococcal mutation rates are low, high rates of turnover of colonizing populations are expected to provide ample time for mutation. Excluding SSR, our WGS analysis indicated an average of 1.02 mutations/genome/month of carriage (i.e., 6.1 × 10^−6^ mutations/site/year). Comparable rates were obtained by Lamelas et al. (35) for serogroup A meningococci (3.1 × 10^−6^ substitutions/site/year), from analysis of 100 isolates representing an 8-year study in a single Ghanaian district, and Pandey et al. (33) for Neisseria lactamica (2.2 × 10^−6^; 27 volunteers) and N. meningitidis (9.3 × 10^−7^; 7 volunteers), using longitudinal isolates from a volunteer study. Meningococcal mutation rates for rifampin resistance are 2.6 × 10^−9^ mutations/division (36), equivalent to 2.2 × 10^−5^ mutations/site/year. The 4-fold differences between these rates suggests that stabilizing selection limits mutation accumulation. However, we observed that nonsynonymous mutations exceeded synonymous mutations. This indicates that stabilizing selection is weak over short periods of persistent carriage and that nonselective bottlenecks, due to expansion after nonspecific physical reductions in population size, could be a key factor in limiting accumulation of variation.

HGT is a major driver of genetic variation in meningococcal populations. We found HGT in 16% of carriers, with 36 recombination events encompassing 51% of all nonhypermutable variable genes. This observation provides definitive evidence from individual infections that natural transformation between two phylogenetically divergent strains generates a high proportion of genetic variation in this bacterial species. This estimate of HGT may be low, as we avoided the 36% of carriers from the 2008–2009 study in which sequential carriage of divergent isolates was detected (45).

### Fixation of genetic variants occurs at similar rates during long- and short-term host persistence.

**FIG 7:**
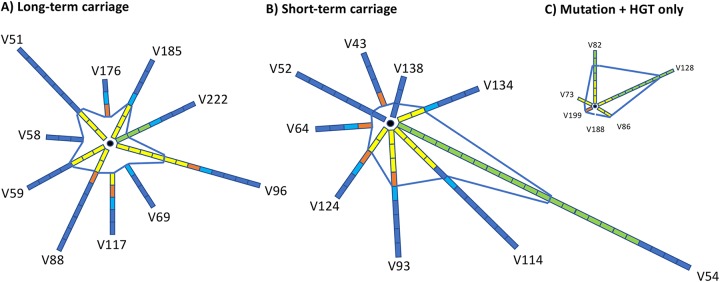
Relative contributions of LH, mutation, and horizontal gene transfer to functional variation during persistent meningococcal carriage. These diagrams depict the relative amounts of different types of genetic variation occurring during natural asymptomatic carriage of meningococci in 23 university students. Each spoke represents the proportion and type of variation relative to a different but homogeneous starting population, indicated by the black circle. We show only genetic variation that alters the amino acid sequence or expression state of genes and has been putatively or probably fixed in the population (note that fixation was determined by comparison of multiple colonies and time points for all PV and the majority of mutation and HGT events). Each rectangle represents either a single gene or a recombination fragment containing one or more variable genes. The rectangles are colored coded to represent different types of recombination events: dark blue, PV; light blue, antigenic variation of the PilE protein; orange, indel; yellow, mutation; green, recombination due to horizontal gene transfer. The blue lines separate events affecting outer membrane proteins or structures on the outside from other functional groups on the inside. (A) Long-term carriage of 5 to 6 months; (B) short-term carriage of 1 to 3 months; (C) carriers where only mutation and HGT has been assessed.

Fixed genetic variation is an indicator of selection. We estimated the extent of fixed functional variation during persistent carriage by focusing on nonsynonymous genic variation and PV. Events classified as putative or potential fixation (see Results for a definition) from this study were combined with those of Alamro et al. on scOMP PV (23). We detected a total of 140 fixed events for the 19 carriers wherein all four modes of genetic variation were examined (71 PVs, 11 antigenic PilE variations, 7 indels, 29 single-nucleotide variants, 22 HGTs). All PV data were derived from analyses of multiple colonies while only 53% (31/58) of the indel/mutation/HGT class and 18% of the PilE variation was from multiple colonies. Figure 7 shows the distribution of genetic events across these 19 carriers plus 6 carriers where only mutation and HGT were examined. Nine carriers exhibited ≤4 events, while the maximum is 23 (4 PVs and 19 HGTs) for V54 (where fixation has not been thoroughly tested) or 12 (5 LHs, 1 indel, and 6 mutations) for V96 and 12 (4 LHs, 3 HGTs) for V222 (where fixation was examined in detail). Overall, we detected an average of 7 genetic events/carrier (note that one recombination event affects multiple genes) with no difference between long- and short-term carriage. These results indicate that meningococcal populations can persist in their hosts with a minimal amount of adaption and that host persistence *per se* is not a strong driver of genetic variability.

### LH is the major driver of genetic variation and most variation affects the outer membrane.

Figure 7 also depicts the relative contributions of the four mechanisms to within-host fixed genetic variation. Seven carriers exhibited variation only by LH or an indel. The average contribution of LH (i.e., PV plus PilE antigenic variation) to fixed genetic variation in each of the 19 carriers was 69% and across all carriers was 59% (82/140; *P* < 0.0001 assuming 2.25% [45] of loci in a genome of 2,000 genes are subject to LH and an equal potential for all genes to undergo directional variation), with again, no significant difference between long- and short-term carriage. These results highlight the critical contribution of LH to within host adaptation.

Another important observation is that 69% of fixed events (96/140; *P* < 0.0001 assuming 8.5% [180] genes in a genome of 2,127 genes affect the outer membrane) affect the outer membrane, with 6 carriers having fixed variation only in outer membrane-associated genes (Fig. 7). While PV was the major driver of this variation, high variability in OMPs was also observed for non-PV indels and HGT, suggesting that the strong diversifying selection acting on these surface antigens requires significant alterations to an epitope as opposed to single amino acid alterations. Comparable hot spots of recombination and variation in capsule biosynthesis, pilin-associated, *pgl*, and OMP-encoding genes have been reported in several comparative population studies of meningococcal isolates (34, 35, 37–39). Major changes in antigenic epitopes during persistent carriage are presumed to facilitate evasion of a polyclonal immune response against surface antigens (40–42).

### Directional PV of the Opa adhesins and PilC proteins.

A key observation was for switching between antigenically divergent Opa alleles during persistent carriage combined with continuous expression of one or more Opa proteins. This result indicates that both maintenance of Opa adhesive functions and antigenic variation are required for persistence on mucosal surfaces. Previously, high turnover of gonococcal Opa variants was observed in experimental human challenge experiments, but without clear evidence of directional switching other than selection against Opa variants with specificity for CEACAM3, the neutrophil decoy receptor (43–45). Our findings suggest that variant-specific antibody responses are frequently elicited during carriage and drive PV-mediated loss of expression of initially dominant Opa variants. Similarly, the PilC proteins modulate meningococcal adhesive properties, with evidence for differential binding of PilC1- and PilC2-expressing variants to different eukaryotic cell types (27). Our finding of frequent switches from PilC1 to PilC2 expression during persistent carriage suggests that the tissue tropism alters during persistent carriage. This process may be driven by immune responses to PilC1 or adaptation to other alterations in the microenvironment. Finally, we observed limited PV of the RM systems, suggesting that selection for alterations in expression of phasevarions, as controlled by PV of RM-mediated methylation (46–48), is infrequent during persistent carriage. These observations are consistent with the idea that PV acts to maintain a range of adhesive functions while enabling frequent antigenic variation and hence a high potential for evasion of antigen-specific antibody responses.

### Conclusion.

We have demonstrated that HGT and LH are the major drivers of within-host adaptive evolution during natural carriage of a bacterial organism. Conversely *de novo* mutations were infrequent and may only contribute significant effects on longer evolutionary timescales. Future studies are required to ascertain the phenotypic effects of observed genetic variation and to correlate these effects with variations in the host niche, including antigen-specific antibody responses. Our study provides a strong baseline for understanding how within-host genetic variation contributes to the evolution of a bacterial commensal pathogen and to the selective pressures driving evolution of HGT and LH.

## MATERIALS AND METHODS

### Bacterial isolates.

Meningococcal isolates were obtained from a carriage study performed at the University of Nottingham between November 2008 and May 2009 as described previously (23, 49). The study was approved by the Nottingham University Medical School Ethics Committee, and written informed consent was obtained from all volunteers.

### Next-generation sequencing.

Genomic DNA was extracted according to the manufacturer’s instructions with either the DNeasy blood and tissue kit (Qiagen) or the Wizard Genomic DNA Purification kit (Promega). Index-tagged Illumina sequencing libraries were generated, multiplexed, and sequenced on Illumina HiSeq 2500 machines to generate paired end sequences. Short sequence reads were trimmed with Trimmomatic v0.32 (50) and assembled with SPAdes v3.9.0 (51).

### Allelic variation and homologous recombination analysis.

The N. meningitidis cgMLST v1.0 scheme within the Genome Comparator tool of the Bacterial Isolate Genome Sequence Database (BIGSdb) platform (52) was used to analyze 1,605 loci for allelic variation. After removal of incomplete loci, phylogenetic networks were derived from allelic variation and visualized using GrapeTree (53). Variable loci were also aligned with Clustal Omega (54) and compared manually. Variability was classified as follows: point mutation, a single nucleotide substitution in a gene; indel, presence or absence of a single or contiguous base pairs within a gene; recombination (either gene conversion or HGT), multiple substitutions (>1) or indels within a gene. Recombination patches were detected by extracting and aligning recombinant allelic sequences for a specific gene of a pair of isolates along with the flanking sequences using tools within BIGSdb. A BLAST analysis was performed with the recombinant allele against subsets of isolates in BIGSdb (e.g., other isolates from the same carrier, all isolates collected in the 2008–2009 carriage study or all UK isolates of the same clonal complex with similar isolation times). The gene and flanking sequences were extracted from the genomes of putative donor isolates with the highest match (usually between 95% and 100% similarity). The contiguous sequences of each pair of recombinant alleles and, where possible, the donor genomes were aligned in order to detect the putative start and end points for intergenic recombination. This process was iterative, with flanking sequences being extracted and aligned until the donor sequence no longer matched the recombinant sequence.

### Enumeration of SSR repeat numbers.

Each SSR was subject to PCR amplification and sequencing using specific primers. Amplicons produced using fluorescently labeled primers were subject to GeneScan fragment size analysis on an ABI3730 autosequencer and compared to either GeneScan 500LIZ or GeneScan 600LIZ size standards (Applied Biosystems). Product sizes were analyzed using PeakScanner v1.0 (Applied Biosystems) and compared to controls of known size. A subset of SSRs were analyzed by dideoxy sequencing and alignment with BioEdit (v7.2.5) to confirm repeat numbers. Two or more labeled products were produced when SSRs contained ≥9 mononucleotide repeats due to slippage during PCR amplification. If the ratio of the areas of the primary and secondary peaks was ≥1.2, then repeat number was determined from the primary peak; if <1.2, then the sample was either reported as mixed or, when occurring across multiple samples, the larger peak was selected as the determinant of repeat number.

### Western blotting.

Meningococcal isolates were grown overnight on brain heart infusion (BHI) agar with 5% horse blood at 37°C with 5% CO_2_. Lysates were prepared from colonies by heating suspensions at 65°C for 15 min in SDS sample loading buffer. Gel electrophoresis of bacterial cell lysates (50 μg) was performed on 10% polyacrylamide gels before transfer to a polyvinylidene difluoride membrane. Glycan-specific monoclonal antibodies (npg1, npg2, and npg3) or a polyclonal antiserum (pDAb2) followed by an alkaline phosphatase-conjugated secondary antibody or an alkaline phosphatase-conjugated succinylated wheat germ agglutinin (sWGA) lectin were used to detect specific protein glycosylation states (28).

### Statistics.

Chi-square tests were performed using an online calculator in GraphPad (https://www.graphpad.com/quickcalcs/).

### Data availability.

All gene alignments and analyses are available at https://leicester.figshare.com/s/24e2782f537d2c77af60. Sequence reads were deposited in the European Nucleotide Archive (ENA), while assembled contigs were deposited into the https://pubmlst.org/neisseria/ database and are accessible through the https://pubmlst.org/ website using the BIGSdb platform (52). The identification (ID) numbers for the isolates in this database are as follows: 28247 to 28298 for the paired isolates from the 25 carriers, 28299 to 28338 for the 39 isolates from carrier V59, and 53889 to 53967 for the multiple isolates from five other carriers.
