# Supplementary material for: Localized Hypermutation is the Major Driver of Meningococcal Genetic Variability during Persistent Asymptomatic Carriage
Source: mBio. 2020 Mar 24;11(2):e03068-19. doi: 10.1128/mBio.03068-19 (PMC7157529; doi:10.1128/mBio.03068-19)
Supplement: FIG S1 [file mBio.03068-19-sf001.pdf]

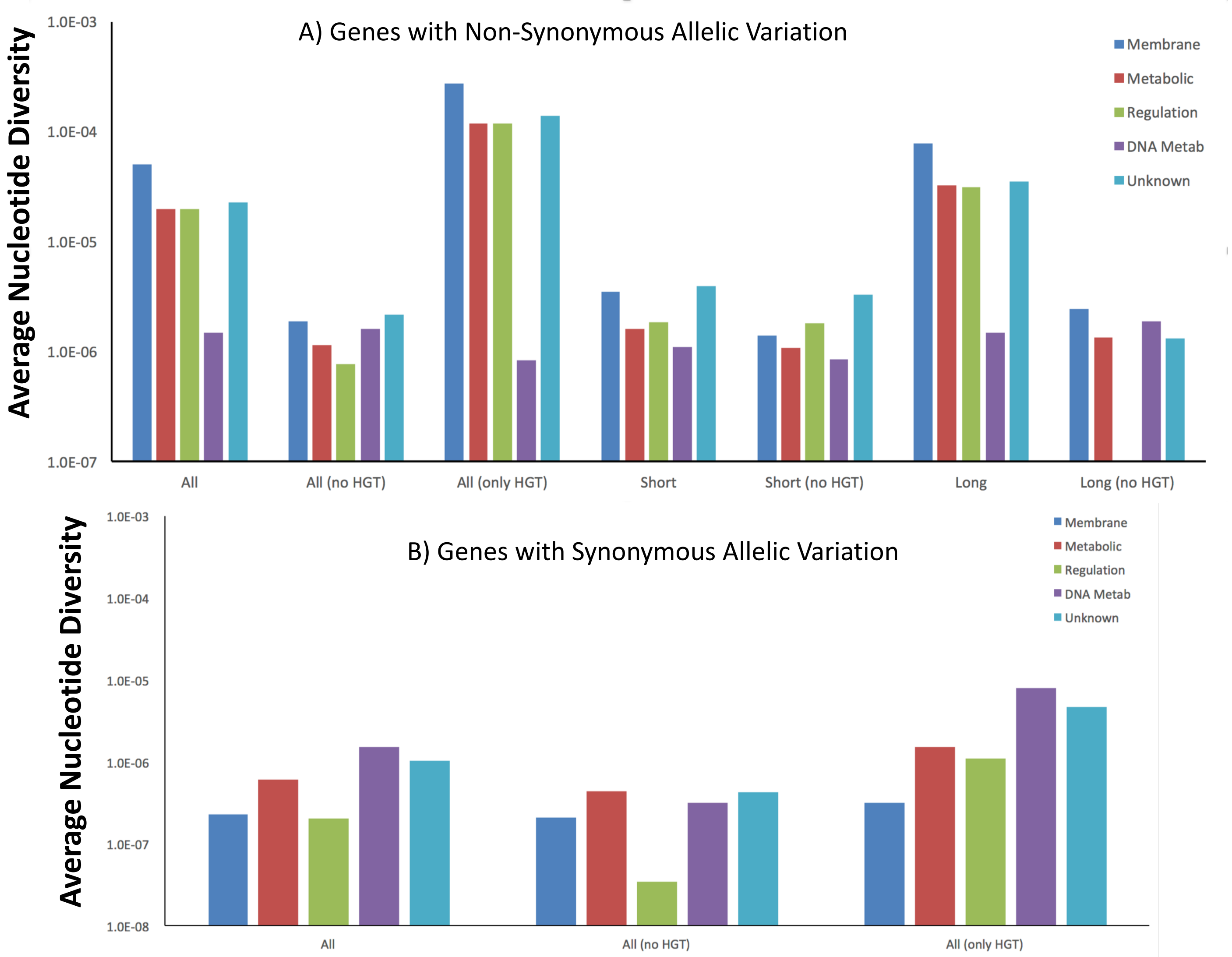

Supplementary Figure 1. Comparison of the nucleotide diversity of genes with non-synonymous and synonymous allelic diversity between paired meningococcal carriage isolates representing 2 to 6 months persistent, asymptomatic carriage in one individual. Variable genes were identified by comparison of genic sequences. Genes were examined for differences in amino acid sequences and separated into those with 1 or more non-synonymous differences (A) or only synonymous differences (B). Genes were then separated into one of four functional gene classes. Nucleotide diversity was calculated by dividing the numbers of differences in a gene by the total gene length and an average calculated for all variable genes in that class. This average number was then adjusted to account for the number of genes in each functional class and number of carriers analysed. The average nucleotide diversity is presented for various classifications:- All, all 25 pairs of isolates; All (no HGT), 21 carriers where no horizontal gene transfer was observed; All (only HGT), the four carriers were HGT was observed; Short, 10 carriers representing 2-3 months persistent carriage; Long, 15 carriers representing 5-6 months persistent carriage.
