# Supplementary material for: Localized Hypermutation is the Major Driver of Meningococcal Genetic Variability during Persistent Asymptomatic Carriage
Source: mBio. 2020 Mar 24;11(2):e03068-19. doi: 10.1128/mBio.03068-19 (PMC7157529; doi:10.1128/mBio.03068-19)
Supplement: TABLE S1 [file mBio.03068-19-st001.docx]

Table S1. Allelic variation in PilE amino acid sequences for longitudinal carriage isolates

| Months of Carriage | Volunteer (isolates) | % identity | Average % identity |
| --- | --- | --- | --- |
| 1-2 | V122 (N122.1/N462.1)^1^ | 88 | 88 |
|  | V223 (N123.1/N393.1) ^1^ | 91 |  |
| 3-4 | V64 (N64.1/N348.1)^2^ | 83 | 86 |
|  | V93 (N264.1/N359.1) ^2^ | 87 |  |
|  | V114 (N114.1/N330.1) ^1^ | 88 |  |
|  | V124 (N124.1/N336.1) ^1^ | 86 |  |
|  | V134 (N134.1/N333.1) ^1^ | 81 |  |
|  | V140 (N140.1/N335.1) ^1^ | 83 |  |
|  | V199 (N199.1/N378.1) ^1^ | 85 |  |
|  | V209 (N383.1/N460.1) ^1^ | 91 |  |
| 5-6 | V82 (N262.1/N446.1) ^1^ | 87 | 88 |
|  | V86 (N86.1/N447.1) ^1^ | 96 |  |
|  | V96 (N259.1/N445.1) ^2^ | 81 |  |
|  | V117 (N117.1/N417.3) ^1^ | 98 |  |
|  | V128 (N128.1/N420.1) ^2^ | 88 |  |
|  | V131 (N131.1/N422.1) ^1^ | 81 |  |
|  | V176 (N176.1/N408.1) ^1^ | 94 |  |
|  | V188 (N188.1/N462.1) ^1^ | 81 |  |
|  | V222 (N222.1/N459.1) ^1^ | 88 |  |

^1^PilE sequences generated by PCR amplification and Sanger sequencing of PCR products; ^2^PilE sequences generated from NGS of genomic DNA
