# Supplementary material for: Localized Hypermutation is the Major Driver of Meningococcal Genetic Variability during Persistent Asymptomatic Carriage
Source: mBio. 2020 Mar 24;11(2):e03068-19. doi: 10.1128/mBio.03068-19 (PMC7157529; doi:10.1128/mBio.03068-19)
Supplement: FIG S3 [file mBio.03068-19-sf003.pdf]

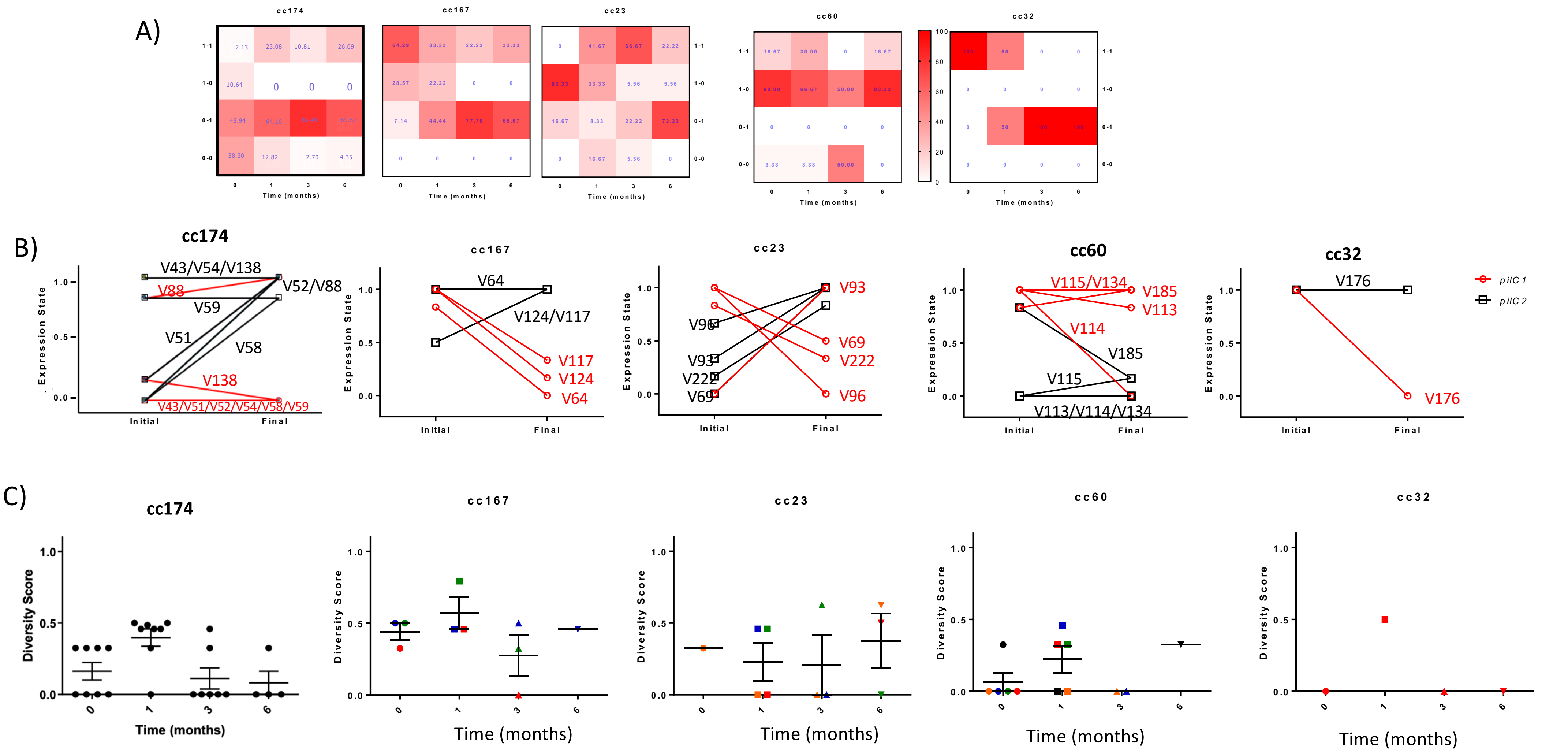

Supplementary Figure 3. Phasotypes for the pilin modulation module. A) Proportion of isolates containing different combinations of expression of the *pilC1* and *pilC2* phase-variable genes (1 and 0 indicate ON and OFF states, respectively). Percentage is calculated from total number of isolates analysed at each time point; cc174, 47, 39, 37, 23; cc167, 14, 18, 18, 6; cc23, 6, 24, 18, 18; cc60, 30, 30, 12, 6; cc32, 6, 6, 6, 6. B) Change in expression for *pilC1* and *pilC2* in each carrier between the initial and final time point. Values of 1 and 0, all observed isolates in ON or OFF state, respectively. C) Shannon diversity scores. Diversity scores were calculated from the combined expression score of the *pilC1* and *pilC2* genes for multiple isolates per time point; 1, all isolates were different from each other; 0, all isolates had same score.
