# Supplementary material for: Localized Hypermutation is the Major Driver of Meningococcal Genetic Variability during Persistent Asymptomatic Carriage
Source: mBio. 2020 Mar 24;11(2):e03068-19. doi: 10.1128/mBio.03068-19 (PMC7157529; doi:10.1128/mBio.03068-19)

Supplementary Figure 4. Western blot to determine the expression states of a sub-set of persistent carriage isolates. Whole cell lysates were prepared from 15 meningococcal isolates obtained as part of this study. Lysates were subject to separation on a PAGE gel and then transferred to a nitrocellulose membrane. These membranes were probed with monoclonal antibodies specific for a range of different glycan extensions.

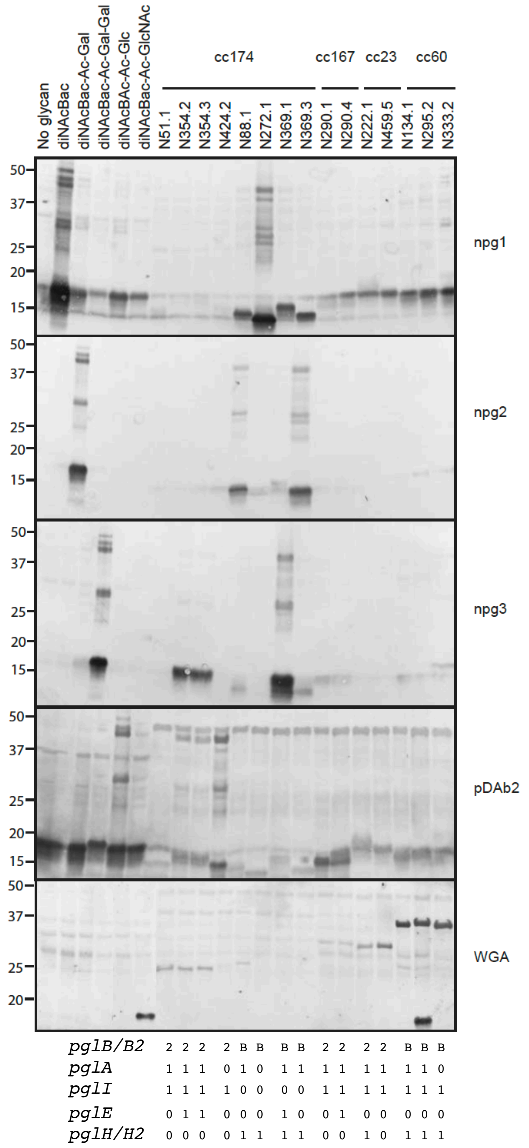

Supplement: FIG S4 [file mBio.03068-19-sf004.pdf]
