# Supplementary material for: Localized Hypermutation is the Major Driver of Meningococcal Genetic Variability during Persistent Asymptomatic Carriage
Source: mBio. 2020 Mar 24;11(2):e03068-19. doi: 10.1128/mBio.03068-19 (PMC7157529; doi:10.1128/mBio.03068-19)
Supplement: FIG S5 [file mBio.03068-19-sf005.pdf]

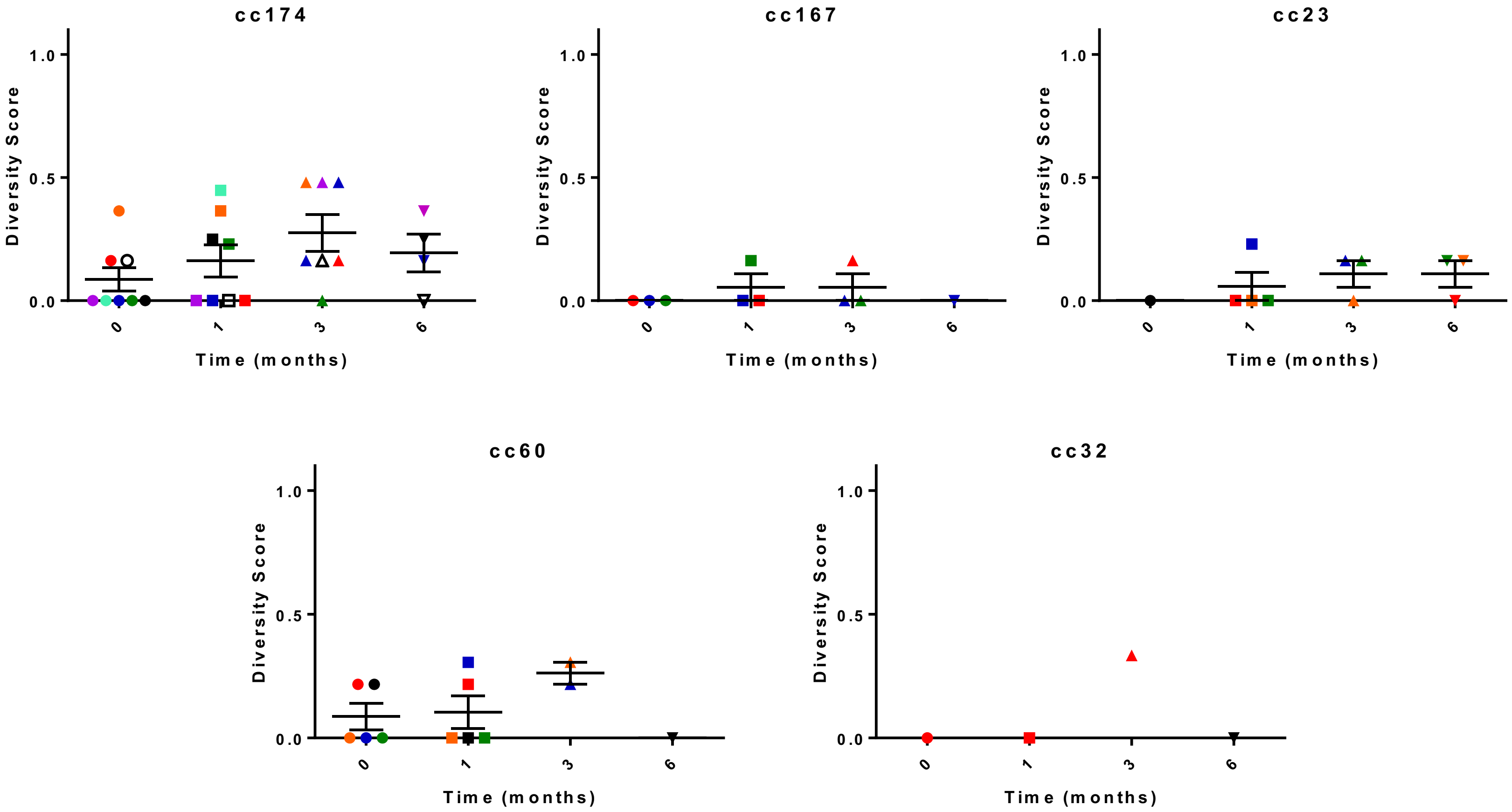

Supplementary Figure 5. Shannon diversity scores for each time point for the pilin glycosylation module. The genes in this module and method for calculation of expression score is indicated in Figure 5. The diversity was calculated as described in Supplementary Figure 3.
