# Supplementary material for: Localized Hypermutation is the Major Driver of Meningococcal Genetic Variability during Persistent Asymptomatic Carriage
Source: mBio. 2020 Mar 24;11(2):e03068-19. doi: 10.1128/mBio.03068-19 (PMC7157529; doi:10.1128/mBio.03068-19)
Supplement: FIG S6 [file mBio.03068-19-sf006.pdf]

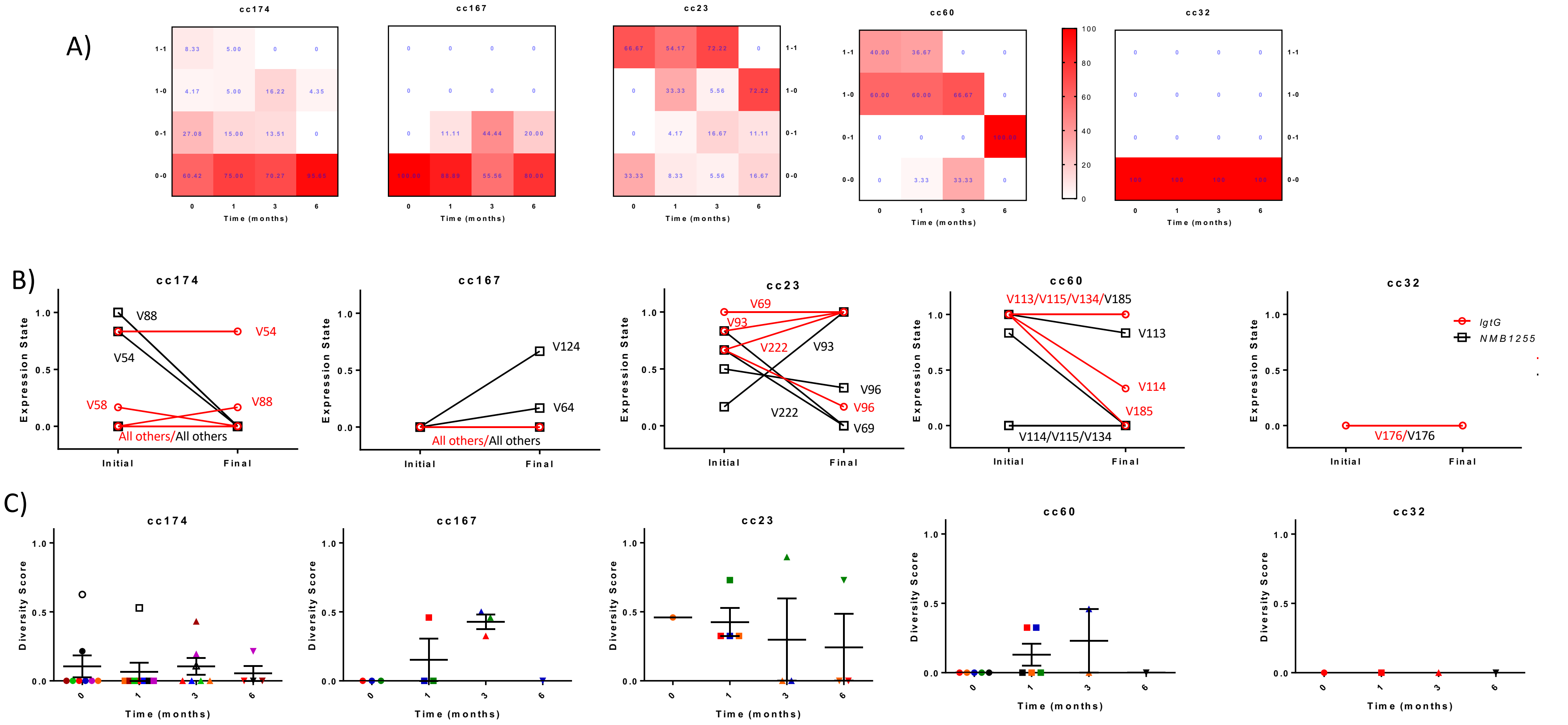

Supplementary Figure 6. Phasotypes for the LPS module (*lgtG* and *NMB1255*). A) Percentages were calculated as described in Supplementary Figure 3 for the following numbers of isolates; cc174, 48, 40, 37, 23; cc167, 14, 18, 18, 15; cc23, 6, 24, 18, 18; cc60, 30, 30, 12, 6; cc32, 6, 6, 6, 6. B) Switching states for each volunteer for the LPS module. See Supplementary Figure 3 for method for determination of expression states. C) Shannon diversity scores for each time point. See Supplementary Figure 3 for how diversity scores were derived.
