# Supplementary material for: Localized Hypermutation is the Major Driver of Meningococcal Genetic Variability during Persistent Asymptomatic Carriage
Source: mBio. 2020 Mar 24;11(2):e03068-19. doi: 10.1128/mBio.03068-19 (PMC7157529; doi:10.1128/mBio.03068-19)
Supplement: FIG S7 [file mBio.03068-19-sf007.pdf]

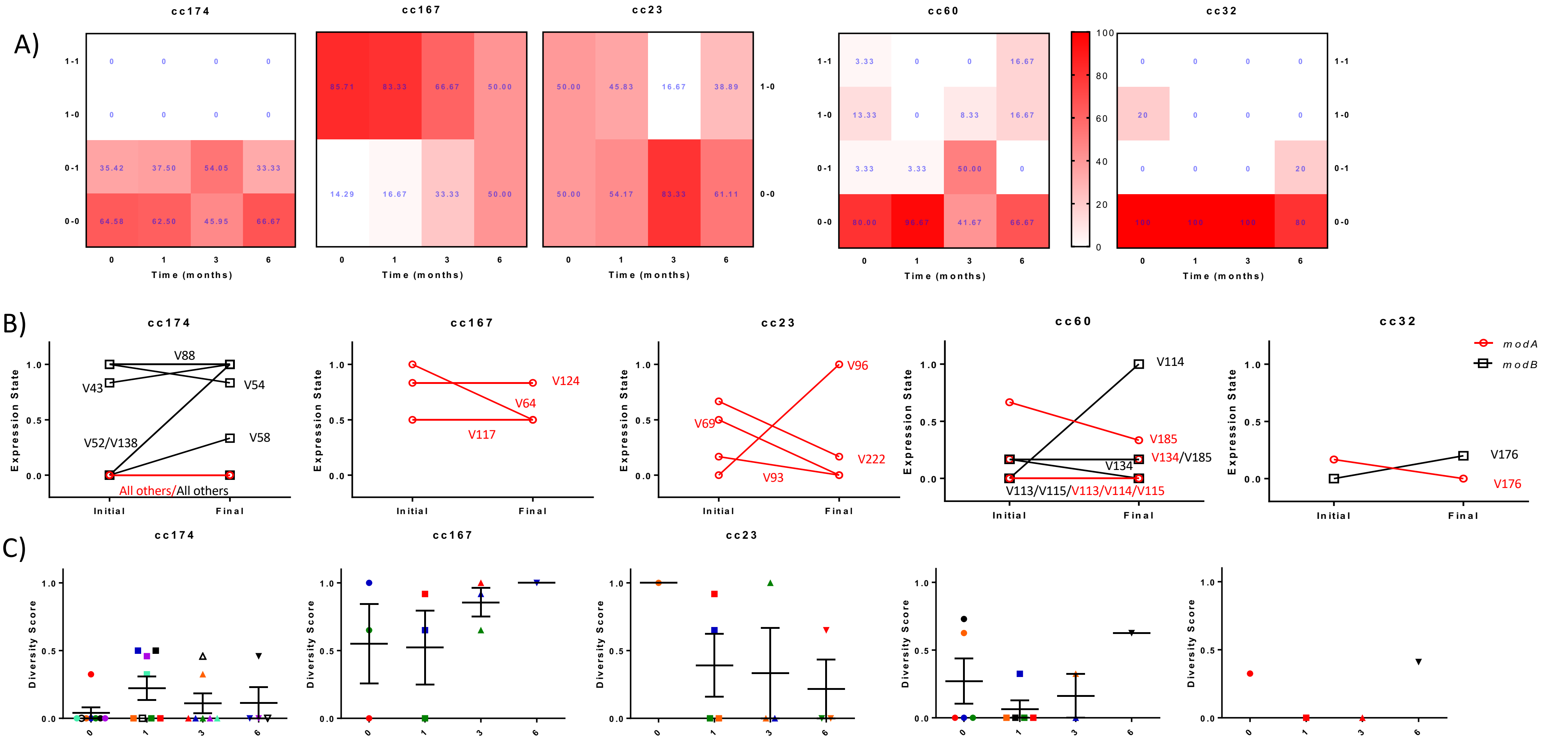

Supplementary Figure 7. Phasotypes for the Mod module (*modA* and *modB)*. A) Percentages were calculated as described in Supplementary Figure 3 for the following numbers of isolates; cc174, 48, 40, 37, 24; cc167, 14, 18, 18, 6; cc23, 6, 24, 18, 18; cc60, 30, 30, 12, 6; cc32, 6, 6, 6, 5. B) Switching states for each volunteer for the Mod module. See Supplementary Figure 3 for how expression states were determined. Note that the cc167 and cc23 strains did not contain the *modB* gene. C) Shannon diversity scores. See Supplementary Figure 3 for how diversity scores were derived
