# Supplementary figures and images for: Localized Hypermutation is the Major Driver of Meningococcal Genetic Variability during Persistent Asymptomatic Carriage
Source: mBio. 2020 Mar 24;11(2):e03068-19. doi: 10.1128/mBio.03068-19 (PMC7157529; doi:10.1128/mBio.03068-19)

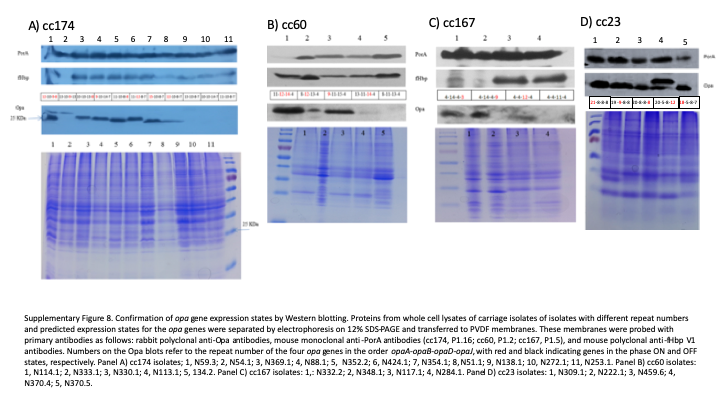

Supplement: FIG S8 [file mBio.03068-19-sf008.tif]
