# Supplementary material for: Localized Hypermutation is the Major Driver of Meningococcal Genetic Variability during Persistent Asymptomatic Carriage
Source: mBio. 2020 Mar 24;11(2):e03068-19. doi: 10.1128/mBio.03068-19 (PMC7157529; doi:10.1128/mBio.03068-19)
Supplement: FIG S2 [file mBio.03068-19-sf002.pdf]

```
N332-2_1      MNTLQKGFTLIELMIXIAIVGILAAVALPAYQDYTARAQVSEAILLAEGQKSAVTEYYLN   60
N284-3_1      MNTLQKGFTLIELMIVIAIVGILAAVALPAYQDYTARAQVSEAILLAEGQKSAVTEYYLN   60
N284-6_1      MNTLQKGFTLIELMIVIAIVGILAAVALPAYQDYTARAQVSEAILLAEGQKSAVTEYYLN   60
N332-3_1      MNTLQKGFTLIELMIVIAIVGILAAVALPAYQDYTARAQVSEAILLAEGQKSAVTEYYLN   60
N332-1_1      MNTLQKGFTLIELMIVIAIVGILAAVALPAYQDYTARAQVSEAILLAEGQKSAVTEYYLN   60
N332_4_1      MNTLQKGFTLIELMIVIAIVGILAAVALPAYQDYTARAQVSEAILLAEGQKSAVTEYYLN   60
N332-6_1      MNTLQKGFTLIELMIVIAIVGILAAVALPAYQDYTARAQVSEAILLAEGQKSAVTEYYLN   60
N284-1_1      MNTLQKGFTLIELMIVIAIVGILAAVALPAYQDYTARAQVSEAILLAEGQKSAVTEYYLN   60
N285-5_1      MNTLQKGFTLIELMIVIAIVGILAAVALPAYQDYTARAQVSEAILLAEGQKSAVTEYYLN   60
N284-2_1      MNTLQKGFTLIELMIVIAIVGILAAVALPAYQDYTARAQVSEAILLAEGQKSAVTEYYLN   60
N284-4_1      ------------------------AVALPAYQDYTARAQVSEAILLAEGQKSAVTEYYLN   36
N417-6_1      MNTLQKGFTLIELMIVIAIVGILAAVALPAYQDYTARAQVSEAILLAEGQKSAVTEYYLN   60
N117-1_1      MNTLQKGFTLIELMIVIAIVGILAAVALPAYQDYTARAQVSEAILLAEGQKSAVTEYYLN   60
N417-1_1      MNTLQKGFTLIELMIVIAIVGILAAVALPAYQDYTARAQVSEAILLAEGQKSAVTEYYLN   60
N417_2_1      MNTLQKGFTLIELMIVIAIVGILAAVALPAYQDYTARAQVSEAILLAEGQKSAVTEYYLN   60
N417-3_1      MNTLQKGFTLIELMIVIAIVGILAAVALPAYQDYTARAQVSEAILLAEGQKSAVTEYYLN   60
N417-4_1      MNTLQKGFTLIELMIVIAIVGILAAVALPAYQDYTARAQVSEAILLAEGQKSAVTEYYLN   60
N417-5_1      MNTLQKGFTLIELMIVIAIVGILAAVALPAYQDYTARAQVSEAILLAEGQKSAVTEYYLN   60
              ************************************************************

N332-2_1      HGEWPDGNSNAGVASSSTIKGKYVKEVTVANGVITATMLSSGVNKEIQGKKLSLWAKRQD   120
N284-3_1      HGEWPGNNSSAGVASSSTIKGKYVEKVEVKNGVVTATMLSSGVNKEIQGKKLSLWAKRQD   120
N284-6_1      HGEWPGNNSSAGVATSANIKGKYVEKVEVKNGVVTATMLSTGVNKEIKGKKLSLWAKRQD   120
N332-3_1      HGEWPDGNSNAGVASSSTIKGKYVKEVTVANGVITATMLSSGVNKEIQGKKLSLWAKRQD   120
N332-1_1      HGEWPGNNSSAGVASSSTIKGKYVKEVTVAKGVITATMLSSGVNKEIQGKKLSLWAKRQD   120
N332_4_1      HGEWPGNNSSAGVASSSTIKGKYVKEVTVANGVITATMLSSGVNKEIQGKKLSLWAKRQD   120
N332-6_1      HGIWPGNNSSAGVASSSTIKGKYVKEVTVANGVITATMLSSGVNKEIQGKKLSLWAKRQD   120
N284-1_1      HGIWPGNNSSAGVASSSTIKGKYVKEVTVANGVITATMLSSGVNKEIQGKKLSLWAKRQD   120
N285-5_1      HGIWPGNNSSAGVASSSTIKGKYVKEVTVANGVITATMLSSGVNKEIQGKKLSLWAKRQD   120
N284-2_1      HGIWPGNNSSAGVASSSTIKGKYVKEVTVANGVITATMLSSGVNKEIQGKKLSLWAKRQD   120
N284-4_1      HGIWPGNNSSAGVASSSTIKGKYVKEVTVANGVITATMLSSGVNKEIQGKKLSLWAKRQD   96
N417-6_1      HGEWPGNNSSAGVASSSTIKGKYVEKVEVANGVITATMLSSGVNNEIKGKKLSLWAKRQD   120
N117-1_1      HGEWPGNNSSAGVASSSTIKGKYVKEVTVANGVITATMLSSGVNNEIKGKKLSLWAKRQD   120
N417-1_1      HGEWPGNNSSAGVASSSTIKGKYVKEVTVANGVITATMLSSGVNNEIKGKKLSLWAKRQD   120
N417_2_1      HGEWPGNNSSAGVASSSTIKGKYVKEVTVANGVITATMLSSGVNNEIKGKKLSLWAKRQD   120
N417-3_1      HGEWPGNNSSAGVASSSTIKGKYVEKVEVANGVITATMLSSGVNNEIKGKKLSLWAKRQD   120
N417-4_1      HGEWPGNNSSAGVASSSTIKGKYVEKVEVANGVITATMLSSGVNNEIKGKKLSLWAKRQD   120
N417-5_1      HGEWPGNNSSAGVASSSTIKGKYVEKVEVANGVITATMLSSGVNNEIKGKKLSLWAKRQD   120
              ** **..**.****:*:.******::* * :**:******:***:**:************

N332-2_1      GSVKWFCGLPVTRTDAKADTVAA-----DSDKQIDTKHLPSTCRDASSAG*  165
N284-3_1      GSVKWFCGLPVTRTDAKADDVTA-----DSDKQIDTKHLPSTCRDASSAG*  165
N284-6_1      GSVKWFCGQPVQRANAKADDVTA-----DSDKQIDTKHLPSTCRDASSAG*  165
N332-3_1      GSVKWFCGQPVQRANAKADDVAAAAANGKTADNINTLHLPSTCRDAASAG*  170
N332-1_1      GSVKWFCGQPVQRANAKADDVAAAAANGKTADNINTKHLPSTCRDAASAG*  170
N332_4_1      GSVKWFCGQPVQRANAKADDVAAAAANGKTADNINTKHLPSTCRDAASAG*  170
N332-6_1      GSVKWFCGQPVKRD-AKADTVAAAA---KTADNINTKHLPSTCRDASSAG*  166
N284-1_1      GSVKWFCGLPVTRTDAKADTVAAAA---KTADNINTKHLPSTCRDASSAG*  167
N285-5_1      GSVKWFCGLPVTRTDAKADTVAAAA---KTADNINTKHLPSTCRDASSAG*  167
N284-2_1      GSVKWFCGLPVTRTDAKADTVAAAAAA-KTADNINTKHLPSTCRDASSAG*  169
N284-4_1      GSVKWFCGLPVTRTDAKADTVAAAAAA-KTADNINTKHLPSTCRDASSAG*  145
N417-6_1      GSVKWFCGQPVTRDAK-ADTVAA-----KTADNINTKHLPSTC-------   165
N117-1_1      GSVKWFCGQPVTRDAK-ADTVAA-----KTADNINTKHLPSTCRDAASAG*  164
N417-1_1      GSVKWFCGQPVTRDAK-ADTVAA-----KTADNINTKHLPSTCRDAASAG*  164
N417_2_1      GSVKWFCGQPVTRDAK-ADTVAA-----KTADNINTKHLPSTCRDAASAG*  164
N417-3_1      GSVKWFCGQPVTRDAK-ADTVAA-----KTADNINTKHLPSTCRDAASAG*  164
N417-4_1      GSVKWFCGQPVTRDAK-ADTVAA-----KTADNINTKHLPSTCRDAASAG*  164
N417-5_1      GSVKWFCGQPVTRDAK-ADTVAA-----KTADNINTKHLPSTCRDAASAG*  164
              ******** ** *    ** * *     .: .:*:* *********  **
```

Supplementary Figure 2. Allelic variation in the derived PilE amino acid sequences of multiple meningococcal isolates from a single carrier. The *pilE* gene was amplified from 19 meningococcal carriage isolates obtained from volunteer V117. The isolates were obtained at the first (2, N117), second (6, N284), third (5, N332) and fourth (6, N417) time points and represent initial, one, three and six months persistent carriage. Gene sequences were translated and aligned using Clustal Omega.
