# Supplementary material for: Localized Hypermutation is the Major Driver of Meningococcal Genetic Variability during Persistent Asymptomatic Carriage
Source: mBio. 2020 Mar 24;11(2):e03068-19. doi: 10.1128/mBio.03068-19 (PMC7157529; doi:10.1128/mBio.03068-19)
Supplement: DATA FILE S1 [file mBio.03068-19-sd001.pdf]

This file contains five tables (I-V) listing the phase variation and Gene Scan data for all isolates for pilin modulation, pilin glycosylation, restriction-modification and Opa modules of phase-variable genes. The following information is listed for every gene for each isolate; size of PCR product as determined by GeneScan; derived repeat number (as determined from GeneScan relative to a product of known repeat number); derived expression state (as determined from a bioinformatic analysis of the open reading frame or relative to other isolates of known expression state). Two additional items of information are provided for the Opa genes; size of G tract and size of A tract (these two tracts are adjacent to the repetitive sequence and their length influences the expression state)

Supplementary Data Table I. Repeat numbers and expression states for pilin modulation and LOS phase-variable genes

Volunteer /time pt	Isolate	Phase Variable Gene											
		lgtG			*NMB1255*			*pilC1*[4]			*pilC2*		
		Size[1]	Tract[2]	Exp. State[3]	Size[1]	Tract[2]	Exp. State[3]	Size[1]	Tract[2]	Exp. State[3]	Size[1]	Tract[2]	Exp. State[3]
**cc174**													
V43/1st	N43.1.1	372.1	12C*	0	377.0	10G	1	256.5	14G	0	353.7	13G	1
	N43.1.2	372.0	12C	0	377.2	10G	1	256.4>255.5 {1.2}	14G	0	353.5	13G	1
	N43.1.3	372.2	12C	0	377.0	10G	1	256.4	14G	0	353.6	13G	1
	N43.1.4	372.0	12C	0	376.8	10G	1	256.3	14G	0	353.3	13G	1
	N43.1.5	371.9	12C	0	376.8	10G	1	256.3	14G	0	353.4	13G	1
	N43.1.6	372.1	12C	0	377.1	10G	1	256.3	14G	0	353.3	13G	1
V43/2nd	N241.1	369.9	10C	0	376.0	9G	0	255.9 (380.4)	10G* (13G)	0	355.1	13G*	1
	N241.2	370.1	10C	0	375.9	9G	0	255.9 (381.4)	10G (14G)	1	355.2	13G	1
V43/3rd	N349.1	369.9	10C	0	375.9	9G	0	255.9 (380.4)	10G* (13G)	0	355.2	13G*	1
	N349.2	370.2	10C	0	375.9	9G*	0	255.9 (380.5)	10G (13G)	0	355.1	13G	1
	N349.3	370.0	10C	0	375.7	9G	0	256.0 (380.4)	10G (13G)	0	355.1	13G	1
	N349.4	370.1	10C	0	376.0	9G	0	256.1 (379.5)	10G (12G)	0	355.1	13G	1
	N349.5	370.3	10C	0	376.0	9G	0	255.9 (380.4)	10G (13G)	0	355.1	13G	1
	N349.6	370.2	10C	0	376.0	9G	0	256.2 (380.4)	10G (13G)	0	355.1	13G	1
V43/4th	Cleared												
V51/1st	N51.1	370.0	10C	0	375.9	9G	0	256.1 (380.5)	10G* (13G)	0	356.0	14G*	0
	N51.2	370.1	10C	0	376.0	9G	0	256.3 (380.5)	10G (13G)	0	354.9	13G	1
	N51.3	369.8	10C	0	376.1	9G	0	256.3 (380.8)	10G (13G*)	0	355.9	14G	0

	N51.4	370.1	10C	0	376.2	9G	0	256.3 (380.6)	10G (13G)	0	355.9	14G	0
	N51.5	369.9	10C	0	376.0	9G	0	256.3 (380.5)	10G (13G*)	0	355.9	14G	0
	N51.6	370.2	10C	0	376.0	9G	0	256.3 (380.5)	10G (13G)	0	355.8	14G	0
V51/2nd	N236.1	369.9	10C	0	375.9	9G	0	256.5 (380.6)	10G* (13G)	0	354.8	13G*	1
	N236.2	370.2	10C	0	376.0	9G	0	255.9 (380.6)	10G (13G)	0	355.0	13G	1
	N236.3	369.9	10C	0	375.8	9G	0	256.3 (380.5)	10G* (13G)	0	355.0	13G	1
	N236.4	370.2	10C	0	376.0	9G	0	256.3 (380.6)	10G (13G)	0	356.0	14G	0
	N236.5	370.2	10C	0	376.1	9G	0	256.4 (380.5)	10G (13G)	0	355.9	14G	0
	N236.6	370.2	10C	0	376.4	9G	0	256.4 (380.5)	10G (13G)	0	355.0	13G	1
V51/3rd	N354.1	370.1	10C	0	376.1	9G	0	256.4 (380.5)	10G (13G)	0	355.8	14G	0
	N354.2	370.2	10C	0	375.6*	9G	0	256.0 (380.3)	10G (13G)	0	355.2	13G	1
	N354.3	370.2	10C	0	375.9	9G	0	255.9 (380.4)	10G (13G*)	0	354.9	13G	1
	N354.4	370.1	10C	0	376.3	9G	0	256.4 (380.6)	10G (13G)	0	354.9	13G	1
	N354.5	370.0	10C	0	376.1	9G	0	256.0 (380.5)	10G (13G)	0	355.2	13G	1
	N354.6	370.1	10C	0	376.3	9G	0	256.4 (380.5)	10G (13G)	0	355.0	13G	1
V51/4th	N424.1	370.0	10C	0	375.9	9G	0	252.7 (379.6)	10G (12G)	0	353.4	13G	1
	N424.2	370.2	10C	0	376.1	9G	0	255.9 (379.6)	10G (12G)	0	355.2 (13)	13G	1
	N424.3	370.1	10C	0	375.9	9G	0	256.4 (379.6)	10G (12G)	0	354.9 (13)	13G	1
	N424.4	370.1	10C	0	375.9	9G	0	256.1 (379.6)	10G (12G)	0	355.1 (13)	13G	1

	N424.5	370.1	10C	0	375.8	9G	0	256.5 (379.6)	10G* (12G)	0	354.9 (13)	13G	1
	N424.6	370.1	10C	0	376.1	9G	0	256.1 (379.6)	10G (12G)	0	355.1 (13)	13G	1
V52/1st	N52.1	370.6	10C*	0	376.0	9G	0	256.2 (380.6)	10G (13G)	0	356.0	14G*	0
	N52.2	370.1	10C	0	376.1	9G	0	256.0 (380.6)	10G (13G)	0	356.0	14G	0
	N52.3	370.4	10C	0	376.1	9G	0	255.9 (380.6)	10G (13G)	0	356.0	14G	0
	N52.4	370.1	10C	0	376.3	9G	0	256.0 (380.8)	10G (13G)	0	356.1	14G	0
	N52.5	370.2	10C	0	376.1	9G	0	255.9 (380.7)	10G (13G)	0	356.1	14G	0
	N52.6	370.1	10C	0	376.2	9G	0	256.0 (380.7)	10G (13G)	0	356.1	14G	0
V52/2nd	N238.1	370.0	10C	0	375.9	9G	0	256.0 (380.4)	10G (13G)	0	355.2	13G*	1
	N238.2	370.6	10C	0	376.0	9G	0	255.9 (380.4)	10G* (13G)	0	355.2	13G	1
	N238.3	370.0	10C	0	375.8	9G	0	255.9 (380.4)	10G (13G)	0	356.2	14G	0
	N238.4	370.1	10C	0	376.2	9G	0	255.9 (380.5)	10G (13G)	0	355.2	13G	1
	N238.5	370.2	10C	0	376.0	9G	0	252.8 (380.4)	10G (13G)	0	353.3	13G	1
	N238.6	369.7	10C	0	376.2	9G	0	255.9 (380.6)	10G (13G)	0	356.1	14G	0
V52/3rd	N342.1	370.2	10C	0	375.9	9G	0	256.1 (380.6)	10G (13G*)	0	355.1	13G	1
V52/4th	Cleared												
V54/1st	N54.1.1	371.1	11C	1	378.2	11G	0	257.8 (374.5)	12G (11G)	0	352.3	10G	1
	N54.1.2	370.1	10C	0	377.1	10G	1	257.8>256.9 {1.1} (375.0)	12G (12G)	0	352.3	10G	1
	N54.1.3	371.0	11C	1	377.2	10G	1	256.8>257.7	11G	0	352.3	10G	1

								{1.2} (375.5)	(12G)			
	N54.1.4	371.1	11C	1	377.0	10G	1	257.8>256.9 {1} (375.6)	12G (12G)	0	352.3	10G	1
	N54.1.5	371.0	11C	1	377.2	10G	1	257.8 (375.6)	12G (12G)	0	352.2	10G	1
	N54.1.6	370.9	11C	1	377.1	10G	1	254.4>253.5 {1} (375.5)	12G (12G)	0	350.4	10G*	1
V54/2nd	N237.1	370.1	10C	0	378.2	11G*	0	254.3 (374.5)	12G (11G)	0	350.3	10G	1
	N237.2	371.0	11C*	1	378.0	11G	0	255.6 (374.6)	13G (11G)	1	350.4	10G	1
	N237.3	370.0	10C	0	378.1	11G	0	257.8 (374.5)	12G (11G)	0	352.3	10G	1
	N237.4	371.0	11C	1	378.0	11G	0	258.8 (374.6)	13G (11G)	1	352.3	10G	1
	N237.5	371.0	11C	1	377.1	10G	1	254.3 (374.5)	12G (11G)	0	350.3	10G	1
	N237.6	371.1	11C	1	377.0	10G*	1	254.3 (373.6)	12G (10G)	0	350.4	10G	1
V54/3rd	N343.1	371.3	11C	1	379.1	12G	0	258.8 (375.5)	13G (12G)	1	352.2	10G	1
	N343.2	371.1	11C	1	378.1	11G	0	257.7 (375.6)	12G (12G)	0	352.3	10G	1
	N343.3	371.1	11C	1	378.3	11G	0	257.6 (375.5)	12G (12G)	0	352.4	10G	1
	N343.4	371.0	11C	1	379.2	12G*	0	255.5 (375.4)	13G (12G)	0	350.3	10G	1
	N343.5	370.1	10C	0	379.2	12G	0	255.4 (375.5)	13G (12G)	0	350.4	10G	1
	N343.6	371.0	11C	1	379.3	12G	0	255.4 (375.3)	13G (12G*)	0	350.1	10G	1
V54/4th	Replaced											
V58/1st	N58.1	370.1	10C	0	376.2	9G	0	256.0 (380.7)	10G* (13G)	0	355.9	14G*	0
	N58.2	368.0	8C*	1	376.0	9G	0	256.1 (380.7)	10G (13G)	0	356.1	14G	0
	N58.3	370.0	10C	0	376.0	9G	0	256.0	10G	0	356.0	14G	0

								(380.6)	(13G)				
	N58.4	370.1	10C	0	376.1	9G	0	255.9<br>(379.6)	10G<br>(12G)	0	356.0	14G	0
	N58.5	370.3	10C	0	376.0	9G	0	256.0<br>(380.7)	10G<br>(13G)	0	356.0	14G	0
	N58.6	370.2	10C	0	376.3	9G	0	255.9<br>(ND)	10G<br>(13G)	ND	355.1>354.2<br>{1}	13G	1
V58/2nd	N240.1	370.1	10C	0	375.9	9G	0	252.5<br>(380.4)	10G<br>(13G)	0	354.4	14G	0
	N240.2	370.2	10C	0	375.8	9G	0	252.5<br>(380.4)	10G<br>(13G)	0	353.1	13G	1
V58/3rd	Cleared												
V58/4th	N429.1	370.2	10C	0	376.0	9G	0	256.0<br>(379.6)	10G*<br>(12G)	0	355.1	13G*	1
	N429.2	370.1	10C	0	376.1	9G	0	256.0<br>(379.6)	10G<br>(12G)	0	355.1	13G	1
	N429.3	370.1	10C	0	375.6	9G	0	256.0<br>(380.5)	10G<br>(13G)	0	355.1	13G	1
	N429.4	370.1	10C	0	375.7	9G	0	256.1<br>(380.3)	10G<br>(13G)	0	355.0	13G	1
	N429.5	370.2	10C	0	376.1	9G	0	256.0<br>(379.6)	10G<br>(12G)	0	353.4	13G*	1
	N429.6	370.1	10C	0	376.0	9G	0	256.1<br>(380.5)	10G<br>(13G)	0	355.1	13G	1
V59/1st	N59.1.1	370.1	10C	0	376.1	9G	0	256.4<br>(380.4)	10G*<br>(13G)	0	355.0	13G*	1
	N59.1.2	370.1	10C	0	375.9	9G	0	255.8<br>(379.4)	10G<br>(12G)	0	355.0	13G	1
	N59.1.3	370.2	10C	0	376.2	9G	0	256.3<br>(380.8)	10G<br>(13G)	0	355.9	14G	0
	N59.1.4	370.1	10C	0	375.4	8G	0	256.3<br>(379.6)	10G<br>(12G)	0	354.9	13G	1
	N59.1.5	370.0	10C	0	376.1	9G	0	256.0<br>(379.6)	10G<br>(12G)	0	355.2	13G	1
	N59.1.6	370.3	10C	0	376.0	9G	0	256.6<br>(379.7)	10G*<br>(12G)	0	354.9	13G	1
V59/2nd	N253.1	370.0	10C	0	376.0	9G	0	256.2	10G*	0	355.0	13G*	1

								(379.4)	(12G)				
	N253.2	370.2	10C	0	376.1	9G	0	256.3 (379.4)	10G (12G)	0	355.0	13G	1
	N253.3	370.1	10C	0	375.9	9G	0	256.2 (379.7)	10G* (12G)	0	355.0	13G	1
	N253.4	370.2	10C	0	375.9	9G	0	256.3 (380.4)	10G (13G)	0	355.0	13G	1
	N253.5	370.0	10C	0	375.7	9G	0	256.3 (379.7)	10G (12G)	0	354.9	13G	1
	N253.6	370.1	10C	0	376.0	9G	0	256.5 (379.4)	10G (12G)	0	354.9	13G	1
V59/3rd	N352.1	370.2	10C	0	375.6	9G	0	256.3 (380.5)	10G (13G)	0	355.0	13G	1
	N352.2	371.0	11C	1	376.0	9G	0	256.2 (380.6)	10G (13G)	0	355.0	13G	1
	N352.3	370.1	10C	0	375.7	9G	0	256.0 (380.6)	10G (13G)	0	355.1	13G	1
	N352.4	370.0	10C	0	376.0	9G	0	256.2 (379.6)	10G (12G)	0	355.0	13G	1
	N352.5	370.1	10C	0	376.6	10G	1	256.3 (380.5)	10G (13G)	0	354.9	13G	1
	N352.6	370.1	10C	0	375.8	9G	0	256.1 (379.4)	10G (12G)	0	355.0	13G	1
V59/4th	N438.1	370.0	10C	0	375.9	9G	0	256.4 (380.4)	10G (13G*)	0	355.0	13G	1
	N438.2	370.1	10C	0	376.1	9G	0	256.3 (380.4)	10G (13G)	0	355.0	13G	1
	N438.3	370.2	10C	0	375.7	9G	0	256.3 (380.5)	10G* (13G)	0	354.0	12G	0
	N438.4	370.1	10C	0	376.0	9G	0	256.3 (380.5)	10G (13G)	0	354.9	13G	1
	N438.5	370.1	10C	0	375.7	9G	0	256.4 (380.6)	10G (13G)	0	355.1	13G	1
	N438.6	370.2	10C	0				256.4 (380.4)	10G (13G)	0	354.9	13G	1
V88/1st	N88.1.1	372.1	12C	0	376.7	10G	1	259.5	13G	1	351.4	9G*	0
	N88.1.2	372.0	12C	0	377.0	10G	1	259.4	13G	1	351.4	9G	0

	N88.1.3	372.0	12C	0	376.7	10G	1	259.6	13G	1	351.4	9G	0
	N88.1.4	372.0	12C	0	377.0	10G	1	259.5	13G	1	351.4	9G	0
	N88.1.5	372.0	12C	0	376.7	10G	1	258.7	12G	0	351.3	9G	0
	N88.1.6	371.9	12C	0	376.8	10G	1	259.7	13G	1	351.3	9G	0
V88/2nd	N272.1	371.9	12C	0	376.8	10G	1	259.6	13G	1	352.3	10G	1
	N272.2	372.0	12C	0	376.9	10G	1	259.5	13G	1	352.3	10G	1
	N272.3	372.1	12C	0	376.9	10G	1	259.5>258.6 {1}	13G	1	352.3	10G	1
	N272.4	372.5	12C	0	377.1	10G	1	259.5	13G	1	352.4	10G	1
	N272.5	372.0	12C	0	376.7	10G	1	259.5	13G	1	352.3	10G	1
	N272.6	372.0	12C	0	377.1	10G	1	256.1	14G	0	355.0	13G	1
V88/3rd	N369.1	372.0	12C	0	376.9	10G	1	259.6>258.7 {1.2}	13G	1	352.3	10G	1
	N369.2	372.0	12C	0	377.2	10G	1	259.6	13G	1	352.3	10G	1
	N369.3	372.1	12C	0	377.7	11G	0	256.4	14G	0	350.2	10G	1
	N369.4	372.0	12C	0	378.1	11G	0	256.4	14G	0	352.4	10G	1
	N369.5	372.0	12C	0	376.8	10G	1	259.4>258.5 {1.2}	13G	1	352.3	10G	1
	N369.6	371.9	12C	0	377.1	10G	1	259.4	13G	1	352.4	10G	1
V88/4th	N449.1	372.0	12C	0	377.9	11G	0	255.3	13G	1	350.3	10G	1
	N449.2	372.0	12C	0	378.1	11G	0	255.5	13G	1	350.4	10G	1
	N449.3	372.0	12C	0	377.9	11G	0	255.4	13G	1	350.4	10G	1
	N449.4	371.6	11C	1	378.1	11G	0	255.4	13G	1	350.4	10G	1
	N449.5	372.0	12C	0	377.9	11G	0	255.4	13G	1	350.4	10G	1
	N449.6	372.0	12C	0	378.1	11G	0	255.4	13G	1	350.2	10G	1
V138/1st	N138.1	370.1	10C	0	375.7	9G	0	256.1 (379.4)	10G (12G)	0	353.3	13G	1
	N138.2	370.1	10C	0	376.0	9G	0	256.0 (380.5)	10G* (13G)	0	355.1	13G*	1
	N138.3	370.0	10C	0	375.9	9G	0	256.0 (380.5)	10G (13G)	0	353.2	13G	1
	N138.4	370.0	10C	0	376.2	9G	0	256.0 (380.5)	10G (13G)	0	355.1	13G	1
	N138.5	370.1	10C	0	375.7	9G	0	256.0 (380.5)	10G (13G)	0	355.2	13G	1
	N138.6	370.2	10C	0	376.1	9G	0	256.1 (381.6)	10G (14G)	1	355.2	13G	1

V138/2nd	N288.1	368.9	10C	0	375.7	9G	0	256.2 (380.6)	10G* (13G)	0	355.0	13G*	1
	N288.2	370.0	10C	0	376.0	9G	0	256.2 (ND)	10G (ND)	ND	355.2	13G	1
	N288.3	370.1	10C	0	375.7	9G	0	256.2 (381.4)	10G (14G)	1	355.1	13G	1
	N288.4	369.9	10C	0	376.2	9G	0	256.0 (380.4)	10G (13G)	0	355.1	13G	1
	N288.5	370.1	10C	0	375.9	9G	0	256.0 (381.5)	10G (14G)	1	353.3	13G	1
	N288.6	370.1	10C	0	376.2	9G	0	256.1 (380.4)	10G (13G)	0	355.0	13G	1
V138/3rd	N331.1	370.1	10C	0	375.8	9G	0	255.9 (379.6)	10G (12G)	0	355.2	13G	1
	N331.2	370.0	10C	0	376.1	9G	0	256.0 (380.4)	10G (13G)	0	355.1	13G	1
	N331.3	370.1	10C	0	375.6	9G	0	256.0 (380.3)	10G (13G)	0	355.2	13G	1
	N331.4	370.0	10C	0	375.8	9G	0	256.1 (380.5)	10G (13G)	0	355.0	13G	1
	N331.5	369.7	10C*	0	375.7	9G	0	256.1 (380.4)	10G (13G*)	0	355.1	13G	1
	N331.6	370.0	10C	0	376.0	9G	0	256.2 (380.4)	10G (13G)	0	355.0	13G	1
V138/4th	Replaced												
						**cc167**							
V124/1st	N124.1	368.9	9C	0	377.9	11G	0	274.2	13G	1	386.0	11G	0
	N124.2	369.2	9C	0	378.2	11G	0	274.3	13G	1	386.1	11G	0
	N124.3	369.0	9C	0	378.0	11G	0	274.4	13G	1	386.2	11G	0
	N124.4	369.1	9C	0	378.2	11G	0	274.2	13G	1	385.2	10G	1
	N124.5	369.0	9C	0	378.0	11G	0	274.2	13G	1	385.1	10G	1
	N124.6	369.2	9C	0	378.3	11G	0	271.7	13G*	1	385.3	10G	1
V124/2nd	N290.1	369.0	9C	0	377.9	11G	0	274.1	13G*	1	386.2	11G	0
	N290.2	369.1	9C*	0	378.3*	11G	0	274.2	13G	1	385.3	10G	1
	N290.3	368.7	9C	0	377.9	11G	0	274.2	13G	1	385.2	10G	1
	N290.4	369.1	9C	0	378.3	11G	0	274.3	13G	1	386.2	11G	0
	N290.5	368.9	9C	0	377.9	11G	0	273.4	12G	0	385.1	10G	1

	N290.6	369.2	9C	0	378.3	11G	0	271.0	10G	1	384.4	9G	0
V124/3rd	N336.1	369.0	9C	0	376.7	10G	1	273.3	12G	0	385.0	10G	1
	N336.2	369.1	9C	0	378.2	11G	0	274.2	13G	1	385.2	10G	1
	N336.3	369.0	9C	0	376.9	10G	1	273.3	12G	0	385.1	10G	1
	N336.4	369.0	9C	0	378.3	11G	0	273.3	12G	0	385.2	10G	1
	N336.5	369.1	9C	0	377.1	10G	1	272.4	11G	0	385.3	10G	1
	N336.6	369.3	9C	0	377.3	10G	1	273.3	12G	0	385.3	10G	1
V64/1st	N64.1	369.1	9C	0	377.9	11G	0	275.2	14G*	0	385.1	10G	1
	N64.2	369.1	9C	0	378.2	11G	0	274.3	13G*	1	385.1	10G*	1
	N64.3	368.9	9C	0	378.0	11G	0	274.2	13G	1	385.3	10G	1
	N64.4	369.1	9C	0	378.3	11G	0	274.1	13G	1	385.0	10G	1
	N64.5	368.9	9C	0	377.9	11G	0	274.2	13G	1	385.1	10G	1
	N64.6	369.1	9C	0	378.2	11G	0	274.2	13G	1	385.1	10G	1
V64/2nd	N257.1	368.9	9C	0	377.8	11G	0	274.2	13G	1	385.1	10G*	1
	N257.2	369.2	9C	0	378.1	11G	0	274.3	13G	1	385.2	10G	1
	N257.3	369.1	9C	0	377.9	11G	0	274.3	13G	1	384.9	10G	1
	N257.4	369.2	9C	0	377.0	10G	1	275.2	14G	0	385.0	10G	1
	N257.5	369.0	9C	0	378.0	11G	0	274.3	13G	1	384.9	10G	1
	N257.6	369.1	9C	0	377.1	10G	1	275.3	14G	0	384.6	10G	1
V64/3rd	N348.1	369.0	9C	0	377.9	11G	0	275.2	14G	0	385.2	10G	1
	N348.2	369.2	9C	0	378.2	11G	0	275.2	14G	0	385.2	10G	1
	N348.3	368.9	9C	0	378.3	11G	0	272.9	14G	0	384.5	10G	1
	N348.4	369.1	9C	0	380.4	13G	1	273.7	15G	0	384.3	10G	1
	N348.5	368.9	9C	0	377.4	11G	0	275.1	14G	0	385.0	10G	1
	N348.6	369.1	9C	0	378.3	11G	0	272.7	14G	0	384.2	10G	1
V117/1st	N117.1	369.0	9C	0	378.4	11G	0	274.3	13G*	1	384.1	9G	0
	N117.2	369.1	9C	0	378.4	11G	0	274.3	13G*	1	384.8	10G*	1
V117/2nd	N284.1	368.9	9C	0	378.2	11G	0	273.3	12G	0	385.3	10G	1
	N284.2	369.2	9C	0	378.3	11G	0	273.3	12G	0	385.3	10G	1
	N284.3	369.0	9C	0	378.3	11G	0	274.3	13G	1	384.2	9G	0
	N284.4	369.2	9C	0	377.4	11G	0	273.3	12G*	0	385.0	10G	1
	N284.5	369.0	9C	0	378.2	11G	0	274.3	13G	1	383.8	9G	0
	N284.6	369.1	9C	0	378.1	11G	0	273.2	12G	0	384.7	10G	1
V117/3rd	N332.1	369.0	9C	0	377.0	10G	1	273.3	12G	0	385.1	10G	1
	N332.2	369.3	9C	0	377.4	10G	1	273.4	12G	0	385.1	10G	1

	N332.3	368.9	9C	0	377.2	10G	1	270.9	12G	0	384.2	10G	1
	N332.4	369.0	9C	0	378.3	11G	0	271.8	13G	1	384.2	10G*	1
	N332.5	369.0	9C	0	378.3	11G	0	274.2	13G	1	385.3	10G	1
	N332.6	369.1	9C	0	379.3	12G	0	274.2	13G	1	385.1	10G	1
V117/4th	N417.1	369.0	9C	0	379.4	12G	0	274.2	13G	1	385.3	10G	1
	N417.2	369.2	9C	0	379.3	12G	0	274.2	13G	1	385.3	10G	1
	N417.3	369.0	9C	0	379.3	12G	0	272.9>271.9 {1.1}	14G	0	384.5	10G*	1
	N417.4	369.1	9C	0	379.2	12G	0	272.9>271.9 [1.1}	14G	0	384.6	10G	1
	N417.5	369.0	9C	0	379.3	12G	0	275.2	14G	0	385.0	10G	1
	N417.6	369.2	9C	0	379.3	12G	0	275.2	14G	0	384.6	10G	1
**cc23**													
V69/1st	Non-carrier												
V69/2nd	N258.1	370.9	11C	1	379.6	13G	1	277.6	12G*	1	424.6	8G*	0
	N258.2	370.6	11C	1	379.5	13G	1	279.6	12G	1	425.2	8G	0
	N258.3	370.9	11C	1	380.3	13G	1	279.7	12G	1	425.3	8G	0
	N258.4	370.8	11C	1	380.2	13G	1	279.7	12G	1	425.4	8G	0
	N258.5	371.0	11C	1	379.5	13G	1	279.6	12G	1	425.3	8G	0
	N258.6	370.6	11C	1	379.3??	13G	1	279.6	12G	1	425.4	8G	0
V69/3rd	No sample												
V69/4th	N431.1	370.7	11C	1	381.7	15G	0	276.6	11G	0	425.7	9G*	1
	N431.2	370.9	11C	1	380.5	14G	0	277.7	12G*	1	425.5	9G	1
	N431.3	370.8	11C	1	380.7	14G	0	279.6	12G	1	426.3	9G	1
	N431.4	370.9	11C	1	381.2	14G	0	279.6	12G	1	426.4	9G	1
	N431.5	370.7	11C	1	381.4>380.5 {1.2}	15G	0	278.8	11G	0	425.7	9G	1
	N431.6	370.9	11C	1	381.1	14G	0	278.7	11G	0	426.3	9G	1
V93/1st	Non-carrier												
V93/2nd	N264.1	370.8	11C	1	378.0	11G	0	281.5>282.4 {1.1}	14G	0	428.3	11G	0
	N264.2	370.9	11C	1	378.3	11G	0	281.4>282.4 {1.1}	14G*	0	428.3	11G*	0
	N264.3	371.7	12C	0	377.3	10G	1	281.4>282.3 {1.1}	14G	0	429.3	12G	1
	N264.4	370.9	11C	1	377.9	11G	0	281.3>282.3	14G	0	428.3	11G	0

								{1.0}					
	N264.5	370.7	11C	1	378.3	11G	0	281.4>282.3<br>{1.1}	14G	0	428.3	11G	0
	N264.6	370.8	11C	1	378.2	11G	0	281.4>282.3<br>{1.0}	14G	0	429.3	12G	1
V93/3rd	N359.1	370.8	11C	1	377.1	10G	1	283.2>282.3<br>{1.0}	15G	1	429.3	12G	1
	N359.2	370.9	11C	1	377.3	10G	1	282.3>283.2<br>{1.2}	15G	1	429.4	12G	1
	N359.3	370.8	11C	1	377.2	10G	1	282.3>283.3<br>{1.1}	15G	1	429.3	12G	1
	N359.4	370.9	11C	1	376.9	10G	1	282.3>283.3<br>{1.1}	15G	1	429.3	12G	1
	N359.5	370.7	11C	1	376.9	10G	1	282.3>283.3<br>{1.1}	15G	1	429.3	12G	1
	N359.6	370.9	11C	1	376.9	10G	1	282.4>283.3<br>{1.2}	15G	1	429.2	12G	1
V96/1st	Non-carrier												
V96/2nd	N259.1	370.7	11C	1	380.2	13G	1	279.6	12G	1	426.3	9G	1
	N259.2	370.9	11C	1	379.5	13G	1	279.6	12G	1	426.5	9G	1
	N259.3	371.7	12C	0	378.1	11G	0	279.6	12G	1	427.2	10G	0
	N259.4	370.9	11C	1	380.2	13G	1	279.6	12G	1	426.3	9G	1
	N259.5	370.7	11C	1	379.3	12G	0	279.6	12G	1	426.4	9G	1
	N259.6	371.8	12C	0	377.1	11G	0	279.7	12G	1	427.3	10G	0
V96/3rd	N370.1	372.2	12C	0	381.5>382.5<br>{1}	15G	0	277.9	10G*	0	426.3	9G	1
	N370.2	369.8	11C	1	380.4>379.4<br>{1.2}	14G	0	278.8	11G	0	426.3	9G	1
	N370.3	371.8>3<br>70.8{1.1}	13C	0	378.3	11G	0	278.7	11G	0	431.3>432.4<br>{1.1}	14G	0
	N370.4	371.8>3<br>70.9{1.1}	13C	0	381.6>382.6<br>{1}	15G	0	278.7	11G	0	426.4	9G	1
	N370.5	370.8	11C	1	379.3	13G	1	276.9	9G	1	425.4	8G	0
	N370.6	370.0	12C*	0	381.6>382.6<br>{1}	15G	0	277.7	10G	0	426.4	9G	1
V96/4th	N445.1	372.3	12C	0	379.1	12G	0	278.8	11G	0	426.4	9G	1
	N445.2	374.8	16C	0	379.6	13G	1	276.6	11G	0	426.4	9G	1

	N445.3	375.7>376.7 {1}	17C	1	379.3	12G	0	278.8	11G	0	426.3	9G	1
	N445.4	374.8>375.7{1.1}	16C	0	379.1	12G	0	278.7	11G	0	426.3	9G	1
	N445.5	371.0>371.9 {1}	12C	0	378.3	11G	0	280.7>279.7 {1.1}	13G	0	426.3	9G	1
	N445.6	372.5	12C	0	377.2	10G	1	278.8	11G	0	426.2	9G	1
V222/1st	N222.1	370.8	11C	1	379.1	13G	1	279.6	12G*	1	425.4 (8)	8G	0
	N222.2	370.7	11C*	1	380.2	13G	1	280.5 (13)	13G	0	426.4 (9)	9G*	1
	N222.3	371.8	12C*	0	379.3	12G	0	279.6 (12)	12G	1	425.4 (8)	8G	0
	N222.4	370.8	11C	1	379.4	13G	1	279.7 (12)	12G	1	425.4 (8)	8G	0
	N222.5	370.8	11C	1	379.5	13G	1	279.6 (12)	12G	1	425.2 (8)	8G	0
	N222.6	371.8	12C	0	379.1	12G	0	279.9 (12)	12G	1	425.3 (8)	8G	0
V222/2nd	N309.1	370.8	11C	1	379.2	13G	1	279.7 (12)	12G	1	426.4 (9)	9G	1
	N309.2	370.8	11C	1	380.2	13G	1	279.7 (12)	12G	1	426.3 (9)	9G	1
	N309.3	370.9	11C	1	380.3	13G	1	279.6 (12)	12G	1	426.4 (9)	9G	1
	N309.4	370.7	11C	1	380.2	13G	1	279.6 (12)	12G	1	426.5 (9)	9G	1
	N309.5	370.8	11C	1	380.5	14G	0	279.5 (12)	12G	1	426.5 (9)	9G	1
	N309.6	370.7	11C	1	380.2	13G	1	279.6 (12)	12G	1	426.5 (9)	9G	1
V222/3rd	N391.1	370.9	11C	1	379.3	13G	1	279.5 (12)	12G	1	426.5 (9)	9G	1
	N391.2	370.6	11C	1	379.6	13G	1	279.6 (12)	12G	1	426.5 (9)	9G	1
	N391.3	370.8	11C	1	380.2	13G	1	279.6 (12)	12G	1	426.3 (9)	9G	1
	N391.4	370.7	11C	1	380.1	13G	1	279.6 (12)	12G	1	426.3 (9)	9G	1
	N391.5	370.9	11C	1	380.1	13G	1	279.6 (12)	12G	1	426.4 (9)	9G	1
	N391.6	370.7	11C	1	380.1	13G	1	277.7 (12)	12G	1	425.5 (9)	9G	1
V222/4th	N459.1	370.8	11C	1	379.1	12G	0	278.7	11G	0	426.5 (9)	9G	1
	N459.2	370.9	11C	1	379.1	12G	0	278.6 (11)	11G	0	426.6 (9)	9G	1
	N459.3	370.8	11C	1	379.1	12G	0	278.5 (11)	11G	0	426.3 (9)	9G	1
	N459.4	370.8	11C	1	379.1	12G	0	279.5 (12)	12G	1	426.2 (9)	9G	1
	N459.5	371.0	11C	1	379.2	12G	0	278.7 (11)	11G	0	426.5 (9)	9G	1
	N459.6	370.7	11C	1	379.1	12G	0	277.5 (12)	12G	1	425.4 (9)	8G	0
**cc60**													
V114/1st	N114.1	370.6	11C	1	375.1	7G	0	274.8	10G*	1	401.9	9G*	0
	N114.2	370.8	11C	1	375.3	7G	0	274.7	10G	1	401.0	8G	0

	N114.3	370.7	11C	1	375.2	7G	0	274.8	10G	1	401.0	8G	0
	N114.4	370.9	11C	1	375.4	7G	0	274.7	10G	1	401.1	8G	0
	N114.5	370.7	11C	1	375.2	7G	0	274.8	10G	1	400.7	8G	0
	N114.6	370.9	11C	1	375.4	7G	0	274.7	10G	1	401.1	8G	0
V114/2nd	N283.1	369.9	11C*	1	375.2	7G	0	274.9	10G	1	400.9	8G	0
	N283.2	369.9	11C	1	375.3	7G	0	274.7	10G	1	401.1	8G	0
	N283.3	369.7	11C	1	375.0	7G	0	272.2	10G	1	401.7	10G*	1
	N283.4	370.8	11C	1	375.3	7G	0	272.3	10G	1	401.6	10G	1
	N283.5	370.4	10C?	0	374.9	7G*	0	275.0	10G*	1	400.6	9G	0
	N283.6	370.6	11C	1	375.1	7G	0	274.7	10G	1	400.9	8G	0
V114/3rd	N330.1	372.7	13C*	0	375.2	7G	0	273.1	8G	0	402.0	9G	0
	N330.2	370.0	10C	0	375.5	7G	0	272.9	8G	0	402.1	9G	0
	N330.3	370.0	10C*	0	375.3	7G	0	272.9	8G	0	401.9	9G	0
	N330.4	370.0	10C	0	375.4	7G	0	272.8	8G	0	402.2	9G	0
	N330.5	370.9	11C	1	375.4	7G	0	273.1	8G	0	402.0	9G	0
	N330.6	370.9	11C	1	375.5	7G	0	272.8	8G	0	402.0	9G	0
V134/1st	N134.1	370.7	11C	1	375.4	7G	0	274.8	10G	1	400.9	8G	0
	N134.2	370.9	11C*	1	375.4	7G	0	274.6	10G	1	402.1	9G	0
	N134.3	370.6	11C	1	375.2	7G	0	274.9	10G*	1	402.0	9G	0
	N134.4	370.8	11C	1	375.3	7G	0	274.7	10G	1	402.1	9G	0
	N134.5	370.6	11C	1	375.1	7G	0	274.9	10G	1	401.9	9G	0
	N134.6	370.7	11C	1	375.3	7G	0	274.7	10G	1	401.9	9G	0
V134/2nd	N295.1	370.5	11C?	1	375.1	7G	0	274.7	10G	1	401.0	8G	0
	N295.2	370.6	11C	1	375.4	7G	0	272.4	10G	1	400.5	9G*	0
	N295.3	370.6	11C	1	375.3	7G	0	274.8	10G	1	401.0	8G	0
	N295.4	370.5	11C	1	375.4	7G	0	274.6	10G	1	401.1	8G	0
	N295.5	370.4	11C?	1	375.3	7G	0	274.8	10G	1	400.9	8G	0
	N295.6	369.9	11C	1	375.4	7G	0	274.7	10G	1	401.1	8G	0
V134/3rd	N333.1	370.9	11C	1	375.2	7G	0	274.8	10G	1	401.9	9G	0
	N333.2	370.8	11C	1	375.5	7G	0	274.6	10G	1	402.0	9G	0
	N333.3	370.9	11C	1	375.3	7G	0	274.9	10G	1	401.8	9G	0
	N333.4	371.0	11C	1	375.5	7G	0	274.6	10G	1	402.1	9G	0
	N333.5	370.9	11C	1	375.2	7G	0	275.0	10G	1	401.9	9G	0
	N333.6	371.0	11C	1	375.3	7G	0	274.7	10G	1	401.8	9G	0
V185/1st	N185.1	371.0	11C*	1	376.2	8G	1	277.7	13G	1	402.9	10G	1

	N185.2	370.8	11C	1	376.4	8G	1	277.7	13G	1	402.6	10G	1
	N185.3	370.9	11C	1	376.3	8G	1	276.9	12G	0	403.8	11G*	0
	N185.4	370.9	11C	1	376.5	8G	1	277.5	13G	1	403.0	10G	1
	N185.5	370.9	11C	1	376.3	8G	1	277.5	13G	1	403.0	10G	1
	N185.6	370.9	11C	1	376.0	8G	1	275.4	13G	1	401.8	10G	1
V185/2nd	N306.1	370.8	11C	1	376.2	8G	1	277.6	13G	1	402.8	10G	1
	N306.2	370.8	11C	1	376.4	8G	1	277.7	13G	1	402.9	10G	1
	N306.3	370.9	11C	1	376.3	8G	1	277.7	13G	1	402.8	10G	1
	N306.4	370.9	11C	1	376.4	8G	1	277.5	13G	1	403.1	10G	1
	N306.5	370.8	11C	1	376.2	8G	1	277.4	13G	1	403.0	10G	1
	N306.6	370.8	11C	1	376.4	8G	1	277.6	13G	1	402.9	10G	1
V185/4th	N456.1	371.8	12C	0	376.2	8G	1	277.6	13G	1	401.9	9G	0
	N456.2	371.8	12C	0	376.3	8G	1	277.6	13G	1	401.8	9G	0
	N456.3	371.9	12C*	0	376.3	8G	1	277.7	13G	1	402.8	10G	1
	N456.4	371.8	12C	0	376.4	8G	1	277.5	13G	1	402.1	9G	0
	N456.5	371.7	12C	0	376.3	8G	1	274.4	13G*??	1	401.9	9G	0
	N456.6	371.7	12C	0	376.5	8G	1	277.5	13G	1	402.0	9G	0
V113/1st	N113.1	369.8	11C*	1	376.2	8G	1	274.8	10G	1	401.9	9G	0
	N113.2	369.9	11C	1	376.4	8G	1	274.7	10G	1	401.0	8G	0
	N113.3	369.8	11C	1	376.4	8G	1	274.8	10G	1	401.0	8G	0
	N113.4	369.8	11C	1	376.4	8G	1	274.7	10G	1	401.1	8G	0
	N113.5	369.6	11C	1	376.2	8G	1	274.8	10G	1	400.7	8G	0
	N113.6	369.7	11C	1	376.4	8G	1	274.7	10G	1	401.1	8G	0
V113/2nd	N281.1	369.7	11C	1	376.2	8G	1	274.7	10G	1	401.9	9G	0
	N281.2	369.8	11C	1	376.3	8G	1	274.6	10G	1	401.1	8G	0
	N281.3	369.9	11C	1	376.3	8G	1	274.9	10G	1	401.9	9G	0
	N281.4	369.9	11C	1	375.3	7G	0	273.8	9G	0	402.1	9G	0
	N281.5	369.8	11C	1	376.2	8G	1	274.9	10G	1	400.8	8G	0
	N281.6	369.8	11C	1	376.4	8G	1	274.7	10G	1	400.9	8G	0
V115/1st	N115.1	369.9	11C	1	375.2	7G	0	274.9	10G	1	400.9	8G	0
	N115.2	369.8	11C	1	374.9	7G	0	274.7	10G	1	401.1	8G	0
	N115.3	369.8	11C	1	375.3	7G	0	274.9	10G	1	400.7	8G	0
	N115.4	369.8	11C	1	375.3	7G	0	274.8	10G	1	400.9	8G	0
	N115.5	369.9	11C	1	375.5	7G?	0	275.0	10G	1	401.9	9G	0
	N115.6	369.9	11C	1	375.6	7G?	0	274.7	10G	1	401.7	9G	0

V115/2nd	N282.1	369.8	11C	1	374.8	7G	0	274.9	10G	1	401.8	9G	0
	N282.2	369.9	11C	1	374.8	7G	0	274.7	10G	1	401.0	8G	0
	N282.3	370.0	11C	1	375.0	7G	0	274.8	10G	1	402.0	9G	0
	N282.4	369.9	11C	1	374.8	7G	0	272.2	10G	1	401.9	10G	1
	N282.5	370.0	11C	1	375.0	7G	0	274.8	10G	1	401.9	9G	0
	N282.6	369.9	11C	1	374.9	7G	0	274.7	10G	1	402.1	9G	0
**cc32**													
V176/1st	N176.1	369.8	10C	0	375.1	8G	0	278.1	13G*	1	429.4	12G*	1
	N176.2	369.6	10C	0	375.2	8G	0	278.0	13G	1	429.3	12G	1
	N176.3	369.8	10C	0	375.2	8G	0	278.0	13G	1	429.4	12G	1
	N176.4	369.6	10C	0	375.0	8G	0	278.0	13G	1	429.4	12G	1
	N176.5	369.7	10C	0	374.8	8G	0	278.1	13G	1	429.4	12G	1
	N176.6	369.6	10C	0	374.9	8G	0	278.1	13G	1	429.3	12G	1
V176/2nd	N318.1	369.7	10C	0	374.8	8G	0	278.0	13G	1	429.4	12G	1
	N318.2	369.6	10C	0	374.9	8G	0	277.2	12G	0	429.3	12G	1
	N318.3	369.0	9C	0	375.0	8G	0	278.0	13G	1	429.3	12G	1
	N318.4	369.7	10C	0	374.8	8G	0	277.9	13G	1	429.4	12G	1
	N318.5	369.8	10C	0	374.9	8G	0	277.4	12G	0	429.4	12G	1
	N318.6	369.6	10C	0	374.8	8G	0	277.0	12G	0	429.5	12G	1
V176/3rd	N399.1	369.8	10C	0	374.9	8G	0	277.0	12G*	0	429.4	12G*	1
	N399.2	369.5	10C	0	374.7	8G	0	277.1	12G	0	429.4	12G	1
	N399.3	369.8	10C	0	374.8	8G	0	277.1	12G	0	429.3	12G	1
	N399.4	369.5	10C	0	374.7	8G	0	277.2	12G	0	429.3	12G	1
	N399.5	369.7	10C	0	374.9	8G	0	277.1	12G	0	429.3	12G	1
	N399.6	369.7	10C	0	374.8	8G	0	277.2	12G	0	429.3	12G	1
V176/4th	N408.1	369.9	10C	0	375.1	8G	0	277.3	12G*	0	429.2	12G*	1
	N408.2	369.6	10C	0	374.8	8G	0	277.2	12G	0	429.2	12G	1
	N408.3	369.8	10C	0	374.9	8G	0	277.2	12G	0	429.3	12G	1
	N408.4	369.7	10C	0	374.9	8G	0	277.2	12G	0	429.3	12G	1
	N408.5	369.9	10C	0	375.1	8G	0	277.2	12G	0	429.4	12G	1
	N408.6	369.6	10C	0	374.9	8G	0	277.2	12G	0	429.3	12G	1

[1]Fragments sizes were determined using either a GS600LIZ (black type) or GS500LIZ (blue type) size standard; numbers in curly brackets indicate the ratio between the two major peaks where obvious multiple peaks were present (the largest peak was utilized if the ratio was >1.2; the larger repeat number equivalent peak was utilized if the ratio was <1.2); numbers in round brackets indicate the repeat number as determined from next generation whole genome sequence data; [2]an asterisk indicates that the PCR fragment was sequenced by dideoxy sequencing and the repeat number determined by visual inspection of the trace file. [3]Expression states, 1=1, 0=0 (all genes are subject to translational phase variation). [4]A subset of cc174 isolates contained a second polyC tract withing pilC, the data for this tract is indicated in brackets whilst the expression state is a combination of data from both tracts.

Supplementary Data Table II. Repeat numbers and expression states for the phase-variable pilin glycosylation genes

Volunteer/ time pt	Isolate	Phase Variable Gene											
		pglA			*pglE*			*pglH*			*pglI*		
		Size[1]	Tract[2]	Exp. State[3]	Size[1]	Tract[2]	Exp. State[3]	Size[1]	Tract[2]	Exp. State[3]	Size[1]	Tract[2]	Exp. State[3]
**cc174**													
V43/1st	N43.1.1	425.4	12G	0	388.4	4CAAACAA*	1	187.1	14C	0	351.3	12G*	0
	N43.1.2	425.6	12G	0	388.5	4CAAACAA	1	187.1	14C	0	352.5	13G	1
	N43.1.3	425.3	12G	0	388.4	4CAAACAA	1	187.1	14C	0	352.4	13G	1
	N43.1.4	425.6	12G	0	388.7	4CAAACAA	1	187.2*	14C	0	352.5	13G	1
	N43.1.5	425.3	12G	0	388.6	4CAAACAA	1	187.1	14C	0	352.4	13G	1
	N43.1.6	425.6	12G	0	388.5	4CAAACAA	1	187.1	14C	0	352.5	13G	1
V43/2nd	N241.1	426.1	12G	0	562.9	29CAAACAA*	0	187.9	15C	0	349.7 (10)	10G*	1
	N241.2	425.7>426.7 {1.2}	12G	0	562.4	29CAAACAA	0	188.0	15C	0	349.2	10G	1
V43/3rd	N349.1	426.1>427.1 {1.1}	13G	0	562.3	29CAAACAA*	0	188.9>189.9 {1.1}	16C	1	349.7 (10)	10G*	1
	N349.2	426.1>427.1 {1.1}	13G	0	562.1	29CAAACAA	0	189.0	16C	1	349.4	10G	1
	N349.3	425.7>426.7 {1.1}	13G	0	562.1	29CAAACAA	0	189.0	16C	1	349.2	10G	1
	N349.4	426.0>427.0 {1.2}	13G	0	555.6	28CAAACAA	1	189.0	16C	1	349.3	10G	1
	N349.5	425.6	12G	0	570.1	30CAAACAA	0	189.0>189.9 {1.2}	16C	1	349.2	10G	1
	N349.6	426.0>427.0 {1.2}	12G	0	562.8	29CAAACAA	0	189.0>190.0 {1.2}	16C	1	349.3	10G	1
V43/4th	Cleared												
V51/1st	N51.1	424.6 (11)	11G*	1	563.1	29CAAACAA*	0	188.0*	15C	0	349.6 (10)	10G	1
	N51.2	424.3 (11)	11G	1	562.7	29CAAACAA	0	188.0>189.0 {1.2}	15C	0	349.3	10G	1
	N51.3	424.5 (11)	11G*	1	562.6	29CAAACAA	0	188.0	15C	0	349.3	10G	1
	N51.4	424.3 (11)	11G	1	561.8	29CAAACAA	0	188.0	15C	0	349.4	10G	1
	N51.5	424.5 (11)	11G	1	562.6	29CAAACAA	0	188.0>188.9 {1.2}	15C	0	349.4	10G	1
	N51.6	424.3 (11)	11G	1	562.1	29CAAACAA	0	188.1>189 {1.2}	15C	0	349.4	10G	1
V51/2nd	N236.1	424.5	11G	1	563.0	29CAAACAA	0	187.9>188.8 {1.1}	15C	0	349.4	10G	1
	N236.2	424.3 (11)	11G	1	562.2	29CAAACAA	0	187.9	15C	0	349.3	10G	1
	N236.3	424.5 (11)	11G	1	562.7	29CAAACAA	0	187.9>188.9 {1.2}	15C	0	349.3	10G	1

	N236.4	424.2 (11)	11G	1	562.6	29CAAACAA	0	187.9	15C	0	349.7	10G	1
	N236.5	424.5 (11)	11G	1	561.9	29CAAACAA	0	187.9	15C	0	349.4	10G	1
	N236.6	424.2 (11)	11G	1	562.1	29CAAACAA	0	187.9	15C	0	349.1	10G*	1
V51/3rd	N354.1	424.4	11G	1	554.8	28CAAACAA	1	190.9>191.8 {1.2}	18C	0	349.3	10G	1
	N354.2	424.4 (11)	11G	1	554.7	28CAAACAA	1	190.9>191.8 {1.2}* (10)	18C	0	349.5	10G	1
	N354.3	424.5 (11)	11G	1	555.2	28CAAACAA	1	190.9>191.9 {1.1}	18C	0	349.4	10G	1
	N354.4	424.4 (11)	11G	1	555.4	28CAAACAA	1	190.8*	18C	0	349.4	10G	1
	N354.5	424.5 (11)	11G	1	569.8	30CAAACAA	0	188.0	15C	0	349.5	10G	1
	N354.6	424.3 (11)	11G	1	554.9	28CAAACAA	1	190.9>190.0 {1.1}	17C	0	349.5	10G	1
V51/4th	N424.1	423.5	10G	0	583.6 (13)	32CAAACAA	0	188.1	15C	0	349.7 (10)	10G	1
	N424.2	423.5	10G	0	582.9	32CAAACAA	0	187.9	15C	0	349.4	10G	1
	N424.3	423.2	10G	0	583.1	32CAAACAA	0	188.0	15C	0	349.5	10G	1
	N424.4	423.5	10G	0	583.6	32CAAACAA	0	187.9	15C	0	349.5	10G	1
	N424.5	423.2	10G	0	583.0	32CAAACAA	0	187.9>188.8 {1.1}	15C	0	349.3	10G	1
	N424.6	423.4	10G	0	576.5	31CAAACAA	1	188>188.9 {1.2}	15C	0	349.4	10G	1
V52/1st	N52.1	426.2	12G	0	562.3	29CAAACAA	0	189.0>190 {1.2}	16C	1	349.6 (10)	10G	1
	N52.2	425.7	12G	0	562.1	29CAAACAA	0	189.1>190 {1.2}	16C	1	349.3	10G	1
	N52.3	426.0	12G	0	562.6	29CAAACAA	0	189>190 {1.2}	16C	1	349.3	10G	1
	N52.4	425.8	12G	0	562.4	29CAAACAA	0	189>189.9 {1.2}	16C	1	349.4	10G	1
	N52.5	426.1	12G	0	562.4	29CAAACAA	0	189>190 {1.2}	16C	1	349.5	10G	1
	N52.6	425.9	12G	0	562.5	29CAAACAA	0	189.0>189.9 {1.2}	16C	1	349.4	10G	1
V52/2nd	N238.1	427.2	13G	0	533.7	25CAAACAA	1	189.0>189.8 {1.0}	16C	1	349.4	10G	1
	N238.2	427.9	14G	1	533.7	25CAAACAA	1	189>189.9 {1.1}	16C	1	349.4	10G	1
	N238.3	427.1	13G	0	576.3	31CAAACAA	1	188.9>189.9 {1.0}	16C	1	349.4	10G	1
	N238.4	427.1	13G	0	533.6	25CAAACAA	1	189.0	16C	1	349.5	10G	1
	N238.5	427.2	13G	0	533.7	25CAAACAA	1	188.9>189.9 {1.0}	16C	1	349.6	10G	1
	N238.6	427.9>426.8 {1.1}	14G	1	576.9	31CAAACAA	1	189.0	16C	1	349.4	10G	1
V52/3rd	N342.1	427.1	13G	0	577.4	31CAAACAA*	1	188.0	15C	0	351.5	12G	0
V52/4th	Cleared												
V54/1st	N54.1.1	424.6 (11)	11G	1	388.8 (4)	4CAAACAA*	1	186.2	13C	1	349.6	10G	1
	N54.1.2	424.5	11G	1	388.8	4CAAACAA	1	186.3>185.2 {1.1}	12C	0	352.5>351.5 {1.0}	12G	0

	N54.1.3	424.3	11G	1	388.7	4CAAACAA	1	186.2	13C	1	352.5>351.5 {1.2}	13G	1
	N54.1.4	424.5	11G	1	388.7	4CAAACAA	1	186.2>185.2 {1.0}	12C	0	352.5>351.5 {1.1}	12G	0
	N54.1.5	424.4	11G	1	388.7	4CAAACAA	1	186.2>185.1 {1.1}	12C	0	352.5>351.5 {1.0}	12G	0
	N54.1.6	424.5	11G	1	388.8	4CAAACAA	1	186.1>185 {1.2}	13C	1	352.5>351.5 {1.0}	12G	0
V54/2nd	N237.1	424.4	11G	1	388.8	4CAAACAA	1	185.1	12C	0	351.4	12G	0
	N237.2	425.6	12G	0	388.8	4CAAACAA	1	186.1	13C	1	351.5	12G	0
	N237.3	424.4	11G	1	388.7	4CAAACAA	1	184.2	11C	0	351.5	12G	0
	N237.4	424.6	11G	1	388.8	4CAAACAA	1	188>187 {1.1}	14C	0	351.5	12G	0
	N237.5	424.6	11G	1	388.7	4CAAACAA	1	184.1	11C	0	352.3	13G	1
	N237.6	424.5	11G	1	388.8	4CAAACAA	1	187>188 {1.1}	14C	0	352.5	13G	1
V54/3rd	N343.1	424.5 (11)	11G	1	388.6 (4)	4CAAACAA	1	186.2	13C	1	351.5>352.6 {1.2}	12G	0
	N343.2	424.3	11G	1	388.7	4CAAACAA	1	185.1	12C	0	352.4	13G	1
	N343.3	424.5	11G	1	388.7	4CAAACAA	1	186.1	13C	1	352.5	13G	1
	N343.4	424.3	11G	1	388.6	4CAAACAA	1	185.2	12C	0	352.4	13G	1
	N343.5	424.5	11G	1	388.8	4CAAACAA	1	184.2	11C	0	352.5>351.4 {1.1}	12G	0
	N343.6	424.3	11G	1	388.7	4CAAACAA	1	186.1	13C	1	352.4	13G	1
V54/4th	Replaced												
V58/1st	N58.1	426.2	12G	0	562.9 (13)	29CAAACAA	0	188.1	15C	0	349.7 (10)	10G	1
	N58.2	426.1	12G	0	561.3	29CAAACAA	0	188.2	15C	0	349.5	10G	1
	N58.3	426.8>425.8 {1.1}	13G	0	562.2	29CAAACAA	0	188.0	15C	0	349.5	10G	1
	N58.4	426.1	12G	0	561.7	29CAAACAA	0	188.3	15C	0	349.6	10G	1
	N58.5	425.8	12G	0	562.1	29CAAACAA	0	188.2	15C	0	349.5	10G	1
	N58.6	425.7	12G	0	561.8	29CAAACAA	0	188.2	15C	0	349.5	10G	1
V58/2nd	N240.1	427.9	14G	1	562.2	29CAAACAA	0	188.0	15C	0	349.5	10G	1
	N240.2	426.1	12G	0	562.4	29CAAACAA	0	187.9	15C	0	349.6	10G	1
V58/3rd	Cleared												
V58/4th	N429.1	425.9	12G	0	533.8 (13)	25CAAACAA	1	189.0	16C	1	349.7>348.6 {1.2}	10G	1
	N429.2	426.8>425.8 {1.1}	13G	0	533.7	25CAAACAA	1	189.0	16C	1	349.4	10G	1
	N429.3	427.1>426.1 {1.1}	13G	0	518.5	23CAAACAA	0	189.0	16C	1	349.6	10G	1
	N429.4	426.7>425.8 {1.2}	13G	0	518.8	23CAAACAA	0	189.0	16C	1	349.3	10G	1
	N429.5	426.1>427.1	13G	0	533.5	25CAAACAA	1	189.0	16C	1	349.6	10G	1

		{1.0}											
	N429.6	426.8>425.8 {1.1}	13G	0	518.7	23CAAACAA	0	189.1	16C	1	349.4	10G	1
V59/1st	N59.1.1	426.1	12G	0	563.1	29CAAACAA	0	189.9	17C	0	349.6 (10)	10G	1
	N59.1.2	426.0>427.0 {1.1}	13G	0	562.7	29CAAACAA	0	189.9	17C	0	349.3	10G	1
	N59.1.3	425.8	12G	0	556.0	28CAAACAA	0	189.9	17C	0	349.5>348.6 {1.0}	9G	0
	N59.1.4	426.0>427.1 {1.2}	12G	0	562.6	29CAAACAA	0	190.0	17C	0	349.5	10G	1
	N59.1.5	425.8>426.8 {1.1}	13G	0	562.6	29CAAACAA	0	190.0	17C	0	349.4	10G	1
	N59.1.6	426.0>427.1 {1.2}	12G	0	562.3	29CAAACAA	0	190.0	17C	0	349.6	10G	1
	N59.1.7	426.2	12G	0	563.3	29CAAACAA	0	No data			349.6	10G	1
	N59.1.8	426.1	12G	0	562.9	29CAAACAA	0	No data			349.6	10G	1
	N59.1.9	426.1	12G	0	562.7	29CAAACAA	0	No data			349.6	10G	1
	N59.1.10	426.0	12G	0	562.8	29CAAACAA	0	No data			349.6	10G	1
V59/2nd	N253.1	425.8	12G	0	562.3	29CAAACAA	0	190.0>189.0 {1.0}	16C	1	349.5	10G	1
	N253.2	426.0	12G	0	562.2	29CAAACAA	0	189.0>190.0 {1.1}	16C	1	349.4	10G	1
	N253.3	425.6>426.8 {1.0}	13G	0	562.6	29CAAACAA	0	190.0>189.0 {1.1}	16C	1	349.3	10G	1
	N253.4	426.0>427 {1.1}	13G	0	562.3	29CAAACAA	0	189.9>189.0 {1.0}	16C	1	349.3	10G	1
	N253.5	425.6>426.6 {1.1}	13G	0	562.9	29CAAACAA	0	189.9>189.0 {1.1}	16C	1	349.4	10G	1
	N253.6	426.0>427 {1.1}	13G	0	562.7	29CAAACAA	0	190.0>189.0 {1.0}	16C	1	349.3	10G	1
	N253.7	426.0>427.1 {1.1}	13G	0	562.5	29CAAACAA	0	No data			348.5>349.6 {1.1}	9G	0
	N253.8	426.1>427.1 {1.1}	13G	0	562.9	29CAAACAA	0	No data			348.5>349.5 {1.1}	9G	0
	N253.9	426.1>427.2 {1.1}	13G	0	562.7	29CAAACAA	0	No data			348.5>349.5 {1.1}	9G	0
	N253.10	426.1>427.2 {1.1}	13G	0	563.1	29CAAACAA	0	No data			348.4>349.5 {1.0}	9G	0
V59/3rd	N352.1	425.7	12G	0	562.5	29CAAACAA	0	189.0>187.8 {1.1}	15C	0	349.3	10G	1
	N352.2	425.9>424.9 {1.2}	12G	0	562.5	29CAAACAA	0	189.0>188.0 {1.1}	15C	0	349.4	10G	1
	N352.3	425.7>426.7	13G	0	562.4	29CAAACAA	0	188.6>187.9 {1.1}	15C	0	349.3	10G	1

		{1.1}											
	N352.4	424.9	11G	1	576.6	31CAAACAA	1	189.0>187.9 {1.1}	15C	0	350.7	11G	0
	N352.5	425.7>426.8 {1.0}	13G	0	562.3	29CAAACAA	0	189.0>187.9 {1.1}	15C	0	349.4	10G	1
	N352.6	426.0>427.0 {1.0}	13G	0	562.9	29CAAACAA	0	189.0>187.9 {1.1}	15C	0	349.5	10G	1
	N352.7	424.9>426.0 {1.1}	12G	0	555.4	28CAAACAA	1	No data			348.4>349.4 {1.0}	9G	0
	N352.8	426.1	12G	0	562.8	29CAAACAA	0	No data			349.5	10G	1
	N352.9	425.1>426.1 {1.1}	12G	0	562.2	29CAAACAA	0	No data			349.5	10G	1
	N352.10	425.2>426.2 {1.1}	12G	0	563.5	29CAAACAA	0	No data			349.5	10G	1
V59/4th	N438.1	426.0	12G	0	562.7 (13)	29CAAACAA	0	188.0>188.9 {1.0}	15C	0	349.7 (10)	10G	1
	N438.2	425.8>426.8 {1.0}	13G	0	562.6	29CAAACAA	0	188.0>188.9 {1.1}	15C	0	349.4	10G	1
	N438.3	427.0>426.0 {1.0}	13G	0	562.4	29CAAACAA	0	187.9>188.9 {1.1}	15C	0	349.4	10G	1
	N438.4	425.7	12G	0	562.5	29CAAACAA	0	188.0>188.9 {1.1}	15C	0	349.4	10G	1
	N438.5	426.0>427.0 {1.2}	12G	0	562.4	29CAAACAA	0	187.9>188.9 {1.1}	15C	0	349.4	10G	1
	N438.6	426.0>427.0 {1.0}	13G	0	562.0	29CAAACAA	0	187.9>188.9 {1.1}	15C	0	349.5	10G	1
	N438.7	426.1>427.2 {1.1}	13G	0	563.3	29CAAACAA	0	No data					
	N438.8	426.0>427.0 {1.1}	13G	0	563.3	29CAAACAA	0	No data			349.4	10G	1
	N438.9	426.0>427.0 {1.1}	13G	0	563.3	29CAAACAA	0	No data			349.6	10G	1
	N438.10	426.1>427.1 {1.1}	13G	0							349.3	10G	1
V88/1st	N88.1.1	427.6	14G*	1	518.7	23CAAACAA*	0	188.9	16C	1	351.6>352.6 {1.0}	12G	0
	N88.1.2	427.6	14G	1	525.8	24CAAACAA	0	188.7	16C	1	352.5>351.4 {1.1}	12G	0
	N88.1.3	427.7	14G	1	518.5	23CAAACAA	0	188.9	16C	1	351.5>352.5 {1.1}	12G	0
	N88.1.4	427.6	14G	1	518.3	23CAAACAA	0	188.9	16C	1	352.5>351.5 {1.1}	12G	0
	N88.1.5	427.5	14G	1	518.5	23CAAACAA	0	189.1	16C	1	351.5>352.5 {1.0}	12G	0

	N88.1.6	427.7	14G	1	518.7	23CAAACAA	0	188.9	16C	1	351.4>352.5 {1.1}	12G	0
V88/2nd	N272.1	426.6	13G*	0	518.7	23CAAACAA	0	188.9	16C	1	352.5>351.5 {1.1}	12G	0
	N272.2	426.6	13G	0	518.7	23CAAACAA	0	188.8	16C	1	351.5>352.5 {1.1}	12G	0
	N272.3	426.6	13G	0	518.4	23CAAACAA	0	188.9	16C	1	352.5>351.5 {1.0}	12G	0
	N272.4	426.6	13G	0	518.6	23CAAACAA	0	189.0	16C	1	351.5>352.5 {1.0}	12G	0
	N272.5	426.6	13G	0	518.6	23CAAACAA	0	188.9	16C	1	351.5>352.5 {1.1}	12G	0
	N272.6	426.7	13G	0	518.7	23CAAACAA	0	188.9	16C	1	351.5>352.6 {1.0}	12G	0
V88/3rd	N369.1	427.8	14G	1	511.1	22CAAACAA	1	188.9	16C	1	351.5>352.5 {1.1}	12G	0
	N369.2	427.8	14G	1	511.3	22CAAACAA	1	189.0	16C	1	351.5>352.6 {1.0}	12G	0
	N369.3	427.7	14G	1	503.6	21CAAACAA	0	189.2	16C	1	351.5>350.6 {1.1}	12G	0
	N369.4	427.7	14G	1	503.6	21CAAACAA	0	189.1	16C	1	351.5>350.6 {1.1}	12G	0
	N369.5	427.7	14G	1	511.3	22CAAACAA	1	188.9	16C	1	352.4>351.5 {1.1}	13G	1
	N369.6	427.7	14G	1	511.2	22CAAACAA	1	189.0>188.0 {1.0}	15C	0	351.5>352.5 {1.0}	13G	1
V88/4th	N449.1	427.7	14G	1	540.7 (13)	26CAAACAA	0	188.9	16C	1	351.6>352.3 (1.2)	13G	1
	N449.2	427.7	14G	1	533.5	25CAAACAA	1	188.9	16C	1	352.5>351.5 {1.1}	13G	1
	N449.3	427.6	14G	1	540.7	26CAAACAA	0	189.9	17C	0	352.5>351.5 {1.1}	13G	1
	N449.4	427.6	14G	1	540.7	26CAAACAA	0	189.0	16C	1	352.5>351.5 {1.1}	13G	1
	N449.5	427.7	14G	1	547.7	27CAAACAA	0	189.0	16C	1	351.5>352.5 {1.0}	13G	1
	N449.6	427.6	14G	1	533.2	25CAAACAA	1	189.0	16C	1	351.5>352.5 {1.1}	13G	1
V138/1st	N138.1	427.1>426.1 {1.1}	13G	0	562.3	29CAAACAA	0	188.9	16C	1	349.5 (10)	10G	1
	N138.2	426.1>427.2 {1.0}	13G	0	562.3	29CAAACAA	0	188.9	16C	1	349.6	10G	1
	N138.3	426.1	12G	0	561.8	29CAAACAA	0	188.9>189.9 {1.1}	16C	1	349.7	10G	1
	N138.4	426.1>427.2 {1.0}	13G	0	562.5	29CAAACAA	0	188.9	16C	1	349.5	10G	1
	N138.5	427.1>426.1 {1.1}	13G	0	562.7	29CAAACAA	0	188.9>189.9 {1.2}	16C	1	349.6	10G	1
	N138.6	426.1	12G	0	561.8	29CAAACAA	0	189.0	16C	1	349.6	10G	1
V138/2nd	N288.1	426.0	12G	0	562.4	29CAAACAA	0	188.9	16C	1	349.4	10G	1
	N288.2	427.1	13G	0	562.6	29CAAACAA	0	188.0>189.0 {1.1}	15C	0	349.5	10G	1
	N288.3	425.0	11G	1	562.2	29CAAACAA	0	188.0	15C	0	349.3	10G	1
	N288.4	427.1	13G	0	561.9	29CAAACAA	0	188.0>188.9 {1.1}	15C	0	349.6	10G	1
	N288.5	426.1	12G	0	561.9	29CAAACAA	0	188.0	15C	0	349.5	10G	1
	N288.6	427.1	13G	0	561.9	29CAAACAA	0	187.9>188.9 {1.2}	15C	0	350.6	11G	0
V138/3rd	N331.1	427.9>426.9	14G*	1	561.9	29CAAACAA	0	188.9>187.9 {1.1}	15C	0	348.2 (9)	9G	0

		{1.2}											
	N331.2	426.0>427.0 {1.1}	13G	0	576.1	31CAAACAA	1	188.9>189.8 {1.2}	16C	1	349.5	10G	1
	N331.3	426>427 {1.1}	13G	0	575.9	31CAAACAA	1	187.9>188.9 {1.1}	15C	0	349.5	10G	1
	N331.4	427.0>426.0 {1.2}	13G	0	575.9	31CAAACAA	1	188.9>187.9 {1.2}	16C	1	349.6	10G	1
	N331.5	426.0>427.1 (1.0)	13G	0	576.3	31CAAACAA	1	187.8>188.9 {1.1}	15C	0	349.4	10G	1
	N331.6	426.0>427.0 {1.1}	13G	0	576.3	31CAAACAA	1	188.9>189.8 {1.2}	16C	1	348.4	9G	0
V138/4th	Replaced												
**cc167**													
V124/1st	N124.1	420.7 (11)	11G	1	484.2	18CAAACAA*	0	187.1	11C	0	349.3 (10)	10G	1
	N124.2	420.7	11G	1	484.3	18CAAACAA	0	187.0	11C	0	349.3	10G	1
	N124.3	420.7	11G	1	484.2	18CAAACAA	0	187.1	11C	0	349.3	10G	1
	N124.4	420.7	11G	1	484.2	18CAAACAA	0	187.0	11C	0	349.3	10G	1
	N124.5	420.6	11G	1	484.1	18CAAACAA	0	187.1	11C	0	349.3	10G	1
	N124.6	420.6	11G	1	484.1	18CAAACAA*	0	186.9	11C	0	349.3	10G	1
V124/2nd	N290.1	420.7	11G	1	484.3	18CAAACAA	0	187.1	11C	0	349.3	10G	1
	N290.2	420.8	11G	1	484.2	18CAAACAA	0	187.0	11C	0	349.4	10G	1
	N290.3	420.8	11G	1	484.3	18CAAACAA	0	187.1	11C	0	349.4	10G	1
	N290.4	420.8	11G	1	491.1	19CAAACAA	1	187.0	11C	0	349.3	10G	1
	N290.5	420.7	11G	1	484.2	18CAAACAA	0	187.1	11C	0	349.3	10G	1
	N290.6	420.7	11G	1	484.2	18CAAACAA	0	187.0	11C	0	349.3	10G	1
V124/3rd	N336.1	420.6 (11)	11G	1	484.2	18CAAACAA	0	187.1	11C	0	349.3 (10)	10G	1
	N336.2	420.7	11G	1	506.3	21CAAACAA	0	187.0	11C	0	349.3	10G	1
	N336.3	420.7	11G	1	484.3	18CAAACAA	0	187.1	11C	0	349.3	10G	1
	N336.4	420.7	11G	1	484.2	18CAAACAA	0	186.9	11C	0	349.3	10G	1
	N336.5	420.7	11G	1	484.3	18CAAACAA	0	187.1	11C	0	349.3	10G	1
	N336.6	420.8	11G	1	484.2	18CAAACAA	0	186.9	11C	0	349.4	10G	1
V64/1st	N64.1	420.6 (11)	11G	1	484.2	18CAAACAA	0	187.0	11C	0	349.3 (10)	10G	1
	N64.2	420.6	11G	1	484.1	18CAAACAA	0	186.9	11C	0	349.3	10G	1
	N64.3	420.7	11G	1	484.2	18CAAACAA	0	187.0	11C	0	349.3	10G	1
	N64.4	420.7	11G	1	484.2	18CAAACAA	0	186.9	11C	0	349.3	10G	1
	N64.5	420.7	11G	1	484.2	18CAAACAA	0	187.1	11C	0	349.3	10G	1
	N64.6	420.7	11G	1	484.3	18CAAACAA	0	186.9	11C	0	349.3	10G	1

V64/2nd	N257.1	420.5	11G	1	484.2	18CAAACAA	0	186.9	11C	0	349.2	10G	1
	N257.2	420.6	11G	1	484.1	18CAAACAA	0	186.8	11C	0	347.5	10G	1
	N257.3	420.6	11G	1	484.2	18CAAACAA	0	187.1	11C	0	349.3	10G	1
	N257.4	420.5	11G	1	484.2	18CAAACAA	0	186.9	11C	0	349.3	10G	1
	N257.5	420.5	11G	1	484.1	18CAAACAA	0	187.1	11C	0	349.3	10G	1
	N257.6	420.5	11G	1	484.1	18CAAACAA	0	186.9	11C	0	349.2	10G	1
V64/3rd	N348.1	420.7	11G	1	477.6	17CAAACAA	0	187.0	11C	0	349.3 (10)	10G	1
	N348.2	420.7	11G	1	484.3	18CAAACAA	0	187.0	11C	0	349.3	10G	1
	N348.3	420.7	11G	1	484.3	18CAAACAA	0	187.0	11C	0	349.2	10G	1
	N348.4	420.7	11G	1	477.6	17CAAACAA	0	186.9	11C	0	349.3	10G	1
	N348.5	420.6	11G	1	491.1	19CAAACAA	1	187.1	11C	0	349.3	10G	1
	N348.6	420.6	11G	1	477.6	17CAAACAA	0	187.0	11C	0	349.3	10G	1
V117/1st	N117.1	420.5 (11)	11G	1	484.1 (13)	18CAAACAA*	0	184.3	11C*	0	349.3 (10)	10G	1
	N117.2	420.6	11G	1	484.2	18CAAACAA*	0	184.2	11C	0	349.3	10G	1
V117/2nd	N284.1	420.6	11G	1	484.1	18CAAACAA	0	184.3	11C	0	349.3	10G	1
	N284.2	420.7	11G	1	484.3	18CAAACAA	0	184.5	11C	0	349.2	10G	1
	N284.3	420.5	11G	1	484.2	18CAAACAA	0	184.4	11C	0	349.2	10G	1
	N284.4	420.3	11G	1	484.6	18CAAACAA*	0	184.3	11C	0	347.0	10G	1
	N284.5	420.6	11G	1	484.1	18CAAACAA	0	184.2	11C	0	349.3	10G	1
	N284.6	420.6	11G	1	484.2	18CAAACAA	0	184.3	11C	0	349.3	10G	1
V117/3rd	N332.1	420.5	11G	1	464.5	15CAAACAA	0	184.2	11C	0	349.3	10G	1
	N332.2	420.5	11G	1	464.6	15CAAACAA	0	184.5	11C*	0	349.3	10G	1
	N332.3	420.5	11G	1	464.6	15CAAACAA	0	184.2	11C	0	349.3	10G	1
	N332.4	420.5	11G	1	464.6	15CAAACAA	0	184.2	11C	0	349.3	10G	1
	N332.5	420.5	11G	1	484.2	18CAAACAA	0	184.2	11C	0	349.3	10G	1
	N332.6	420.6	11G	1	484.3	18CAAACAA	0	184.2	11C	0	349.3	10G	1
V117/4th	N417.1	420.5	11G	1	484.1	18CAAACAA	0	184.4	11C	0	349.2 (10)	10G	1
	N417.2	420.6	11G	1	484.2	18CAAACAA	0	184.4	11C	0	349.3	10G	1
	N417.3	420.5	11G	1	477.7	17CAAACAA	0	184.6	11C	0	349.2	10G	1
	N417.4	420.6	11G	1	477.5	17CAAACAA	0	184.5	11C	0	349.3	10G	1
	N417.5	420.5	11G	1	477.6	17CAAACAA	0	184.5	11C	0	349.2	10G	1
	N417.6	420.6	11G	1	484.2	18CAAACAA	0	184.5	11C	0	349.2	10G	1
**cc23**													
V69/1st	Non-carrier												

V69/2nd	N258.1	417.1 (8)	8G*	1	444.1 (12)	12CAAACAA*	0	187.4>188.4 {1.1}	16C*	1	347.1	10G*	1
	N258.2	417.0	8G	1	444.4	12CAAACAA	0	187.5>188.4 {1.1}	16C	1	347.2	10G	1
	N258.3	417.2	8G	1	444.2	12CAAACAA	0	187.4>188.4 {1.1}	16C	1	347.1	10G	1
	N258.4	417.1	8G	1	444.0	12CAAACAA	0	187.4>188.4 {1.2}	16C	1	347.1	10G	1
	N258.5	417.4	8G	1	444.1	12CAAACAA	0	187.3>188.2 {1.2}	16C	1	347.3	10G	1
	N258.6	417.1	8G	1	444.1	12CAAACAA	0	187.4>188.4 {1.0}	16C	1	347.2	10G	1
V69/3rd	No sample												
V69/4th	N431.1	417.4 (8)	8G	1	444.3 (12)	12CAAACAA	0	187.5>188.6 {1.2}	16C*	1	347.2 (10)	10G	1
	N431.2	417.3	8G	1	444.3	12CAAACAA	0	187.5>188.6 {1.1}	16C	1	347.2	10G	1
	N431.3	417.5	8G	1	444.2	12CAAACAA	0	187.6>188.5 {1.2}	16C	1	347.3	10G	1
	N431.4	417.3	8G	1	444.4	12CAAACAA	0	187.6>188.6 {1.1}	16C	1	347.2	10G	1
	N431.5	417.3	8G	1	444.4	12CAAACAA	0	187.5>188.5 {1.2}	16C	1	347.2	10G	1
	N431.6	417.2	8G	1	444.4	12CAAACAA	0	187.5>188.5 {1.1}	16C	1	347.3	10G	1
V93/1st	Non-carrier												
V93/2nd	N264.1	424.0	15G*??	1??	445.6 (11)	12CAAACAA	0	189.9	16C	1	349.4 (10)	10G	1
	N264.2	424.0	14G	1	445.5	12CAAACAA	0	189.8	16C	1	349.5	10G	1
	N264.3	425.0	15G	0	438.7	11CAAACAA	0	189.9	16C	1	349.4	10G	1
	N264.4	423.8	14G*	1	445.5	12CAAACAA	0	189.9	16C	1	349.5	10G	1
	N264.5	423.7	14G	1	445.4	12CAAACAA	0	189.9	16C	1	349.4	10G	1
	N264.6	424.3	15G	0	445.4	12CAAACAA	0	189.9	16C	1	349.4	10G	1
V93/3rd	N359.1	425.4	16G*	0	445.5	12CAAACAA	0	190.9	17C	0	349.4	10G	1
	N359.2	425.4	16G	0	445.6	12CAAACAA	0	190.9	17C	0	349.4	10G	1
	N359.3	425.3	16G	0	445.4	12CAAACAA	0	190.9	17C	0	349.4	10G	1
	N359.4	425.2	16G	0	445.4	12CAAACAA	0	190.9	17C	0	349.4	10G	1
	N359.5	425.3	16G	0	452.1	13CAAACAA	1	189.9	16C	1	349.5	10G	1
	N359.6	425.3	16G	0	445.5	12CAAACAA	0	190.9	17C	0	349.4	10G	1
V96/1st	Non-carrier												
V96/2nd	N259.1	417.3 (8)	8G*	1	445.5 (12)	12CAAACAA*	0	185.1	11C	0	349.4 (10)	10G*	1
	N259.2	417.4	8G	1	445.5	12CAAACAA	0	185.0	11C	0	349.5	10G	1
	N259.3	417.5	8G	1	445.5	12CAAACAA	0	185.1	11C	0	349.5	10G	1
	N259.4	417.4	8G	1	445.5	12CAAACAA	0	184.9	11C	0	349.4	10G	1

								(13)					
	N259.5	417.4	8G	1	445.5	12CAAACAA	0	185.1	11C	0	349.4	10G	1
	N259.6	417.4	8G	1	445.5	12CAAACAA	0	184.9	11C	0	349.4	10G	1
V96/3rd	N370.1	417.3	8G	1	445.5	12CAAACAA	0	185.1	11C	0	348.5	9G	0
	N370.2	417.3	8G	1	445.4	12CAAACAA	0	185.0 (10)	11C	0	348.4	9G	0
	N370.3	417.4	8G	1	445.4	12CAAACAA	0	185.1	11C	0	351.4	12G	0
	N370.4	417.5	8G	1	445.6	12CAAACAA	0	185.0	11C	0	349.4>350.4 {1.2}	10G	1
	N370.5	417.5	8G	1	445.5	12CAAACAA	0	185.1	11C	0	348.4	9G	0
	N370.6	417.5	8G	1	445.6	12CAAACAA	0	185.0	11C	0	348.4	9G	0
V96/4th	N445.1	417.5 (8)	8G	1	445.5 (11)	12CAAACAA	0	185.2	11C	0	348.4 (9)	9G	0
	N445.2	417.4	8G	1	445.5	12CAAACAA	0	185.3	11C	0	348.5	9G	0
	N445.3	417.4	8G	1	445.5	12CAAACAA	0	185.3	11C	0	348.5	9G	0
	N445.4	417.4	8G	1	445.5	12CAAACAA	0	185.2	11C	0	348.5	9G	0
	N445.5	417.4	8G	1	445.5	12CAAACAA	0	185.1	11C	0	348.5	9G	0
	N445.6	417.4	8G	1	445.6	12CAAACAA	0	185.1	11C	0	349.4	10G	1
V222/1st	N222.1	417.4 (8)	8G	1	445.6 (12)	12CAAACAA	0	190.0	16C	1	349.5	10G	1
	N222.2	417.4	8G	1	445.5	12CAAACAA	0	190.0	16C	1	349.5	10G	1
	N222.3	417.4	8G	1	445.4	12CAAACAA	0	190.0	16C	1	349.4	10G	1
	N222.4	417.4	8G	1	445.5	12CAAACAA	0	190.0	16C	1	349.4	10G	1
	N222.5	417.4	8G	1	445.5	12CAAACAA	0	190.0	16C	1	349.4	10G	1
	N222.6	417.4	8G	1	445.5	12CAAACAA	0	189.9	16C	1	349.5	10G	1
V222/2nd	N309.1	417.4	8G	1	445.5	12CAAACAA	0	189.9	16C	1	349.4	10G	1
	N309.2	417.4	8G	1	445.6	12CAAACAA	0	189.9	16C	1	349.4	10G	1
	N309.3	417.2	8G	1	445.5	12CAAACAA	0	189.9	16C	1	349.4	10G	1
	N309.4	417.3	8G	1	445.5	12CAAACAA	0	189.9	16C	1	349.3	10G	1
	N309.5	417.1	8G	1	445.5	12CAAACAA	0	190.0	16C	1	349.4	10G	1
	N309.6	417.2	8G	1	445.4	12CAAACAA	0	190.0	16C	1	349.4	10G	1
V222/3rd	N391.1	417.3	8G	1	445.5	12CAAACAA	0	189.1	15C	0	349.5	10G	1
	N391.2	417.4	8G	1	445.5	12CAAACAA	0	189.0	15C	0	349.5	10G	1
	N391.3	417.4	8G	1	445.6	12CAAACAA	0	189.0	15C	0	349.5	10G	1
	N391.4	417.4	8G	1	445.5	12CAAACAA	0	189.0	15C	0	349.4	10G	1
	N391.5	417.4	8G	1	445.5	12CAAACAA	0	189.0	15C	0	349.4	10G	1
	N391.6	417.4	8G	1	445.5	12CAAACAA	0	189.0	15C	0	349.4	10G	1

V222/4th	N459.1	417.4 (8)	8G	1	445.5 (11)	12CAAACAA	0	189.0	15C	0	349.5 (10)	10G	1
	N459.2	417.3	8G	1	452.1	13CAAACAA	1	189.1	15C	0	349.4	10G	1
	N459.3	417.3	8G	1	445.4	12CAAACAA	0	189.0	15C	0	349.5	10G	1
	N459.4	417.3	8G	1	445.4	12CAAACAA	0	189.0	15C	0	349.5	10G	1
	N459.5	417.4	8G	1	458.3	14CAAACAA	0	189.0	15C	0	349.4	10G	1
	N459.6	417.4	8G	1	445.5	12CAAACAA	0	189.0	15C	0	349.4	10G	1
**cc60**													
V114/1st	N114.1	424.1	14G*	1	464.6 (12)	15CAAACAA	0	Absent			430.7	13G	1
	N114.2	424.3	14G	1	464.7	15CAAACAA	0	Absent			430.7	13G	1
	N114.3	424.3	14G	1	464.7	15CAAACAA	0	Absent			430.7	13G	1
	N114.4	424.3	14G	1	464.7	15CAAACAA	0	Absent			430.7	13G	1
	N114.5	424.2>423.2 {1.2}	14G	1	464.7	15CAAACAA	0	Absent			430.7	13G	1
	N114.6	424.2	14G	1	464.7	15CAAACAA	0	Absent			430.7	13G	1
V114/2nd	N283.1	424.4	14G	1	464.2	15CAAACAA	0	Absent			430.8	13G	1
	N283.2	424.4	14G	1	464.6	15CAAACAA	0	Absent			430.7	13G	1
	N283.3	425.2>424.3 [1.2]	15G	0	464.7	15CAAACAA	0	Absent			430.8	13G	1
	N283.4	424.4	14G	1	464.6	15CAAACAA	0	Absent			430.7	13G	1
	N283.5	425.5	15G	0	464.3	15CAAACAA	0	Absent			430.7	13G	1
	N283.6	424.4	14G	1	464.2	15CAAACAA	0	Absent			430.7	13G	1
V114/3rd	N330.1	420.2 (10)	10G	0	464.7 (12)	15CAAACAA	0	Absent			427.5	11G	0
	N330.2	420.2	10G	0	464.5	15CAAACAA	0	Absent			427.8	11G	0
	N330.3	420.1	10G	0	464.6	15CAAACAA	0	Absent			429.7	12G	0
	N330.4	420.1	10G	0	464.6	15CAAACAA	0	Absent			428.6	11G	0
	N330.5	420.3	10G	0	464.7	15CAAACAA	0	Absent			433.3??	13G*	1
	N330.6	420.4	10G	0	464.7	15CAAACAA	0	Absent			428.5	12G	0
V134/1st	N134.1	424.1	14G	1	464.7 (10)	15CAAACAA	0	Absent			430.7	13G	1
	N134.2	424.4	14G	1	464.7	15CAAACAA	0	Absent			430.7	13G	1
	N134.3	424.5	14G	1	464.7	15CAAACAA	0	Absent			430.7	13G	1
	N134.4	424.5	14G	1	464.6	15CAAACAA	0	Absent			430.8	13G	1
	N134.5	424.2	14G	1	464.7	15CAAACAA	0	Absent			430.8	13G	1

	N134.6	424.2	14G	1	464.7	15CAAACAA	0	Absent			430.9	13G	1
V134/2nd	N295.1	424.5	14G	1	464.3	15CAAACAA	0	Absent			430.9	13G	1
	N295.2	424.6	14G	1	438.7	11CAAACAA	0	Absent			430.8	13G	1
	N295.3	424.6	14G	1	424.4	9CAAACAA	0	Absent			430.9	13G	1
	N295.4	424.6	14G	1	424.4	9CAAACAA	0	Absent			430.8	13G	1
	N295.5	424.6	14G	1	464.6	15CAAACAA	0	Absent			429.7	13G*	1
	N295.6	424.7	14G	1	424.4	9CAAACAA	0	Absent			429.7	13G	1
V134/3rd	N333.1	424.6	14G	1	464.5 (11)	15CAAACAA	0	Absent			429.7	13G	1
	N333.2	423.6	13G	0	464.7	15CAAACAA	0	Absent			429.8	13G	1
	N333.3	424.6	14G	1	464.6	15CAAACAA	0	Absent			429.7	13G	1
	N333.4	423.6	13G	0	464.7	15CAAACAA	0	Absent			430.9	13G	1
	N333.5	423.6	13G	0	464.7	15CAAACAA	0	Absent			430.9	13G	1
	N333.6	423.6	13G	0	464.7	15CAAACAA	0	Absent			430.8	13G	1
								Absent					
V185/1st	N185.1	421.2 (11)	11G	1	438.6 (11)	11CAAACAA	0	Absent			430.8	13G*	1
	N185.2	421.5 (11)	11G	1	438.7 (11)	11CAAACAA	0	Absent			429.6	13G	1
	N185.3	421.5	11G	1	438.6	11CAAACAA	0	Absent			430.1	13G	1
	N185.4	421.4	11G	1	438.6	11CAAACAA	0	Absent			429.8	13G	1
	N185.5	421.1	11G	1	438.7	11CAAACAA	0	Absent			430.6	13G	1
	N185.6	421.2	11G	1	438.7	11CAAACAA	0	Absent			429.6	12G	0
V185/2nd	N306.1	421.5	11G	1	424.4	9CAAACAA	0	Absent			429.7	13G	1
	N306.2	421.4	11G	1	437.7	11CAAACAA	0	Absent			430.9	13G	1
	N306.3	421.1	11G	1	421.2	9CAAACAA	0	Absent			430.7	13G	1
	N306.4	421.2	11G	1	421.1	9CAAACAA	0	Absent			430.8	13G	1
	N306.5	421.2	11G	1	438.6	11CAAACAA	0	Absent			430.8	13G	1
	N306.6	421.3	11G	1	438.7	11CAAACAA	0	Absent			430.8	13G	1
V185/4th	N456.1	421.1 (11)	11G	1	438.7 (11)	11CAAACAA	0	Absent			428.6	12G	0
	N456.2	421.2 (11)	11G	1	438.7 (11)	11CAAACAA	0	Absent			429.6	12G	0
	N456.3	421.1	11G*	1	438.7	11CAAACAA	0	Absent			431.3	14G	0
	N456.4	421.2	11G	1	438.7	11CAAACAA	0	Absent			428.7	11G	0
	N456.5	420.9	11G	1	438.7	11CAAACAA	0	Absent			427.1	10G	0
	N456.6	421.3	11G	1	438.7	11CAAACAA	0	Absent			428.9	12G	0

V113/1st	N113.1	424.0	14G	1	464.2	15CAAACAA*	0	Absent			428.5	12G	0
	N113.2	424.1	14G	1	464.3	15CAAACAA	0	Absent			429.9	13G	1
	N113.3	424.1	14G	1	464.7	15CAAACAA	0	Absent			429.5	13G	1
	N113.4	424.1	14G	1	464.7	15CAAACAA	0	Absent			429.7	13G	1
	N113.5	424.1	14G	1	464.7	15CAAACAA	0	Absent			429.7	13G	1
	N113.6	424.2>423.2 {1.2}	14G	1	464.7	15CAAACAA	0	Absent			430.0	13G	1
V113/2nd	N281.1	424.1	14G	1	464.6	15CAAACAA	0	Absent			429.4	13G*	1
	N281.2	424.2	14G	1	464.6	15CAAACAA	0	Absent			429.8>428.8 {1.2}	13G	1
	N281.3	424.1	14G	1	464.7	15CAAACAA	0	Absent			429.9	13G	1
	N281.4	423.1	13G	0	464.5	15CAAACAA	0	Absent			430.0	13G	1
	N281.5	423.9	14G	1	464.6	15CAAACAA	0	Absent			429.6	13G	1
	N281.6	423.9	14G	1	464.3	15CAAACAA	0	Absent			430.1	13G	1
V115/1st	N115.1	424.1	14G	1	464.7	15CAAACAA	0	Absent			429.7	13G	1
	N115.2	424.2>423.2 {1.2}	14G	1	464.7	15CAAACAA	0	Absent			430.2	13G	1
	N115.3	424.2	14G	1	464.7	15CAAACAA	0	Absent			429.7	13G	1
	N115.4	424.3	14G	1	464.7	15CAAACAA	0	Absent			430.0	13G	1
	N115.5	424.0>423.0 {1.1]	14G	1	464.7	15CAAACAA	0	Absent			429.9>428.9 {1.2}	13G	1
	N115.6	424.1>423.1 {1.2}	14G	1	464.6	15CAAACAA	0	Absent			430.2	13G	1
V115/2nd	N282.1	423.9	14G	1	464.3	15CAAACAA	0	Absent			429.6	13G	1
	N282.2	424.0>422.9 {1.2}	14G	1	464.2	15CAAACAA	0	Absent			430.1	13G	1
	N282.3	424.0	14G	1	464.3	15CAAACAA	0	Absent			430.0	13G	1
	N282.4	424.1	14G	1	464.4	15CAAACAA	0	Absent			429.6	13G	1
	N282.5	424.2	14G	1	464.5	15CAAACAA	0	Absent			429.7	13G	1
	N282.6	424.2	14G	1	464.4	15CAAACAA	0	Absent			430.0	13G	1
**cc32**													
V176/1st	N176.1	429.8	16G	0	533.6 (14)	24CAAACAA	1	Absent			354.5	15G	0
	N176.2	429.9	16G	0	511.1	21CAAACAA	1	Absent			354.5	15G	0
	N176.3	429.9	16G	0	511.2	21CAAACAA	1	Absent			354.5	15G	0
	N176.4	429.9	16G	0	526.0	23CAAACAA	0	Absent			354.6	15G	0
	N176.5	429.9	16G	0	525.7	23CAAACAA	0	Absent			354.5	15G	0

	N176.6	429.9	16G	0	511.3	21CAAACAA	1	Absent			354.5	15G	0
V176/2nd	N318.1	430.1	16G	0	533.7	24CAAACAA	1	Absent			355.4	16G	1
	N318.2	430.1	16G	0	525.9	23CAAACAA	0	Absent			355.5	16G	1
	N318.3	430.2	16G	0	533.3	24CAAACAA	1	Absent			355.5	16G	1
	N318.4	430.2	16G	0	533.6	24CAAACAA	1	Absent			355.4	16G	1
	N318.5	430.3	16G	0	526.0	23CAAACAA	0	Absent			355.6	16G	1
	N318.6	430.1	16G	0	525.9	23CAAACAA	0	Absent			355.4	16G	1
V176/3rd	N399.1	429.9	16G	0	541.1	25CAAACAA	0	Absent			354.6	15G	0
	N399.2	428.9	15G	0	533.5	24CAAACAA	1	Absent			354.5	15G	0
	N399.3	428.9	15G	0	449.8	12CAAACAA	1	Absent			354.5	15G	0
	N399.4	428.9	15G	0	540.5	25CAAACAA	0	Absent			354.6	15G	0
	N399.5	429.9	16G	0	533.1	24CAAACAA	1	Absent			354.5	15G	0
	N399.6	429.9	16G	0	541.0	25CAAACAA	0	Absent			354.5	15G	0
V176/4th	N408.1	429.0	15G	0	533.6 (12)	24CAAACAA	1	Absent			353.5	14G	0
	N408.2	429.2	15G	0	540.7	25CAAACAA	0	Absent			353.5	14G	0
	N408.3	429.1	15G	0	533.4	24CAAACAA	1	Absent			353.7	14G	0
	N408.4	429.2	15G	0	533.8	24CAAACAA	1	Absent			353.5	14G	0
	N408.5	429.2	15G	0	540.5	25CAAACAA	0	Absent			353.5	14G	0
	N408.6	429.1	15G	0	533.4	24CAAACAA	1	Absent			353.4	14G	0

[1]Fragments sizes were determined using either a GS600LIZ (black type) or GS500LIZ (blue type) size standard; numbers in curly brackets indicate the ratio between the PCR fragment sizes where two obvious peaks were observed (where double peaks were observed, a ratio of >1.2 was utilised as a cut-off with the highest peak height being used to determine repeat number, if the ratio was <1.2 then the larger peak in bp was utilised to determine repeat number except in the cases of *pglI* and *pglH* where sequencing indicated that the these double peaks followed the following rules 191/192, 17; 190/191, 16; 189/190, 15; 188/189, 14; 187/188, 14; 186/187, 13; 185/186, 12). [2]an asterisk indicates that the PCR fragment was sequenced by dideoxy sequencing and the repeat number determined by visual inspection of the trace file. [3]Expression states, 1=1, 0=0 (all genes are subject to translational phase variation).

Supplementary Data Table III. Repeat numbers and expression states for the phase-variable restriction-modification genes

Volunteer /time pt	Isolate	Phase Variable Gene					
		modA			*modB*		
		Size[1]	Tract[2]	Exp. State[3]	Size[1]	Tract[2]	Exp. State[3]
cc174							
V43/1st	N43.1.1	198.2	3AGCC	0	439.0	16GCCAA	1
	N43.1.2	198.2	3AGCC*	0	439.1	16GCCAA	1
	N43.1.3	198.3	3AGCC	0	439.1	16GCCAA	1
	N43.1.4	198.3	3AGCC	0	438.7	16GCCAA	1
	N43.1.5	198.3	3AGCC	0	438.7	16GCCAA	1
	N43.1.6	198.2	3AGCC	0	457.7	20GCCAA	0
V43/2nd	N241.1	198.4	3AGCC	0	443.7	17GCCAA	0
	N241.2	198.3	3AGCC	0	443.6	17GCCAA*	0
V43/3rd	N349.1	199.7	3AGCC*	0	438.7	16GCCAA	1
	N349.2	198.3	3AGCC	0	438.8	16GCCAA	1
	N349.3	198.3	3AGCC	0	438.7	16GCCAA	1
	N349.4	198.4	3AGCC	0	438.9	16GCCAA	1
	N349.5	198.3	3AGCC	0	439.4	16GCCAA	1
	N349.6	198.2	3AGCC	0	438.6	16GCCAA	1
V43/4th	Cleared						
V51/1st	N51.1	198.3	3AGCC	0	443.8	17GCCAA	0
	N51.2	198.3	3AGCC	0	443.6	17GCCAA	0
	N51.3	198.3	3AGCC	0	443.8	17GCCAA	0
	N51.4	198.5	3AGCC	0	444.0	17GCCAA	0
	N51.5	198.3	3AGCC	0	443.7	17GCCAA	0
	N51.6	198.3	3AGCC	0	444.0	17GCCAA	0
V51/2nd	N236.1	198.3	3AGCC	0	443.8	17GCCAA	0
	N236.2	198.4	3AGCC	0	438.2	16GCCAA	1
	N236.3	198.4	3AGCC	0	438.8	16GCCAA	1
	N236.4	198.3	3AGCC	0	438.8	16GCCAA	1
	N236.5	198.3	3AGCC	0	444.0	17GCCAA	0
	N236.6	198.3	3AGCC	0	444.0	17GCCAA	0
V51/3rd	N354.1	198.3	3AGCC	0	444.0	17GCCAA	0
	N354.2	198.2	3AGCC	0	444,0	17GCCAA	0
	N354.3	198.2	3AGCC	0	444.1	17GCCAA	0
	N354.4	198.2	3AGCC	0	444.1	17GCCAA	0

	N354.5	198.3	3AGCC	0	444.1	17GCCAA	0
	N354.6	198.2	3AGCC	0	444.0	17GCCAA	0
V51/4th	N424.1	198.3	3AGCC	0	444.1	17GCCAA	0
	N424.2	198.4	3AGCC	0	444.0	17GCCAA	0
	N424.3	198.4	3AGCC	0	444.0	17GCCAA	0
	N424.4	198.4	3AGCC	0	458.0	20GCCAA	0
	N424.5	198.3	3AGCC	0	444.1	17GCCAA	0
	N424.6	198.2	3AGCC	0	448.9	18GCCAA	0
V52/1st	N52.1	198.3	3AGCC	0	444.0	17GCCAA	0
	N52.2	198.5	3AGCC	0	444.0	17GCCAA	0
	N52.3	198.2	3AGCC	0	444.1	17GCCAA	0
	N52.4	198.2	3AGCC	0	444.1	17GCCAA	0
	N52.5	198.3	3AGCC	0	444.1	17GCCAA	0
	N52.6	198.3	3AGCC	0	444.1	17GCCAA	0
V52/2nd	N238.1	198.3	3AGCC	0	444.1	17GCCAA	0
	N238.2	198.2	3AGCC	0	444.1	17GCCAA	0
	N238.3	198.4	3AGCC	0	448.9	18GCCAA	0
	N238.4	198.3	3AGCC	0	444.1	17GCCAA	0
	N238.5	198.3	3AGCC	0	444.0	17GCCAA	0
	N238.6	198.2	3AGCC	0	448.9	18GCCAA	0
V52/3rd	N342.1	198.2	3AGCC	0	438.8	16GCCAA	1
V52/4th	Cleared						
V54/1st	N54.1.1	198.3	3AGCC	0	439.1 (16)	16GCCAA	1
	N54.1.2	198.3	3AGCC	0	439.1	16GCCAA	1
	N54.1.3	198.2	3AGCC	0	439.2	16GCCAA	1
	N54.1.4	198.3	3AGCC	0	439.1	16GCCAA	1
	N54.1.5	198.2	3AGCC	0	439.2	16GCCAA	1
	N54.1.6	198.3	3AGCC	0	439.1	16GCCAA	1
V54/2nd	N237.1	198.3	3AGCC	0	439.2	16GCCAA	1
	N237.2	198.4	3AGCC	0	439.1	16GCCAA	1
	N237.3	198.5	3AGCC	0	439.1	16GCCAA	1
	N237.4	198.3	3AGCC	0	439.0	16GCCAA	1
	N237.5	198.3	3AGCC	0	439.0	16GCCAA	1
	N237.6	198.3	3AGCC	0	438.5*	16GCCAA	1
V54/3rd	N343.1	198.2	3AGCC	0	439.2 (16)	16GCCAA	1

	N343.2	198.3	3AGCC	0	439.1	16GCCAA	1
	N343.3	198.4	3AGCC	0	439.2	16GCCAA	1
	N343.4	198.3	3AGCC	0	439.1	16GCCAA	1
	N343.5	198.2	3AGCC	0	439.1	16GCCAA	1
	N343.6	198.4	3AGCC	0	444.1	17GCCAA	0
V54/4th	Replaced						
V58/1st	N58.1	198.2	3AGCC	0	444.0 (17)	17GCCAA	0
	N58.2	198.4	3AGCC	0	444.1	17GCCAA	0
	N58.3	198.3	3AGCC	0	444.0	17GCCAA	0
	N58.4	198.4	3AGCC	0	444.0	17GCCAA	0
	N58.5	198.3	3AGCC	0	443.9	17GCCAA	0
	N58.6	198.4	3AGCC	0	444.0	17GCCAA	0
V58/2nd	N240.1	198.4	3AGCC	0	453.5	19GCCAA	1
	N240.2	198.3	3AGCC	0	471.7	23GCCAA	0
V58/3rd	Cleared						
V58/4th	N429.1	198.3	3AGCC	0	394.4 (7)	8GCCAA	0
	N429.2	198.3	3AGCC	0	394.8	8GCCAA	0
	N429.3	198.2	3AGCC	0	467.1	22GCCAA	1
	N429.4	198.4	3AGCC	0	471.8	23GCCAA	0
	N429.5	198.3	3AGCC	0	394.5	8GCCAA	0
	N429.6	198.4	3AGCC	0	467.1	22GCCAA	1
V59/1st	N59.1.1	198.4	3AGCC	0	443.7 (17)	17GCCAA	0
	N59.1.2	198.4	3AGCC	0	443.1	17GCCAA	0
	N59.1.3	198.3	3AGCC	0	444.0	17GCCAA	0
	N59.1.4	198.4	3AGCC	0	444.0	17GCCAA	0
	N59.1.5	198.3	3AGCC	0	444.0	17GCCAA	0
	N59.1.6	198.3 (3)	3AGCC	0	444.0	17GCCAA	0
V59/2nd	N253.1	198.3	3AGCC	0	443.9 (17)	17GCCAA	0
	N253.2	198.4	3AGCC	0	444.0 (17)	17GCCAA	0
	N253.3	198.2	3AGCC	0	443.9	17GCCAA	0
	N253.4	198.4	3AGCC	0	444.0 (16)	17GCCAA	0
	N253.5	198.3	3AGCC	0	433.9 (15)	15GCCAA	0
	N253.6	198.4	3AGCC	0	443.9 (17)	17GCCAA	0
V59/3rd	N352.1	198.3	3AGCC	0	438.5	16GCCAA	1
	N352.2	198.5	3AGCC	0	438.1	16GCCAA	1

	N352.3	198.3	3AGCC	0	448.1 (18)	18GCCAA	0
	N352.4	198.5	3AGCC	0	447.7	18GCCAA	0
	N352.5	198.3	3AGCC	0	442.8	17GCCAA	0
	N352.6	198.4	3AGCC	0	443.0	17GCCAA	0
V59/4th	N438.1	198.3	3AGCC	0	462.7	21GCCAA	0
	N438.2	198.5	3AGCC	0	462.4	21GCCAA	0
	N438.3	198.5	3AGCC	0	443.1	17GCCAA	0
	N438.4	198.5	3AGCC	0	447.8	18GCCAA	0
	N438.5	198.3	3AGCC	0	462.6 (16)	21GCCAA	0
	N438.6	198.5	3AGCC	0	457.7	20GCCAA	0
V88/1st	N88.1.1	198.3	3AGCC	0	525.9	34GCCAA*	1
	N88.1.2	198.4	3AGCC	0	525.5	34GCCAA	1
	N88.1.3	198.3	3AGCC	0	525.8	34GCCAA	1
	N88.1.4	198.5	3AGCC	0	525.5	34GCCAA	1
	N88.1.5	198.3	3AGCC	0	525.7	34GCCAA	1
	N88.1.6	198.5	3AGCC	0	525.6	34GCCAA	1
V88/2nd	N272.1	198.3	3AGCC	0	525.8	34GCCAA	1
	N272.2	198.5	3AGCC	0	530.4	35GCCAA*	0
	N272.3	198.4	3AGCC	0	525.8	34GCCAA	1
	N272.4	198.5	3AGCC	0	530.3	35GCCAA	0
	N272.5	198.3	3AGCC	0	525.8	34GCCAA	1
	N272.6	198.5	3AGCC	0	525.6	34GCCAA	1
V88/3rd	N369.1	198.3	3AGCC	0	501.5	29GCCAA	0
	N369.2	198.4	3AGCC	0	501.2	29GCCAA	0
	N369.3	198.3	3AGCC	0	530.4	35GCCAA	0
	N369.4	198.3	3AGCC	0	530.3	35GCCAA	0
	N369.5	198.3	3AGCC	0	501.2	29GCCAA	0
	N369.6	198.5	3AGCC	0	501.2	29GCCAA	0
V88/4th	N449.1	198.3	3AGCC	0	539.9	37GCCAA	1
	N449.2	198.5	3AGCC	0	539.9	37GCCAA	1
	N449.3	198.4	3AGCC	0	539.9	37GCCAA	1
	N449.4	198.4	3AGCC	0	540>544.9 {1.1}	37GCCAA	1
	N449.5	198.4	3AGCC	0	540.0	37GCCAA	1
	N449.6	198.5	3AGCC	0	540.0	37GCCAA	1
V138/1st	N138.1	198.3	3AGCC	0	443.4	17GCCAA*	0

	N138.2	198.5	3AGCC	0	443.0	17GCCAA	0
	N138.3	198.3	3AGCC	0	443.1	17GCCAA	0
	N138.4	198.5	3AGCC	0	442.6	17GCCAA	0
	N138.5	198.3	3AGCC	0	442.7	17GCCAA	0
	N138.6	198.5	3AGCC	0	443.0	17GCCAA	0
V138/2nd	N288.1	198.3	3AGCC	0	438.1	16GCCAA	1
	N288.2	198.5	3AGCC	0	442.9	17GCCAA	0
	N288.3	198.4	3AGCC	0	443.0	17GCCAA	0
	N288.4	198.4	3AGCC	0	443.0	17GCCAA	0
	N288.5	198.2	3AGCC	0	472.3	23GCCAA	0
	N288.6	198.5	3AGCC	0	447.8	18GCCAA	0
V138/3rd	N331.1	198.4	3AGCC	0	452.8 (16)	19GCCAA	1
	N331.2	198.5	3AGCC	0	452.7	19GCCAA	1
	N331.3	198.3	3AGCC	0	452.9	19GCCAA	1
	N331.4	198.5	3AGCC	0	452.6	19GCCAA	1
	N331.5	198.3	3AGCC	0	452.5	19GCCAA	1
	N331.6	198.5	3AGCC	0	452.6	19GCCAA	1
V138/4th	Replaced						
**cc167**							
V124/1st	N124.1	240.7 (13)	13AGCC	1	Absent		
	N124.2	241.3	13AGCC	1	Absent		
	N124.3	240.8	13AGCC	1	Absent		
	N124.4	241.2	13AGCC	1	Absent		
	N124.5	236.7	12AGCC	0	Absent		
	N124.6	241.1	13AGCC	1	Absent		
V124/2nd	N290.1	240.7	13AGCC	1	Absent		
	N290.2	241.2	13AGCC	1	Absent		
	N290.3	240.7	13AGCC	1	Absent		
	N290.4	241.1	13AGCC	1	Absent		
	N290.5	240.8	13AGCC	1	Absent		
	N290.6	236.8	13AGCC*	1	Absent		
V124/3rd	N336.1	240.9 (13)	13AGCC	1	Absent		
	N336.2	241.0	14AGCC	0	Absent		
	N336.3	240.6	13AGCC	1	Absent		
	N336.4	240.6	13AGCC	1	Absent		
	N336.5	240.6	13AGCC	1	Absent		

	N336.6	240.6	13AGCC	1	Absent		
					Absent		
V64/1st	N64.1	240.5 (13)	13AGCC	1	Absent		
	N64.2	240.7	13AGCC	1	Absent		
	N64.3	240.7	13AGCC	1	Absent		
	N64.4	240.7	13AGCC	1	Absent		
	N64.5	240.6	13AGCC	1	Absent		
	N64.6	240.8	13AGCC	1	Absent		
V64/2nd	N257.1	240.8	13AGCC	1	Absent		
	N257.2	240.6	13AGCC	1	Absent		
	N257.3	240.6	13AGCC	1	Absent		
	N257.4	244.8	14AGCC	0	Absent		
	N257.5	240.6	13AGCC	1	Absent		
	N257.6	244.8	14AGCC	0	Absent		
V64/3rd	N348.1	240.5 (13)	13AGCC	1	Absent		
	N348.2	240.6	13AGCC	1	Absent		
	N348.3	244.7	14AGCC	0	Absent		
	N348.4	244.9	14AGCC	0	Absent		
	N348.5	236.5	12AGCC	0	Absent		
	N348.6	240.8	13AGCC	1	Absent		
					Absent		
V117/1st	N117.1	240.8 (13)	13AGCC	1	Absent		
	N117.2	244.8	14AGCC	0	Absent		
V117/2nd	N284.1	240.6	13AGCC	1	Absent		
	N284.2	240.6	13AGCC	1	Absent		
	N284.3	244.7	14AGCC	0	Absent		
	N284.4	240.6	13AGCC	1	Absent		
	N284.5	240.6	13AGCC	1	Absent		
	N284.6	240.6	13AGCC	1	Absent		
V117/3rd	N332.1	240.5	13AGCC	1	Absent		
	N332.2	240.6	13AGCC	1	Absent		
	N332.3	240.6	13AGCC	1	Absent		
	N332.4	240.7	13AGCC	1	Absent		
	N332.5	245.0>240.7 (1)	14AGCC	0	Absent		
	N332.6	244.9	14AGCC	0	Absent		
V117/4th	N417.1	240.6>236.4 (1) (13)	13AGCC	1	Absent		
	N417.2	240.5>236.3 (1.1)	13AGCC	1	Absent		

	N417.3	244.7>240.5 (1)	14AGCC	0	Absent		
	N417.4	240.6	13AGCC	1	Absent		
	N417.5	244.8	14AGCC	0	Absent		
	N417.6	244.8	14AGCC	0	Absent		
					Absent		
**cc23**							
V69/1st	N1-carrier				Absent		
V69/2nd	N258.1	256.8	17AGCC	0	Absent		
	N258.2	253.1 (16)	16AGCC	1	Absent		
	N258.3	249.8 (16)	16AGCC*	1	Absent		
	N258.4	253.2 (16)	16AGCC	1	Absent		
	N258.5	257.1 (17)	17AGCC	0	Absent		
	N258.6	253.2 (16)	16AGCC	1	Absent		
V69/3rd	No sample				Absent		
V69/4th	N431.1	257.1 (17)	17AGCC	0	Absent		
	N431.2	261.0 (18)	18AGCC	0	Absent		
	N431.3	260.9 (18)	18AGCC	0	Absent		
	N431.4	261.9 (19)	19AGCC	1	Absent		
	N431.5	256.9 (17)	17AGCC	0	Absent		
	N431.6	260.8 (18)	18AGCC	1	Absent		
V93/1st	N1-carrier				Absent		
V93/2nd	N264.1	249.0 (15)	15AGCC	0	Absent		
	N264.2	249.1	15AGCC	0	Absent		
	N264.3	253.1	16AGCC	1	Absent		
	N264.4	249.2	15AGCC	0	Absent		
	N264.5	249.3	15AGCC	0	Absent		
	N264.6	249.2	15AGCC	0	Absent		
V93/3rd	N359.1	257.1	17AGCC	0	Absent		
	N359.2	253.9	16AGCC	1	Absent		
	N359.3	257.0	17AGCC	0	Absent		
	N359.4	257.0	17AGCC	0	Absent		
	N359.5	256.9	17AGCC	0	Absent		
	N359.6	256.9	17AGCC	0	Absent		
V96/1st	N1-carrier				Absent		
V96/2nd	N259.1	260.9	18AGCC	0	Absent		

	N259.2	260.8	18AGCC	0	Absent		
	N259.3	260.9	18AGCC	0	Absent		
	N259.4	261.0	18AGCC	0	Absent		
	N259.5	261.0	18AGCC	0	Absent		
	N259.6	257.1	17AGCC	0	Absent		
V96/3rd	N370.1	264.9	19AGCC	1	Absent		
	N370.2	264.9	19AGCC	1	Absent		
	N370.3	260.9	18AGCC	0	Absent		
	N370.4	260.9	18AGCC	0	Absent		
	N370.5	260.9	18AGCC	0	Absent		
	N370.6	264.8	19AGCC	0	Absent		
V96/4th	N445.1	264.8	19AGCC	0	Absent		
	N445.2	264.8	19AGCC	0	Absent		
	N445.3	264.8	19AGCC	0	Absent		
	N445.4	264.9	19AGCC	0	Absent		
	N445.5	264.9	19AGCC	0	Absent		
	N445.6	265.0	19AGCC	0	Absent		
V222/1st	N222.1	224.0 (9)	9AGCC*	0	Absent		
	N222.2	215.5	7AGCC	1	Absent		
	N222.3	215.5	7AGCC	1	Absent		
	N222.4	215.4	7AGCC	1	Absent		
	N222.5	219.6	8AGCC	0	Absent		
	N222.6	219.7	8AGCC	0	Absent		
V222/2nd	N309.1	215.4	7AGCC	1	Absent		
	N309.2	215.4	7AGCC	1	Absent		
	N309.3	215.3	7AGCC	1	Absent		
	N309.4	215.5	7AGCC	1	Absent		
	N309.5	212.3	7AGCC	1	Absent		
	N309.6	212.4	7AGCC	1	Absent		
V222/3rd	N391.1	216.5	8AGCC	0	Absent		
	N391.2	216.7	8AGCC	0	Absent		
	N391.3	216.4	8AGCC	0	Absent		
	N391.4	216.5	8AGCC	0	Absent		
	N391.5	216.4	8AGCC	0	Absent		
	N391.6	220.6	9AGCC	0	Absent		
V222/4th	N459.1	216.6 (8)	8AGCC	0	Absent		

	N459.2	216.8	8AGCC	0	Absent		
	N459.3	216.6	8AGCC	0	Absent		
	N459.4	216.6	8AGCC	0	Absent		
	N459.5	216.5	8AGCC	0	Absent		
	N459.6	216.6	8AGCC	0	Absent		
**cc60**							
V114/1st	N114.1	257.1 (17)	17AGCC	0	419.1 (12)	12GCCAA	0
	N114.2	257.0	17AGCC	0	419.4	12GCCAA	0
	N114.3	257.1	17AGCC	0	419.2	12GCCAA	0
	N114.4	257.0	17AGCC	0	419.3	12GCCAA	0
	N114.5	257.0	17AGCC	0	419.3	12GCCAA	0
	N114.6	256.9	17AGCC	0	414.4	11GCCAA	0
V114/2nd	N283.1	249.1	15AGCC	0	428.9	14GCCAA	0
	N283.2	260.8	18AGCC	0	429.1	14GCCAA	0
	N283.3	257.1	17AGCC	0	428.9	14GCCAA	0
	N283.4	249.1	15AGCC	0	428.8	14GCCAA	0
	N283.5	261.0	18AGCC	0	23.8	13GCCAA	1
	N283.6	257.1	17AGCC	0	428.9	14GCCAA	0
V114/3rd	N330.1	257.0 (17)	17AGCC	0	424.1 (13)	13GCCAA	1
	N330.2	256.9	17AGCC	0	424.2	13GCCAA	1
	N330.3	257.0	17AGCC	0	424.2	13GCCAA	1
	N330.4	256.9	17AGCC	0	424.3	13GCCAA	1
	N330.5	257.0	17AGCC	0	424.1	13GCCAA	1
	N330.6	256.9	17AGCC	0	424.1	13GCCAA	1
V134/1st	N134.1	257.0 (17)	17AGCC	0	424.0 (13)	13GCCAA	1
	N134.2	256.9	17AGCC	0	419.3	12GCCAA	0
	N134.3	257.1	17AGCC	0	419.2	12GCCAA	0
	N134.4	257.0	17AGCC	0	419.3	12GCCAA	0
	N134.5	257.1	17AGCC	0	418.9	12GCCAA	0
	N134.6	253.1	16AGCC	1	419.1	12GCCAA	0
V134/2nd	N295.1	257.0	17AGCC	0	419.3	12GCCAA	0
	N295.2	257.0	17AGCC	0	419.2	12GCCAA	0
	N295.3	249.2	15AGCC	0	419.2	12GCCAA	0
	N295.4	256.8	17AGCC	0	419.3	12GCCAA	0
	N295.5	249.2	15AGCC	0	419.3	12GCCAA	0

	N295.6	256.8	17AGCC	0	419.4	12GCCAA	0
V134/3rd	N333.1	257.0 (17)	17AGCC	0	419.2 (12)	12GCCAA	0
	N333.2	256.9	17AGCC	0	419.3	12GCCAA	0
	N333.3	253.2	16AGCC	1	419.2	12GCCAA	0
	N333.4	256.9	17AGCC	0	414.4	11GCCAA	0
	N333.5	257.2	17AGCC	0	419.2	12GCCAA	0
	N333.6	257.0	17AGCC	0	419.3	12GCCAA	0
V185/1st	N185.1	253.3 (16)	16AGCC	1	404.4 (9)	9GCCAA	0
	N185.2	253.2 (16)	16AGCC	1	409.4 (10)	10GCCAA	1
	N185.3	253.2	16AGCC	1	404.4	9GCCAA	0
	N185.4	257.0	17AGCC	0	404.5	9GCCAA	0
	N185.5	249.3	15AGCC	0	404.4	9GCCAA	0
	N185.6	253.1	16AGCC	1	404.5	9GCCAA	0
V185/2nd	N306.1	249.2	15AGCC	0	404.4	9GCCAA	0
	N306.2	244.9	14AGCC	0	404.5	9GCCAA	0
	N306.3	249.3	15AGCC	0	404.4	9GCCAA	0
	N306.4	249.2	15AGCC	0	404.5	9GCCAA	0
	N306.5	249.4	15AGCC	0	404.3	9GCCAA	0
	N306.6	249.3	15AGCC	0	404.4	9GCCAA	0
V185/4th	N456.1	245.2 (14)	14AGCC	0	404.3 (9)	9GCCAA	0
	N456.2	245.1 (14)	14AGCC	0	404.3 (9)	9GCCAA	0
	N456.3	240.8	13AGCC	1	409.3	10GCCAA	1
	N456.4	257.0	17AGCC	0	404.5	9GCCAA	0
	N456.5	253.2	16AGCC	1	404.5	9GCCAA	0
	N456.6	249.2	15AGCC	0	404.5	9GCCAA	0
V113/1st	N113.1	257.1	17AGCC	0	419.3	12GCCAA	0
	N113.2	249.1	15AGCC	0	404.5	9GCCAA	0
	N113.3	257.0	17AGCC	0	419.3	12GCCAA	0
	N113.4	256.9	17AGCC	0	419.3	12GCCAA	0
	N113.5	257.2	17AGCC	0	419.2	12GCCAA	0
	N113.6	257.0	17AGCC	0	419.3	12GCCAA	0
V113/2nd	N281.1	257.1	17AGCC	0	419.2	12GCCAA	0
	N281.2	257.1	17AGCC	0	419.3	12GCCAA	0
	N281.3	257.0	17AGCC	0	419.0	12GCCAA	0
	N281.4	256.9	17AGCC	0	419.2	12GCCAA	0

	N281.5	257.0	17AGCC	0	419.3	12GCCAA	0
	N281.6	256.9	17AGCC	0	419.2	12GCCAA	0
V115/1st	N115.1	257.0	17AGCC	0	419.2	12GCCAA	0
	N115.2	253.6	17AGCC	0	418.6	12GCCAA*	0
	N115.3	257.1	17AGCC	0	419.2	12GCCAA	0
	N115.4	257.0	17AGCC	0	419.3	12GCCAA	0
	N115.5	257.2	17AGCC	0	419.3	12GCCAA	0
	N115.6	257.1	17AGCC	0	419.3	12GCCAA	0
V115/2nd	N282.1	257.2	17AGCC	0	414.3	11GCCAA	0
	N282.2	228.1	10AGCC	1	419.2	12GCCAA	0
	N282.3	257.1	17AGCC	0	419.3	12GCCAA	0
	N282.4	257.0	17AGCC	0	414.4	11GCCAA	0
	N282.5	257.1	17AGCC	0	419.2	12GCCAA	0
	N282.6	257.0	17AGCC	0	414.3	11GCCAA	0
**cc32**							
V176/1st	N176.1	266.1	20AGCC	0	458.0	19CCCAA	0
	N176.2	266.1	20AGCC	0	458.0	19CCCAA	0
	N176.3	266.2	20AGCC	0	458.0	19CCCAA	0
	N176.4	266.1	20AGCC	0	458.0	19CCCAA	0
	N176.5	202.6	4AGCC	1	457.9	19CCCAA	0
	N176.6	266.3	20AGCC	0	458.0	19CCCAA	0
V176/2nd	N318.1	266.2	20AGCC	0	457.9	19CCCAA	0
	N318.2	266.2	20AGCC	0	458.0	19CCCAA	0
	N318.3	266.3	20AGCC	0	457.9	19CCCAA	0
	N318.4	266.1	20AGCC	0	458.0	19CCCAA	0
	N318.5	266.1	20AGCC	0	458.0	19CCCAA	0
	N318.6	266.4	20AGCC	0	458.0	19CCCAA	0
V176/3rd	N399.1	266.4	20AGCC	0	458.1	19CCCAA	0
	N399.2	266.3	20AGCC	0	458.0	19CCCAA	0
	N399.3	266.4	20AGCC	0	457.9	19CCCAA	0
	N399.4	266.4	20AGCC	0	458.0	19CCCAA	0
	N399.5	266.3	20AGCC	0	457.9	19CCCAA	0
	N399.6	266.2	20AGCC	0	462.5	20CCCAA*	0
V176/4th	N408.1	266.2	20AGCC	0	457.9	19CCCAA	0
	N408.2	266.3	20AGCC	0	457.7	19CCCAA	0

	N408.3	266.0	20AGCC	0	457.9	19CCCAA	0
	N408.4	266.2	20AGCC	0	452.8	18CCCAA	0
	N408.6	266.3	20AGCC	0	457.6	19CCCAA	0

[1]Fragments sizes were determined using either a GS600LIZ (black type) or GS500LIZ (blue type) size standard; numbers in curly brackets indicate the ratio between the PCR fragment sizes where two obvious peaks were observed. [2]an asterisk indicates that the PCR fragment was sequenced by dideoxy sequencing and the repeat number determined by visual inspection of the trace file. [3]Expression states, 1=1, 0=0 (all genes are subject to translational phase variation).

Supplementary Data Table IV. Repeat numbers and expression states for the phase-variable *opaA* and *opaB* genes

**Carrier/ time point**	**Isolate**	***opaA* (5'CTCTT)**					***opaB* (5'CTCTT)**				
		**Size[1]**	**C tract[2]**	**A tract[2]**	**Repeat No.[3]**	**Exp. State[4]**	**Size[1]**	**C tract[2]**	**A tract[2]**	**Repeat No.[3]**	**Exp. State[4]**
						**cc23**					
V222/ 1st	222.1	482.5	5	6	19*	0	641.5	5	6	9*	1
	222.2	492.6	5	6	21	1	636.6	5	6	8	0
	222.3	487.1	5	6	20*	0	641.0	5	6	9	1
	222.4	487.2	5	6	20	0	640.9	5	6	9	1
	222.5	482.1	5	6	19	0	654.7	5	6	12	1
	222.6	487.1	5	6	20	0	636.0	5	6	8	0
V222/2nd	309.1	492.6	5	6	21	1	636.5	5	6	8	0
	309.2	492.7	5	6	21	1	636.6	5	6	8	0
	309.3	492.6	5	6	21	1	636.5	5	6	8	0
	309.4	497.8	5	6	22	0	636.5	5	6	8	0
	309.5	492.3	5	6	21	1	640.9	5	6	9	1
	309.6	492.7	5	6	21	1	636.6	5	6	8	0
V222/3rd	391.1	487.6	5	6	20	0	636.2	5	6	8	0
	391.2	492.7	5	6	21	1	636.6	5	6	8	0
	391.3	487.6	5	6	20	0	636.5	5	6	8	0
	391.4	487.5	5	6	20	0	636.4	5	6	8	0
	391.5	487.5	5	6	20	0	636.4	5	6	8	0
	391.6	487.5	5	6	20	0	636.4	5	6	8	0
V222/4th	459.1	482.4	5	6	19	0	636.4	5	6	8	0
	459.2	472.3	5	6	17	0	636.8	5	6	8	0
	459.4	476.9	5	6	18	1	636.0	5	6	8	0
	459.4	482.0	5	6	19	0	636.1	5	6	8	0
	459.5	471.7	5	6	17*	0	636.0	5	6	8	0
	459.6	487.3	5	6	20	0	636.9	5	6	8*	0

V96/2nd	259.1	472.0	5	6	17	0	620.7	5	6	5	0
	259.2	477.1	5	6	18	1	620.7	5	6	5	0
	259.3	482.1	5	6	19	0	626.6	5	6	6	1
	259.4	477.1	5	6	18*	1	621.1	5	6	5	0
	259.5	477.2	5	6	18	1	621.1	5	6	5	0
	259.6	482.1	5	6	19	0	626.3	5	6	6	1
V96/3rd	370.1	ND	ND	ND	ND	ND	ND	ND	ND	ND	ND
	370.2	738.6	5	6	18	1	621.4	5	6	5	0
	370.3	487.8	5	6	20*	0	626.7	5	6	6	1
	370.4	748.4	5	6	20*	0	621.4	5	6	5	0
	370.5	738.6	5	6	18	1	621.5	5	6	5	0
	370.6	728.5	5	6	16	0	621.5	5	6	5	0
V96/4th	445.1	728.5	5	6	16	0	625.8	5	6	6	1
	445.2	728.6	5	6	16	0	625.6	5	6	6	1
	445.3	728.5	5	6	16*	0	626.0	5	6	6	1
	445.4	728.5	5	6	16	0	626.1	5	6	6	1
	445.5	728.5	5	6	16	0	626.2	5	6	6	1
	445.6	733.4	5	6	17	0	625.8	5	6	6	1
V69/2nd	258.1	482.0	5	6	19	0	641.7	5	6	9	1
	258.2	482.1	5	6	19	0	641.2	5	6	9	1
	258.3	482.1	5	6	19	0	641.4	5	6	9	1
	258.4	482.6	5	6	19	0	641.8	5	6	9	1
	258.5	482.6	5	6	19	0	641.5	5	6	9	1
	258.6	482.0	5	6	19	0	641.6	5	6	9	1
V69/3rd	431.1	472.2	5	6	17	0	652.1	5	6	11	0
	431.2	472.1	5	6	17	0	657.2	5	6	12	1
	431.3	472.4	5	6	17	0	657.1	5	6	12	1
	431.4	472.4	5	6	17	0	656.7	5	6	12	1
	431.5	472.4	5	6	17	0	672.1	5	6	15	1

	431.6	477.4	5	6	18	1	652.1	5	6	11	0
V93/2nd	264.1	472.5	5	6	17	0	641.6	5	6	9	1
	264.2	472.4	5	6	17	0	641.4	5	6	9	1
	264.3	461.9	5	6	15	1	626.8	5	6	6	1
	264.4	472.4	5	6	17	0	641.9	5	6	9	1
	264.5	472.3	5	6	17	0	641.0	5	6	9	1
	264.6	471.8	5	6	17	0	641.0	5	6	9	1
V93/3rd	359.1	482.7	5	6	19	0	642.0	5	6	9	1
	359.2	482.7	5	6	19	0	641.6	5	6	9	1
	359.3	482.4	5	6	19	0	642.1	5	6	9	1
	359.4	482.6	5	6	19	0	641.2	5	6	9	1
	359.5	487.7	5	6	20	0	641.6	5	6	9	1
	359.6	482.3	5	6	19	0	641.0	5	6	9	1
**cc167**											
V117/1st	117.1	616.5	5	6	4	0	652.8	5	6	12	1
	117.2	616.7	5	6	4*	0	652.8	5	6	12*	1
V117/2nd	284.1	617.0	5	6	4	0	647.9	5	6	11	0
	284.2	617.1	5	6	4	0	658.3	5	6	13	0
	284.3	617.1	5	6	4	0	653.1	5	6	12	1
	284.4	617.0	5	6	4	0	658.1	5	6	13	0
	284.5	616.9	5	6	4	0	652.9	5	6	12	1
	284.6	617.0	5	6	4	0	652.8	5	6	12	1
V117/3rd	332.1	616.4	5	6	4	0	683.2	5	6	18*	1
	332.2	616.6	5	6	4	0	673.2	5	6	16	0
	332.3	616.8	5	6	4	0	673.1	5	6	16	0
	332.4	616.8	5	6	4	0	668.0	5	6	15	1
	332.6	616.5	5	6	4	0	647.0	5	6	11	0
V117/4th	417.1	661.0	5	6	13	0	656.9	5	6	13	0
	417.2	661.8	5	6	13	0	657.6	5	6	13	0

	417.3	652.3	5	6	11	0	653.1	5	6	12	1
	417.4	645.7	5	6	10	0	651.3	5	6	12	1
	417.5	652.2	5	6	11	0	653.1	5	6	12	1
	417.6	652.2	5	6	11	0	653.1	5	6	12	1
V64/1st	64.1	616.6	5	6	4*	0	652.8	5	6	12	1
	64.2	616.7	5	6	4	0	652.7	5	6	12	1
	64.3	616.8	5	6	4	0	652.7	5	6	12	1
	64.4	616.8	5	6	4	0	652.8	5	6	12	1
	64.5	616.4	5	6	4	0	651.7	5	6	12	1
	64.6	616.6	5	6	4	0	652.7	5	6	12	1
V64/2nd	257.1	616.6	5	6	4	0	667.7	5	6	15	1
	257.2	617.0	5	6	4	0	668.3	5	6	15	1
	257.3	617.1	5	6	4	0	643.0	5	6	10	0
	257.4	617.1	5	6	4	0	668.3	5	6	15	1
	257.5	617.0	5	6	4	0	668.3	5	6	15	1
	257.6	616.9	5	6	4	0	668.2	5	6	15	1
V64/3rd	348.1	616.5	5	6	4	0	662.7	5	6	14	0
	348.2	616.7	5	6	4	0	662.8	5	6	14	0
	348.3	616.5	5	6	4	0	662.8	5	6	14	0
	348.4	616.7	5	6	4	0	662.7	5	6	14	0
	348.5	616.5	5	6	4	0	662.7	5	6	14	0
	348.6	616.6	5	6	4	0	662.0	5	6	14	0
V124/1st	124.1	617.0	5	6	4	0	657.1	5	6	12	1
	124.2	617.0	5	6	4*	0	657.3	5	6	12*	1
	124.3	616.3	5	6	4	0	656.0	5	6	12	1
	124.4	616.3	5	6	4	0	655.8	5	6	12	1
	124.5	616.3	5	6	4	0	656.0	5	6	12	1
V124/2nd	290.1	616.4	5	6	4	0	656.8	5	6	12	1

	290.3	616.9	5	6	4	0	657.3	5	6	12	1
	290.4	616.8	5	6	4	0	657.1	5	6	12	1
	290.5	616.5	5	6	4	0	656.8	5	6	12	1
	290.6	616.4	5	6	4	0	656.8	5	6	12	1
V124/3rd	336.1	616.6	5	6	4	0	657.0	5	6	12	1
	336.2	616.7	5	6	4	0	657.1	5	6	12	1
	336.3	617.0	5	6	4	0	657.2	5	6	12	1
	336.4	616.8	5	6	4	0	657.1	5	6	12	1
	336.5	616.7	5	6	4	0	656.9	5	6	12	1
	336.6	616.6	5	6	4	0	656.8	5	6	12	1
**cc60**											
V113/1st	113.1	458.6	5	6	13*	0	700.5	5	6	11*	0
	113.2	433.2	5	6	8	0	700.6	5	6	11	0
	113.3	458.7	5	6	13	0	700.7	5	6	11	0
	113.4	433.2	5	6	8	0	700.5	5	6	11	0
	113.5	443.3	5	6	10	0	700.7	5	6	11	0
	113.6	443.2	5	6	10	0	700.5	5	6	11	0
V113/2nd	281.1	443.6	5	6	10	0	700.9	5	6	11	0
	282.2	443.6	5	6	10	0	701.0	5	6	11	0
	282.3	443.7	5	6	10	0	701.0	5	6	11	0
	282.4	438.6	5	6	9	1	701.0	5	6	11	0
	282.5	443.6	5	6	10	0	700.9	5	6	11	0
	282.6	443.6	5	6	10	0	700.9	5	6	11	0
V114/1st	114.1	448.4	5	6	11	0	705.8	5	6	12	1
	114.2	438.3	5	6	9	1	690.7	5	6	9	1
	114.3	438.3	5	6	9	1	700.6	5	6	11	0
	114.4	438.3	5	6	9	1	700.8	5	6	11	0
	114.5	448.4	5	6	11	0	705.6	5	6	12	1
	114.6	448.4	5	6	11	0	705.6	5	6	12	1

	114.7	438.4	5	6	9	1	705.8	5	6	12	1
	114.8	443.4	5	6	10	0	706.0	5	6	12	1
	114.9	443.4	5	6	10	0	705.8	5	6	12	1
	114.10	443.4	5	6	10	0	705.8	5	6	12	1
	114.11	438.3	5	6	9	1	700.6	5	6	11	0
	114.12	438.4	5	6	9	1	700.8	5	6	11	0
	114.13	438.3	5	6	9	1	705.6	5	6	12	1
	114.14	438.3	5	6	9	1	700.6	5	6	11	0
	114.16	443.7	5	6	10	0	706.1	5	6	12	1
	114.18	443.8	5	6	10	0	706.1	5	6	12	1
V114/2nd	283.1	448.3	5	6	11	0	705.4	5	6	12	1
	283.2	448.8	5	6	11	0	706.2	5	6	12	1
	283.3	448.8	5	6	11	0	706.1	5	6	12	1
	283.4	448.4	5	6	11	0	705.6	5	6	12	1
	283.5	448.7	5	6	11	0	706.0	5	6	12	1
	283.6	448.8	5	6	11	0	706.0	5	6	12	1
V114/3rd	330.1	438.1	5	6	9	1	700.5	5	6	11	0
	330.2	438.2	5	6	9	1	700.4	5	6	11	0
	330.3	438.6	5	6	9	1	700.8	5	6	11	0
	330.4	438.5	5	6	9	1	700.7	5	6	11	0
	330.5	438.3	5	6	9	1	700.5	5	6	11	0
	330.6	438.1	5	6	9	1	700.5	5	6	11	0
	330.7	438.3	5	6	9	1	700.7	5	6	11	0
	330.8	438.4	5	6	9	1	700.8	5	6	11	0
	330.9	438.4	5	6	9	1	700.6	5	6	11	0
	330,10	438.4	5	6	9	1	695.7	5	6	10	0
	330.11	438.4	5	6	9	1	700.8	5	6	11	0
	330.12	438.3	5	6	9	1	700.7	5	6	11	0
	330.15	438.7	5	6	9	1	701.0	5	6	11	0
	330.16	438.7	5	6	9	1	701.1	5	6	11	0

	330.17	438.7	5	6	9	1	701.1	5	6	11	0
	330.18	438.8	5	6	9	1	701.0	5	6	11	0
	330.19	438.7	5	6	9	1	701.1	5	6	11	0
	330,20	438.7	5	6	9	1	701.1	5	6	11	0
V115/1st	115.1	433.2	5	6	8	0	705.6	5	6	12	1
	115.2	433.2	5	6	8	0	690.6	5	6	9	1
	115.3	443.3	5	6	10	0	705.7	5	6	12	1
	115.4	443.4	5	6	10	0	705.6	5	6	12	1
	115.5	443.3	5	6	10	0	700.6	5	6	11	0
	115.6	443.3	5	6	10	0	700.6	5	6	11	0
V115/2nd	282.1	433.3	5	6	8	0	700.5	5	6	11	0
	282.2	443.7	5	6	10	0	690.9	5	6	9	1
	282.3	433.6	5	6	8	0	701.0	5	6	11	0
	282.4	433.6	5	6	8	0	701.1	5	6	11	0
	282.5	433.6	5	6	8	0	701.0	5	6	11	0
	282.6	433.6	5	6	8	0	701.0	5	6	11	0
V134/1st	134.1	433.1	5	6	8	0	705.5	5	6	12	1
	134.2	433.2	5	6	8	0	700.6	5	6	11	0
	134.3	433.2	5	6	8	0	700.6	5	6	11	0
	134.4	433.2	5	6	8	0	700.6	5	6	11	0
	134.6	433.2	5	6	8	0	720.8	5	6	15	1
	134.7	433.2	5	6	8	0	700.7	5	6	11	0
	134.8	433.3	5	6	8	0	700.7	5	6	11	0
	134.9	433.3	5	6	8	0	700.7	5	6	11	0
	134,10	433.4	5	6	8	0	700.7	5	6	11	0
	134.11	433.3	5	6	8	0	700.7	5	6	11	0
	134.12	433.3	5	6	8	0	700.6	5	6	11	0
	134.13	433.3	5	6	8	0	700.7	5	6	11	0
	134.14	433.1	5	6	8	0	700.5	5	6	11	0

	134.16	433.6	5	6	8	0	701.2	5	6	11	0
	134.17	433.6	5	6	8	0	701.1	5	6	11	0
	134.18	443.8	5	6	10	0	706.1	5	6	12	1
V134/2nd	295.1	448.7	5	6	11	0	705.6	5	6	12	1
	295.2	448.8	5	6	11	0	706.0	5	6	12	1
	295.3	433.3	5	6	8	0	705.6	5	6	12	1
	295.4	448.4	5	6	11	0	705.5	5	6	12	1
	295.5	432.9	5	6	8	0	705.0	5	6	12	1
	295.6	433.6	5	6	8	0	720.6	5	6	15	1
V134/3rd	333.1	433.1	5	6	8	0	705.5	5	6	12	1
	333.2	433.1	5	6	8	0	705.5	5	6	12	1
	333.3	433.6	5	6	8	0	706.0	5	6	12	1
	333.4	433.5	5	6	8	0	706.0	5	6	12	1
	333.5	433.1	5	6	8	0	705.4	5	6	12	1
	333.6	433.0	5	6	8	0	705.4	5	6	12	1
	333.7	433.3	5	6	8	0	705.8	5	6	12	1
	333.8	433.4	5	6	8	0	705.9	5	6	12	1
	333.9	433.5	5	6	8	0	705.9	5	6	12	1
	333,10	433.5	5	6	8	0	705.9	5	6	12	1
	333.11	433.4	5	6	8	0	705.8	5	6	12	1
	333.12	433.4	5	6	8	0	705.8	5	6	12	1
	333.13	433.4	5	6	8	0	705.7	5	6	12	1
	333.14	433.2	5	6	8	0	705.6	5	6	12	1
	333.15	433.6	5	6	8	0	706.2	5	6	12	1
	333.16	433.7	5	6	8	0	706.2	5	6	12	1
	333.17	433.7	5	6	8	0	706.1	5	6	12	1
	333.18	433.7	5	6	8	0	706.1	5	6	12	1
	333.19	433.7	5	6	8	0	706.2	5	6	12	1
	333,20	433.7	5	6	8	0	706.2	5	6	12	1
V185/1st	185.1	463.6	5	6	13	0	438.2	5	6	8	0

	185.2	463.7	5	6	13	0	438.2	5	6	8	0
	185.3	463.6	5	6	13*	0	438.2	5	6	8	0
	185.4	463.7	5	6	13	0	438.2	5	6	8	0
	185.5	463.6	5	6	13	0	438.2	5	6	8*	0
	185.6	464.1	5	6	13	0	438.7	5	6	8	0
	185.8	463.7	5	6	13	0	438.2	5	6	8	0
	185.9	463.7	5	6	13	0	438.2	5	6	8	0
	185,10	463.8	5	6	13	0	438.4	5	6	8	0
	185.11	463.7	5	6	13	0	438.3	5	6	8	0
	185.12	463.8	5	6	13	0	438.3	5	6	8	0
	185.13	463.7	5	6	13	0	438.3	5	6	8	0
	185.14	463.7	5	6	13	0	438.2	5	6	8	0
	185.15	463.6	5	6	13	0	438.1	5	6	8	0
	185.16	464.0	5	6	13	0	438.6	5	6	8	0
	185.17	464.1	5	6	13	0	438.7	5	6	8	0
	185.18	464.1	5	6	13	0	438.7	5	6	8	0
	185.19	464.2	5	6	13	0	438.7	5	6	8	0
	185,20	464.1	5	6	13	0	438.7	5	6	8	0
V185/2nd	306.1	463.5	5	6	13	0	438.1	5	6	8	0
	306.2	473.8	5	6	15	1	438.2	5	6	8	0
	306.3	463.7	5	6	13	0	438.2	5	6	8	0
	306.4	463.7	5	6	13	0	438.2	5	6	8	0
	306.5	463.6	5	6	13	0	438.1	5	6	8	0
	306.6	463.6	5	6	13	0	438.2	5	6	8	0
V185/4th	456.1	453.6	5	6	11	0	438.4	5	6	8	0
	456.2	453.7	5	6	11	0	438.5	5	6	8	0
	456.3	458.8	5	6	12	1	438.5	5	6	8	0
	456.4	458.9	5	6	12	1	438.5	5	6	8	0
	456.5	453.7	5	6	11	0	438.5	5	6	8	0
	456.6	453.7	5	6	11	0	438.4	5	6	8	0
	456.7	453.9	5	6	11	0	438.3	5	6	8	0

	456.8	458.4	5	6	12	1	438.1	5	6	8	0
	456.9	458.5	5	6	12	1	438.2	5	6	8	0
	456,10	458.5	5	6	12	1	438.2	5	6	8	0
	456.11	453.5	5	6	11	0	438.2	5	6	8	0
	456.12	458.5	5	6	12	1	438.2	5	6	8	0
	456.13	453.5	5	6	11	0	438.2	5	6	8	0
	456.14	458.4	5	6	12	1	438.1	5	6	8	0
	456.16	458.9	5	6	12	1	438.5	5	6	8	0
	456.17	458.9	5	6	12	1	438.6	5	6	8	0
	456.18	453.9	5	6	11	0	438.6	5	6	8	0
	456.19	458.9	5	6	12	1	438.6	5	6	8	0
	456.20	458.9	5	6	12	1	438.6	5	6	8	0
**cc174**											
V51/1st	51.1	450.7	5	6	12*	1	655.7	5	6	10*	0
	51.2	450.7	5	6	12	1	655.7	5	6	10	0
	51.3	450.8	5	6	12	1	655.9	5	6	10	0
	51.4	450.8	5	6	12	1	655.7	5	6	10	0
	51.5	450.7	5	6	12	1	655.8	5	6	10	0
	51.6	450.8	5	6	12	1	655.9	5	6	10	0
V51/2nd	236.1	450.6	5	6	12	1	655.7	5	6	10	0
	236.2	460.9	5	6	14	0	650.5	5	6	9	1
	236.3	445.6	5	6	11	0	665.9	5	6	12	1
	236.4	455.8	5	6	13	0	655.8	5	6	10	0
	236.5	445.6	5	6	11	0	665.9	5	6	12	1
	236.6	445.7	5	6	11	0	665.9	5	6	12	1
V51/3rd	354.1	465.7	5	6	15	1	655.5	5	6	10	0
	354.2	465.8	5	6	15	1	655.4	5	6	10	0
	354.3	465.7	5	6	15	1	655.3	5	6	10	0
	254.4	465.7	5	6	15	1	655.6	5	6	10	0
	254.5	445.3	5	6	11	0	665.5	5	6	12	1

	254.6	465.7	5	6	15	1	655.5	5	6	10	0
V51/4th	424.1	445.6	5	6	11	0	665.8	5	6	12	1
	424.2	445.5	5	6	11	0	665.4	5	6	12	1
	424.3	445.6	5	6	11*	0	665.6	5	6	12*	1
	424.4	445.5	5	6	11	0	665.6	5	6	12	1
	424.5	445.5	5	6	11	0	665.7	5	6	12	1
	424.6	445.5	5	6	11	0	665.6	5	6	12	1
V52/1st	52.1	445.8	5	6	11	0	656.1	5	6	10	0
	52.2	445.8	5	6	11	0	651.0	5	6	9	1
	52.3	445.8	5	6	11	0	656.0	5	6	10	0
	52.4	445.8	5	6	11	0	656.0	5	6	10	0
	52.5	445.7	5	6	11	0	655.8	5	6	10	0
	52.6	445.7	5	6	11	0	655.9	5	6	10	0
V52/2nd	238.1	460.5	5	6	14	0	665.0	5	6	12	1
	238.2	445.8	5	6	11	0	666.1	5	6	12	1
	238.3	445.8	5	6	11	0	650.9	5	6	9	1
	238.4	445.8	5	6	11	0	651.0	5	6	9	1
	238.5	460.4	5	6	14	0	665.2	5	6	12	1
	238.6	445.8	5	6	11	0	650.9	5	6	9	1
V52/3rd	342.1	445.7	5	6	11	0	655.8	5	6	10	0
V58/1st	58.1	445.8	5	6	11	0	650.9	5	6	9	1
	58.2	445.8	5	6	11	0	650.8	5	6	9	1
	58.3	455.9	5	6	13	0	655.9	5	6	10	0
	58.4	445.8	5	6	11	0	650.8	5	6	9	1
	58.5	445.8	5	6	11	0	650.9	5	6	9	1
	58.6	456.0	5	6	13	0	656.1	5	6	10	0
V58/2nd	240.1	445.1	5	6	11	0	644.7	5	6	8	0
V58/4th	429.1	445.8	5	6	11	0	645.9	5	6	8	0
	492.2	445.8	5	6	11	0	645.9	5	6	8	0

	429.3	445.8	5	6	11	0	645.8	5	6	8	0
	429.4	445.8	5	6	11	0	645.6	5	6	8	0
	429.5	445.7	5	6	11	0	645.6	5	6	8	0
	429.6	445.8	5	6	11	0	645.7	5	6	8	0
V59/1st	59.1	450.5	5	6	12	1	655.4	5	6	10	0
	59.2	450.6	5	6	12	1	655.5	5	6	10	0
	59.3	450.6	5	6	12	1	655.5	5	6	10	0
	59.4	450.6	5	6	12	1	660.6	5	6	11	0
	59.5	450.0	5	6	12	1	655.5	5	6	10	0
	59.6	450.5	5	6	12	1	655.4	5	6	10	0
	59.11	451.1	5	6	12	1	655.8	5	6	10	0
	59.12	451.2	5	6	12	1	656.1	5	6	10	0
	59.13	451.1	5	6	12	1	656.1	5	6	10	0
	59.14	451.1	5	6	12	1	656.0	5	6	10	0
	59.15	451.1	5	6	12	1	656.0	5	6	10	0
	59.16	451.1	5	6	12	1	655.9	5	6	10	0
	59.17	451.1	5	6	12	1	655.9	5	6	10	0
	59.18	451.0	5	6	12	1	655.9	5	6	10	0
	59.20	451.4	5	6	12	1	656.5	5	6	10	0
V59/2nd	253.1	445.4	5	6	11	0	655.3	5	6	10	0
	253.2	445.4	5	6	11	0	655.4	5	6	10	0
	253.3	445.4	5	6	11	0	655.4	5	6	10	0
	253.4	445.4	5	6	11	0	655.4	5	6	10	0
	253.5	445.5	5	6	11	0	660.6	5	6	11	0
	253.6	445.4	5	6	11	0	655.4	5	6	10	0
V59/3rd	352.1	445.3	5	6	11	0	655.1	5	6	10	0
	352.2	445.3	5	6	11	0	655.1	5	6	10	0
	352.3	450.4	5	6	12	1	655.2	5	6	10	0
	352.4	445.3	5	6	11	0	655.1	5	6	10	0
	352.5	450.3	5	6	12	1	655.1	5	6	10	0

	352.6	450.4	5	6	12	1	655.1	5	6	10	0
	352.7	446.0	5	6	11	0	656.0	5	6	10	0
	352.8	451.1	5	6	12	1	656.1	5	6	10	0
	352.9	446.0	5	6	11	0	655.9	5	6	10	0
	352,10	446.0	5	6	11	0	661.2	5	6	11	0
	352.11	451.1	5	6	12	1	655.9	5	6	10	0
	352.12	446.0	5	6	11	0	656.0	5	6	10	0
	352.13	445.9	5	6	11	0	655.9	5	6	10	0
	352.14	440.8	5	6	10	0	655.8	5	6	10	0
	352.15	451.3	5	6	12	1	656.3	5	6	10	0
	352.16	456.5	5	6	13	0	656.3	5	6	10	0
	352.17	446.4	5	6	11	0	656.4	5	6	10	0
	352.18	446.4	5	6	11	0	656.5	5	6	10	0
	352.19	451.4	5	6	12	1	656.4	5	6	10	0
	352,20	451.4	5	6	12	1	656.4	5	6	10	0
V59/4th	438.1	445.3	5	6	11	0	655.3	5	6	10	0
	438.2	445.3	5	6	11	0	655.3	5	6	10	0
	438.3	450.4	5	6	12	1	655.2	5	6	10	0
	438.4	450.4	5	6	12	1	655.1	5	6	10	0
	438.5	445.2	5	6	11	0	655.1	5	6	10	0
	438.6	450.7	5	6	12	1	655.5	5	6	10	0
V138/1st	138.1	455.6	5	6	13	0	654.7	5	6	10	0
	138.2	460.6	5	6	14	0	653.7	5	6	10	0
	138.4	460.6	5	6	14	0	655.1	5	6	10	0
	138.5	445.3	5	6	11	0	654.1	5	6	10	0
	138.6	440.7	5	6	10	0	656.0	5	6	10	0
V138/2nd	288.1	445.7	5	6	11	0	666.1	5	6	12	1
	288.2	445.8	5	6	11	0	666.1	5	6	12	1
	288.3	445.7	5	6	11	0	666.0	5	6	12	1
	288.4	445.7	5	6	11	0	666.0	5	6	12	1

	288.5	445.6	5	6	11	0	665.9	5	6	12	1
	288.6	440.6	5	6	10	0	666.0	5	6	12	1
V88/1st	N88.1.1	672.6	5	6	9*	1	677.7	5	6	10	0
	N88.1.2	672.6	5	6	9	1	677.6	5	6	10	0
	N88.1.3	672.6	5	6	9	1	677.7	5	6	10	0
	N88.1.4	672.6	5	6	9	1	677.7	5	6	10	0
	N88.1.5	672.5	5	6	9	1	677.6	5	6	10	0
	N88.1.6	672.6	5	6	9	1	677.7	5	6	10*	0
	88.1.7	673.6	5	6	9	1	678.5	5	6	10	0
	88.1.8	673.6	5	6	9	1	678.6	5	6	10	0
	88.1.9	673.7	5	6	9	1	678.8	5	6	10	0
	88.1.10	673.5	5	6	9	1	678.5	5	6	10	0
	88.1	673.2	5	6	9	1	678.3	5	6	10	0
	88.2	673.2	5	6	9	1	678.4	5	6	10	0
	88.3	673.2	5	6	9	1	678.3	5	6	10	0
	88.4	673.2	5	6	9	1	678.3	5	6	10	0
	88.5	673.1	5	6	9	1	678.3	5	6	10	0
	88.6	673.1	5	6	9	1	678.2	5	6	10	0
	88.8	673.2	5	6	9	1	678.3	5	6	10	0
V88/2nd	N272.1	678.0	5	6	10	0	678.0	5	6	10	0
	N272.2	678.0	5	6	10	0	678.0	5	6	10	0
	N272.3	678.0	5	6	10	0	678.0	5	6	10	0
	N272.4	678.0	5	6	10	0	678.0	5	6	10	0
	N272.5	678.0	5	6	10	0	678.0	5	6	10	0
	N272.6	676.7	5	6	10	0	676.7	5	6	10	0
V88/3rd	N369.1	677.6	5	6	10	0	677.6	5	6	10	0
	N369.2	677.5	5	6	10	0	677.5	5	6	10	0
	N369.3	677.6	5	6	10	0	677.6	5	6	10	0
	N369.3	677.7	5	6	10	0	677.7	5	6	10	0
	N369.5	677.5	5	6	10	0	677.5	5	6	10	0

	N369.6	677.4	5	6	10	0	677.4	5	6	10	0
	369.7	678.4	5	6	10	0	678.4	5	6	10	0
	369.8	678.4	5	6	10	0	678.4	5	6	10	0
	369.9	678.6	5	6	10	0	678.6	5	6	10	0
	369,10	678.5	5	6	10	0	678.5	5	6	10	0
	369.11	678.3	5	6	10	0	678.3	5	6	10	0
	369.12	678.4	5	6	10	0	678.4	5	6	10	0
	369.14	678.3	5	6	10	0	678.3	5	6	10	0
	369.15	678.3	5	6	10	0	678.3	5	6	10	0
	369.16	678.7	5	6	10	0	678.7	5	6	10	0
	369.17	673.7	5	6	9	1	668.7	5	6	8	0
	369.18	678.7	5	6	10	0	678.7	5	6	10	0
	369.19	678.8	5	6	10	0	678.8	5	6	10	0
	369.20	678.6	5	6	10	0	678.6	5	6	10	0
V88/4th	N449.1	682.9	5	6	11*	0	687.7	5	6	12*	1
	449.2	677.8	5	6	10	0	682.9	5	6	11	0
	449.3	683.1	5	6	11	0	688.0	5	6	12	1
	449.4	683.1	5	6	11	0	688.0	5	6	12	1
	449.5	678.0	5	6	10	0	688.1	5	6	12	1
	449.6	677.9	5	6	10	0	682.9	5	6	11	0
V43/2nd	241.1	450.9	5	6	12	1	655.9	5	6	10	0
	241.2	456.0	5	6	13	0	655.9	5	6	10	0
V43/3rd	349.1	450.8	5	6	12	1	655.8	5	6	10	0
	349.2	450.9	5	6	12	1	655.9	5	6	10	0
	349.3	450.9	5	6	12	1	656.1	5	6	10	0
	349.4	450.9	5	6	12	1	656.0	5	6	10	0
	349.5	451.0	5	6	12	1	656.0	5	6	10	0
	349.6	450.9	5	6	12	1	656.1	5	6	10	0

[1]Fragments sizes were determined using either a GS500LIZ size standard. [2]Opa genes contain short C and A tracts adjacent to the 5'CTCTT repeat tract, these short tracts are invariant within a given strains as detected in all Sanger sequences of PCR products spanning the Opa repeats and in whole genome sequence data. [3]Asterisks indicate repeat numbers

confirmed by dideoxy Sanger sequencing of PCR products. [4]Expression states, 1=1, 0=0 (all genes are subject to translational phase variation).

Supplementary Data Table V. Repeat numbers and expression states for the phase-variable *opaD* and *opaJ* genes

Carrier/ Time pt	Isolate	***opaD***					***opaJ*** (5'CTCTT)				
		Size[1]	C tract[2]	A tract[2]	Repeat No.[3]	Exp. States[4]	Size[1]	C tract[2]	A tract[2]	Repeat No.[3]	Exp. States[4]
**cc23**											
V222/1st	222.1	426.7	5	6	8*	0	627.0	5	6	8*	0
	222.2	426.7	5	6	8	0	627.0	5	6	8	0
	222.3	426.1	5	6	8	0	626.4	5	6	8	0
	222.4	426.1	5	6	8	0	626.6	5	6	8	0
	222.5	426.1	5	6	8	0	625.5	5	6	8	0
	222.6	426.2	5	6	8	0	626.5	5	6	8	0
V222/2nd	309.1	426.5	5	6	8	0	627.0	5	6	8	0
	309.2	426.7	5	6	8	0	627.1	5	6	8	0
	309.3	426.6	5	6	8	0	627.1	5	6	8	0
	309.4	426.7	5	6	8	0	632.2	5	6	9	1
	309.5	426.1	5	6	8	0	626.5	5	6	8	0
	309.6	426.6	5	6	8	0	627.1	5	6	8	0
V222/3rd	391.1	426.5	5	6	8	0	639.5	3	6	8	1
	391.2	426.5	5	6	8	0	639.2	3	6	8	1
	391.3	426.8	5	6	8	0	639.5	3	6	8	1
	391.4	426.6	5	6	8	0	639.6	3	6	8	1
	391.5	426.5	5	6	8	0	639.4	3	6	8	1
	391.6	426.6	5	6	8	0	640.2	3	6	8	1
V222/4th	459.1	426.6	5	6	8	0	639.4	3	6	8	1
	459.2	426.5	5	6	8	0	639.4	3	6	8	1
	459.4	426.1	5	6	8	0	640.0	3	6	8	1
	459.4	426.1	5	6	8	0	639.6	3	6	8	1
	459.5	426.1	5	6	8*	0	639.5	3	6	8*	1
	459.6	426.2	5	6	8	0	640.3	3	6	8	1
V96/2nd	259.1	426.4	5	6	8	0	626.4	5	6	8	0
	259.2	426.3	5	6	8	0	626.4	5	6	8	0

	259.3	426.4	5	6	8	0	626.7	5	6	8	0
	259.4	426.4	5	6	8	0	626.8	5	6	8	0
	259.5	426.4	5	6	8	0	626.8	5	6	8	0
	259.6	426.3	5	6	8	0	627.1	5	6	8	0
V96/3rd	370.2	426.8	5	6	8	0	647.4	5	6	12	1
	370.3	421.9	5	6	7	0	627.5	5	6	8	0
	370.4	426.8	5	6	8	0	647.4	5	6	12	1
	370.5	426.8	5	6	8	0	622.4	5	6	7	0
	370.6	421.7	5	6	7	0	647.5	5	6	12	1
V96/4th	445.1	426.2	5	6	8	0	626.8	5	6	8	0
	445.2	426.2	5	6	8	0	626.3	5	6	8	0
	445.3	426.3	5	6	8	0	626.9	5	6	8	0
	445.4	426.3	5	6	8	0	626.8	5	6	8	0
	445.5	426.2	5	6	8	0	627.1	5	6	8	0
	445.6	426.3	5	6	8	0	626.7	5	6	8	0
V69/2nd	258.1	426.3	5	6	8	0	626.6	5	6	8	0
	258.2	426.4	5	6	8	0	626.9	5	6	8	0
	258.3	426.3	5	6	8	0	626.7	5	6	8	0
	258.4	426.8	5	6	8	0	627.3	5	6	8	0
	258.5	426.7	5	6	8	0	627.2	5	6	8	0
	258.6	426.2	5	6	8	0	626.6	5	6	8	0
V69/4th	431.1	431.6	5	6	9	1	626.8	5	6	8	0
	431.2	426.5	5	6	8	0	626.9	5	6	8	0
	431.3	426.6	5	6	8	0	626.8	5	6	8	0
	431.4	426.8	5	6	8	0	627.1	5	6	8	0
	431.5	426.8	5	6	8	0	627.0	5	6	8	0
	431.6	426.8	5	6	8	0	627.1	5	6	8	0
V93/2nd	264.1	421.7	5	6	7	0	622.0	5	6	7	0
	264.2	421.7	5	6	7	0	622.1	5	6	7	0

	264.3	421.3	5	6	7	0	621.7	5	6	7	0
	264.4	421.6	5	6	7	0	622.0	5	6	7	0
	264.5	421.6	5	6	7	0	621.7	5	6	7	0
	264.6	421.1	5	6	7	0	621.5	5	6	7	0
V93/3rd	359.1	421.9	5	6	7	0	622.2	5	6	7	0
	359.2	421.8	5	6	7	0	622.3	5	6	7	0
	359.3	421.5	5	6	7	0	621.9	5	6	7	0
	359.4	421.7	5	6	7	0	622.3	5	6	7	0
	359.5	421.8	5	6	7	0	622.2	5	6	7	0
	359.6	421.4	5	6	7	0	621.7	5	6	7	0
**cc167**											
V117/1st	117.1	420.6	5	6	4	0	616.5	5	6	4	0
	117.2	420.5	5	6	4*	0	616.7	5	6	4*	0
V117/2nd	284.1	421.0	5	6	4	0	617.0	5	6	4	0
	284.2	421.1	5	6	4	0	612.0	5	6	3	1
	284.3	421.1	5	6	4	0	617.1	5	6	4	0
	284.4	421.0	5	6	4	0	611.8	5	6	3	1
	284.5	421.0	5	6	4	0	616.9	5	6	4	0
	284.6	421.0	5	6	4	0	611.8	5	6	3	1
V117/3rd	332.1	420.6	5	6	4*	0	611.5	5	6	3	1
	332.2	420.7	5	6	4	0	611.7	5	6	3	1
	332.3	420.7	5	6	4	0	611.7	5	6	3	1
	332.4	420.7	5	6	4	0	611.6	5	6	3	1
	332.6	420.6	5	6	4	0	611.6	5	6	3	1
V117/4th	417.1	420.4	5	6	4	0	616.4	5	6	4	0
	417.2	421.1	5	6	4	0	617.1	5	6	4	0
	417.3	421.1	5	6	4	0	617.2	5	6	4	0
	417.4	420.4	5	6	4	0	616.2	5	6	4	0
	417.5	421.2	5	6	4	0	617.0	5	6	4	0
	417.6	421.1	5	6	4	0	617.1	5	6	4	0

V64/1st	64.1	420.6	5	6	4	0	657.1	5	6	12*	1
	64.2	420.6	5	6	4	0	657.1	5	6	12	1
	64.3	420.7	5	6	4	0	657.0	5	6	12	1
	64.4	420.5	5	6	4	0	657.2	5	6	12	1
	64.5	420.5	5	6	4	0	656.9	5	6	12	1
	64.6	420.6	5	6	4	0	657.0	5	6	12	1
V64/2nd	257.1	420.5	5	6	4	0	651.8	5	6	11	0
	257.2	421.2	5	6	4	0	652.3	5	6	11	0
	257.3	421.2	5	6	4	0	652.2	5	6	11	0
	257.4	421.1	5	6	4	0	652.4	5	6	11	0
	257.5	421.1	5	6	4	0	652.2	5	6	11	0
	257.6	421.2	5	6	4	0	652.2	5	6	11	0
V64/3rd	348.1	420.5	5	6	4	0	641.7	5	6	9	1
	348.2	420.6	5	6	4	0	636.8	5	6	8	0
	348.3	420.5	5	6	4	0	636.8	5	6	8	0
	348.4	420.6	5	6	4	0	636.7	5	6	8	0
	348.5	420.5	5	6	4	0	641.6	5	6	9	1
	348.6	420.6	5	6	4	0	641.4	5	6	9	1
V124/1st	124.1	421.0	5	6	4	0	617.0	5	6	4	0
	124.2	421.1	5	6	4	0	617.0	5	6	4*	0
	124.3	420.5	5	6	4	0	616.3	5	6	4	0
	124.4	420.5	5	6	4	0	616.3	5	6	4	0
	124.5	420.5	5	6	4	0	616.3	5	6	4	0
V124/2nd	290.1	420.5	5	6	4	0	616.4	5	6	4	0
	290.3	421.0	5	6	4	0	616.9	5	6	4	0
	290.4	420.9	5	6	4	0	616.8	5	6	4	0
	290.5	420.5	5	6	4	0	616.5	5	6	4	0
	290.6	420.4	5	6	4	0	616.4	5	6	4	0
V124/3rd	336.1	420.5	5	6	4	0	616.6	5	6	4	0

	336.2	420.7	5	6	4	0	616.7	5	6	4	0
	336.3	421.0	5	6	4	0	617.0	5	6	4	0
	336.4	420.8	5	6	4	0	616.8	5	6	4	0
	336.5	420.6	5	6	4	0	616.7	5	6	4	0
	336.6	420.5	5	6	4	0	616.6	5	6	4	0
**cc60**											
V113/1st	113.1	464.3	4	6	14*	1	413.4	4	6	4*	0
	113.2	464.4	4	6	14	1	413.5	4	6	4	0
	113.3	464.5	4	6	14	1	413.5	4	6	4	0
	113.4	464.3	4	6	14	1	413.4	4	6	4	0
	113.5	464.4	4	6	14	1	413.4	4	6	4	0
	113.6	464.3	4	6	14	1	413.5	4	6	4	0
V113/2nd	281.1	464.7	4	6	14	1	413.8	4	6	4	0
	281.2	464.7	4	6	14	1	413.9	4	6	4	0
	281.3	464.8	4	6	14	1	413.9	4	6	4	0
	281.4	469.8	4	6	15	0	413.8	4	6	4	0
	281.5	434.3	4	6	8	1	413.8	4	6	4	0
	281.6	434.3	4	6	8	1	413.9	4	6	4	0
V114/1st	114.1	464.4	4	6	14	1	413.5	4	6	4	0
	114.2	469.5	4	6	15	0	413.5	4	6	4	0
	114.3	464.5	4	6	14	1	413.6	4	6	4	0
	114.4	459.4	4	6	13	0	413.5	4	6	4	0
	114.5	464.4	4	6	14	1	413.6	4	6	4	0
	114.6	464.5	4	6	14	1	413.5	4	6	4	0
	114.7	464.4	4	6	14	1	413.7	4	6	4	0
	114.8	464.5	4	6	14	1	413.5	4	6	4	0
	114.9	464.5	4	6	14	1	413.5	4	6	4	0
	114,10	464.5	4	6	14	1	413.6	4	6	4	0
	114.11	464.5	4	6	14	1	413.6	4	6	4	0

	114.12	464.6	4	6	14	1	413.6	4	6	4	0
	114.13	474.6	4	6	16	0	413.5	4	6	4	0
	114.14	464.4	4	6	14	1	413.5	4	6	4	0
	114.16	464.8	4	6	14	1	414.4	4	6	4	0
	114.18	464.9	4	6	14	1	414.3	4	6	4	0
V114/2nd	283.1	459.3	4	6	13	0	413.4	4	6	4	0
	283.2	469.9	4	6	15	0	414.1	4	6	4	0
	283.3	459.7	4	6	13	0	414.0	4	6	4	0
	283.4	459.3	4	6	13	0	413.1	4	6	4	0
	283.5	459.6	4	6	13	0	413.9	4	6	4	0
	283.6	459.7	4	6	13	0	414.0	4	6	4	0
V114/3rd	330.1	469.3	4	6	15	0	413.5	4	6	4	0
	330.2	469.3	4	6	15	0	413.3	4	6	4	0
	330.3	469.7	4	6	15	0	413.8	4	6	4	0
	330.4	469.6	4	6	15	0	413.6	4	6	4	0
	330.5	469.4	4	6	15	0	413.5	4	6	4	0
	330.6	469.4	4	6	15	0	413.3	4	6	4	0
	330.7	469.6	4	6	15	0	413.5	4	6	4	0
	330.8	469.7	4	6	15	0	413.6	4	6	4	0
	330.9	469.6	4	6	15	0	413.5	4	6	4	0
	330,10	469.6	4	6	15	0	413.7	4	6	4	0
	330.11	469.5	4	6	15	0	413.6	4	6	4	0
	330.12	469.5	4	6	15	0	413.6	4	6	4	0
	330.15	469.9	4	6	15	0	414.1	4	6	4	0
	330.16	470.0	4	6	15	0	414.2	4	6	4	0
	330.17	470.0	4	6	15	0	414.2	4	6	4	0
	330.18	469.9	4	6	15	0	414.2	4	6	4	0
	330.19	470.0	4	6	15	0	414.1	4	6	4	0
	330.20	470.0	4	6	15	0	414.1	4	6	4	0
V115/1st	115.1	464.4	4	6	14	1	413.5	4	6	4	0

	115.2	433.9	4	6	8	1	413.5	4	6	4	0
	115.3	464.5	4	6	14	1	413.5	4	6	4	0
	115.4	464.4	4	6	14	1	413.6	4	6	4	0
	115.5	464.3	4	6	14	1	413.6	4	6	4	0
	115.6	464.3	4	6	14	1	413.5	4	6	4	0
V115/2nd	282.1	464.4	4	6	14	1	413.4	4	6	4	0
	282.2	469.9	4	6	15	0	414.1	4	6	4	0
	282.3	464.8	4	6	14	1	414.0	4	6	4	0
	282.4	464.8	4	6	14	1	414.1	4	6	4	0
	282.5	464.8	4	6	14	1	414.0	4	6	4	0
	282.6	464.9	4	6	14	1	414.0	4	6	4	0
V134/1st	134.1	464.3	4	6	14	1	413.4	4	6	4	0
	134.2	459.3	4	6	13	0	413.5	4	6	4	0
	134.3	459.2	4	6	13	0	413.4	4	6	4	0
	134.4	459.3	4	6	13	0	413.5	4	6	4	0
	134.6	459.2	4	6	13	0	413.5	4	6	4	0
	134.7	459.4	4	6	13	0	413.6	4	6	4	0
	134.8	459.4	4	6	13	0	413.5	4	6	4	0
	134.9	459.4	4	6	13	0	413.6	4	6	4	0
	134,10	459.4	4	6	13	0	413.6	4	6	4	0
	134.11	459.4	4	6	13	0	413.5	4	6	4	0
	134.12	459.3	4	6	13	0	413.5	4	6	4	0
	134.13	459.3	4	6	13	0	413.3	4	6	4	0
	134.14	459.3	4	6	13	0	413.5	4	6	4	0
	134.16	459.7	4	6	13	0	414.2	4	6	4	0
	134.17	459.8	4	6	13	0	414.2	4	6	4	0
	134.18	464.9	4	6	14	1	414.3	4	6	4	0
V134/2nd	295.1	464.3	4	6	14	1	413.5	4	6	4	0
	295.2	464.8	4	6	14	1	414.0	4	6	4	0
	295.3	459.3	4	6	13	0	413.5	4	6	4	0

	295.4	464.4	4	6	14	1	413.5	4	6	4	0
	295.5	459.2	4	6	13	0	413.6	4	6	4	0
	295.6	464.7	4	6	14	1	413.8	4	6	4	0
V134/3rd	333.1	459.3	4	6	13	0	413.4	4	6	4	0
	333.2	459.3	4	6	13	0	413.3	4	6	4	0
	333.3	459.7	4	6	13	0	413.8	4	6	4	0
	333.4	459.5	4	6	13	0	413.7	4	6	4	0
	333.5	459.3	4	6	13	0	413.4	4	6	4	0
	333.6	459.2	4	6	13	0	413.4	4	6	4	0
	333.7	459.4	4	6	13	0	413.7	4	6	4	0
	333.8	459.4	4	6	13	0	413.6	4	6	4	0
	333.9	459.5	4	6	13	0	413.7	4	6	4	0
	333.10	459.6	4	6	13	0	413.7	4	6	4	0
	333.11	459.6	4	6	13	0	413.6	4	6	4	0
	333.12	459.4	4	6	13	0	413.7	4	6	4	0
	333.13	464.5	4	6	14	0	413.6	4	6	4	0
	333.14	459.4	4	6	13	0	413.5	4	6	4	0
	333.15	459.7	4	6	13	0	414.1	4	6	4	0
	333.16	459.8	4	6	13	0	414.2	4	6	4	0
	333.17	459.9	4	6	13	0	414.2	4	6	4	0
	333.18	459.9	4	6	13	0	414.2	4	6	4	0
	333.19	459.8	4	6	13	0	414.1	4	6	4	0
	333.20	459.7	4	6	13	0	414.1	4	6	4	0
V185/1st	185.1	438.8	4	6	9	0	428.8	4	6	7	0
	185.2	438.9	4	6	9	0	429.0	4	6	7	0
	185.3	438.9	4	6	9*	0	428.9	4	6	7	0
	185.4	439.0	4	6	9	0	429.0	4	6	7	0
	185.5	439.0	4	6	9	0	428.9	4	6	7	0
	185.6	438.9	4	6	9	0	428.9	4	6	7	0
	185.8	438.9	4	6	9	0	428.9	4	6	7	0

	185.9	439.2	4	6	9	0	429.1	4	6	7	0
	185.10	439.1	4	6	9	0	428.9	4	6	7	0
	185.11	439.0	4	6	9	0	428.8	4	6	7	0
	185.12	439.0	4	6	9	0	429.0	4	6	7	0
	185.13	438.9	4	6	9	0	428.9	4	6	7	0
	185.14	438.9	4	6	9	0	429.0	4	6	7	0
	185.15	438.8	4	6	9	0	428.6	4	6	7	0
	185.16	439.3	4	6	9	0	429.3	4	6	7	0
	185.17	439.3	4	6	9	0	429.3	4	6	7	0
	185.18	439.4	4	6	9	0	429.3	4	6	7	0
	185.19	439.4	4	6	9	0	429.3	4	6	7	0
	185.20	439.4	4	6	9	0	429.4	4	6	7	0
V185/2nd	306.1	443.9	4	6	10	0	428.9	4	6	7	0
	306.2	443.9	4	6	10	0	428.8	4	6	7	0
	306.3	449.1	4	6	11	1	428.8	4	6	7	0
	306.4	444.0	4	6	10	0	428.9	4	6	7	0
	306.5	443.9	4	6	10	0	428.8	4	6	7	0
	306.6	449.0	4	6	11	1	428.8	4	6	7	0
V185/4th	456.1	448.9	4	6	11	1	428.7	4	6	7	0
	456.2	448.9	4	6	11	1	428.7	4	6	7	0
	456.3	459.2	4	6	13	0	429.0	4	6	7	0
	456.4	459.2	4	6	13	0	428.7	4	6	7	0
	456.5	459.2	4	6	13	0	428.8	4	6	7	0
	456.6	449.0	4	6	11	1	428.7	4	6	7	0
	456.7	449.2	4	6	11	1	429.5	4	6	7	0
	456.8	459.2	4	6	13	0	428.7	4	6	7	0
	456.9	459.3	4	6	13	0	428.6	4	6	7	0
	456.10	459.2	4	6	13	0	428.8	4	6	7	0
	456.11	449.1	4	6	11	1	428.9	4	6	7	0
	456.12	459.2	4	6	13	0	428.9	4	6	7	0
	456.13	449.1	4	6	11	1	428.7	4	6	7	0

	456.14	459.2	4	6	13	0	428.9	4	6	7	0
	456.16	459.5	4	6	13	0	428.9	4	6	7	0
	456.17	459.6	4	6	13	0	429.0	4	6	7	0
	456.18	449.5	4	6	11	1	429.1	4	6	7	0
	456.19	459.6	4	6	13	0	429.3	4	6	7	0
	456.20	459.7	4	6	13	0	429.3	4	6	7	0
**cc174**											
V51/1ST	51.1	640.8	5	6	8*	0	433.7	5	5	7*	0
	51.2	641.1	5	6	8	0	438.7	5	5	8	1
	51.3	646.0	5	6	9	1	438.9	5	5	8	1
	51.4	646.1	5	6	9	1	438.7	5	5	8	1
	51.5	640.9	5	6	8	0	438.7	5	5	8	1
	51.6	646.0	5	6	9	1	438.6	5	5	8	1
V51/2nd	236.1	641.0	5	6	8	0	438.7	5	5	8	1
	236.2	641.0	5	6	8	0	443.8	5	5	9	0
	236.3	641.1	5	6	8	0	433.8	5	5	7	0
	236.4	641.1	5	6	8	0	438.7	5	5	8	1
	236.5	641.1	5	6	8	0	433.7	5	5	7	0
	236.6	641.1	5	6	8	0	433.7	5	5	7	0
V51/3rd	354.1	640.8	5	6	8	0	443.6	5	5	9	0
	354.2	641.0	5	6	8	0	443.6	5	5	9	0
	354.3	640.9	5	6	8	0	443.6	5	5	9	0
	254.4	641.0	5	6	8	0	443.7	5	5	9	0
	254.5	641.0	5	6	8	0	433.6	5	5	7	0
	254.6	640.9	5	6	8	0	443.6	5	5	9	0
V51/4th	424.1	640.9	5	6	8	0	433.6	5	5	7	0
	424.2	640.9	5	6	8	0	433.5	5	5	7	0
	424.3	640.9	5	6	8*	0	433.6	5	5	7*	0
	424.4	640.9	5	6	8	0	433.4	5	5	7	0
	424.5	640.9	5	6	8	0	433.6	5	5	7	0

	424.6	640.8	5	6	8	0	438.5	5	5	8	1
V52/1st	52.1	641.3	5	6	8	0	439.0	5	5	8	1
	52.2	641.1	5	6	8	0	438.9	5	5	8	1
	52.3	641.2	5	6	8	0	439.0	5	5	8	1
	52.4	641.2	5	6	8	0	439.0	5	5	8	1
	52.5	641.1	5	6	8	0	438.8	5	5	8	1
	52.6	641.1	5	6	8	0	438.9	5	5	8	1
V52/2nd	238.1	640.2	5	6	8	0	432.9	5	5	7	0
	238.2	641.2	5	6	8	0	433.9	5	5	7	0
	238.3	641.2	5	6	8	0	449.2	5	5	10	0
	238.4	641.3	5	6	8	0	434.0	5	5	7	0
	238.5	640.0	5	6	8	0	443.2	5	5	9	0
	238.6	641.3	5	6	8	0	449.1	5	5	10	0
V52/3rd	342.1	641.1	5	6	8	0	438.8	5	5	8	1
V58/1st	58.1	646.3	5	6	9	1	444.0	5	5	9	0
	58.2	646.2	5	6	9	1	444.0	5	5	9	0
	58.3	641.2	5	6	8	0	438.8	5	5	8	1
	58.4	646.2	5	6	9	1	443.8	5	5	9	0
	58.5	645.6	5	6	9	1	443.0	5	5	9	0
	58.6	641.3	5	6	8	0	439.0	5	5	8	1
V58/2nd	240.1	640.3	5	6	8	0	437.9	5	5	8	1
V58/3rd	429.1	641.2	5	6	8	0	438.9	5	5	8	1
	492.2	641.1	5	6	8	0	438.9	5	5	8	1
	429.3	641.2	5	6	8	0	438.9	5	5	8	1
	429.4	641.1	5	6	8	0	438.8	5	5	8	1
	429.5	641.1	5	6	8	0	438.8	5	5	8	1
	429.6	641.1	5	6	8	0	438.8	5	5	8	1
V59/1st	59.1	640.7	5	6	8	0	433.5	5	5	7	0

	59.2	640.7	5	6	8	0	438.5	5	5	8	1
	59.3	640.8	5	6	8	0	443.5	5	5	9	0
	59.4	640.7	5	6	8	0	438.4	5	5	8	1
	59.5	640.5	5	6	8	0	433.2	5	5	7	0
	59.6	640.6	5	6	8	0	433.3	5	5	7	0
	59.11	640.6	5	6	8	0	433.2	5	5	7	0
	59.12	640.8	5	6	8	0	433.4	5	5	7	0
	59.13	640.8	5	6	8	0	433.2	5	5	7	0
	59.14	640.8	5	6	8	0	433.3	5	5	7	0
	59.15	640.7	5	6	8	0	433.3	5	5	7	0
	59.16	645.7	5	6	9	0	438.2	5	5	8	1
	59.17	640.6	5	6	8	0	433.1	5	5	7	0
	59.18	640.5	5	6	8	0	433.1	5	5	7	0
	59.20	641.2	5	6	8	0	438.7	5	5	8	1
V59/2nd	253.1	640.7	5	6	8	0	433.4	5	5	7	0
	253.2	640.7	5	6	8	0	433.4	5	5	7	0
	253.3	635.7	5	6	7	0	433.5	5	5	7	0
	253.4	640.8	5	6	8	0	433.4	5	5	7	0
	253.5	640.8	5	6	8	0	433.4	5	5	7	0
	253.6	640.7	5	6	8	0	433.3	5	5	7	0
V59/3rd	352.1	640.6	5	6	8	0	438.2	5	5	8	1
	352.2	640.6	5	6	8	0	438.3	5	5	8	1
	352.3	640.6	5	6	8	0	433.2	5	5	7	0
	352.4	640.6	5	6	8	0	438.2	5	5	8	1
	352.5	640.7	5	6	8	0	433.3	5	5	7	0
	352.6	640.6	5	6	8	0	433.1	5	5	7	0
	352.7	640.5	5	6	8	0	438.2	5	5	8	1
	352.8	640.6	5	6	8	0	433.3	5	5	7	0
	352.9	640.7	5	6	8	0	433.4	5	5	7	0
	352,10	640.7	5	6	8	0	438.2	5	5	8	1
	352.11	640.6	5	6	8	0	433.2	5	5	7	0

	352.12	640.5	5	6	8	0	438.2	5	5	8	1
	352.13	640.6	5	6	8	0	438.1	5	5	8	1
	352.14	640.6	5	6	8	0	438.0	5	5	8	1
	352.15	641.0	5	6	8	0	433.6	5	5	7	0
	352.16	641.1	5	6	8	0	433.6	5	5	7	0
	352.17	641.1	5	6	8	0	433.6	5	5	7	0
	352.18	641.2	5	6	8	0	438.7	5	5	8	1
	352.19	641.2	5	6	8	0	433.6	5	5	7	0
	352,20	641.1	5	6	8	0	433.6	5	5	7	0
V59/4th	438.1	645.8	5	6	9	1	438.4	5	5	8	1
	438.2	645.6	5	6	9	1	438.2	5	5	8	1
	438.3	645.6	5	6	9	1	438.3	5	5	8	1
	438.4	645.7	5	6	9	1	433.3	5	5	7	0
	438.5	645.6	5	6	9	1	438.3	5	5	8	1
	438.6	645.5	5	6	9	1	437.9	5	5	8	1
V138/1st	138.1	640.3	5	6	8	0	432.8	5	5	7	0
	138.2	640.5	5	6	8	0	433.0	5	5	7	0
	138.4	640.3	5	6	8	0	432.9	5	5	7	0
	138.5	640.4	5	6	8	0	437.8	5	5	8	1
	138.6	641.3	5	6	8	0	433.8	5	5	7	0
V138/2nd	288.1	641.1	5	6	8	0	433.8	5	5	7	0
	288.2	641.1	5	6	8	0	433.7	5	5	7	0
	288.3	641.0	5	6	8	0	433.7	5	5	7	0
	288.4	645.3	5	6	9	1	433.7	5	5	7	0
	288.5	641.0	5	6	8	0	433.6	5	5	7	0
	288.6	640.9	5	6	8	0	433.6	5	5	7	0
V88/1st	N88.1.1	671.1	5	6	14	0	441.3	5	5	7	0
	N88.1.	676.4	5	6	15	1	441.4	5	5	7	0

	2										
	N88.1.3	671.2	5	6	14	0	441.5	5	5	7	0
	N88.1.4	671.1	5	6	14	0	441.4	5	5	7	0
	N88.1.5	681.2	5	6	16	0	441.4	5	5	7	0
	N88.1.6	671.1	5	6	14	0	441.3	5	5	7	0
	88.1.7	671.6	5	6	14	0	441.4	5	5	7	0
	88.1.8	671.7	5	6	14	0	441.5	5	5	7	0
	88.1.9	671.8	5	6	14	0	441.4	5	5	7	0
	88.1.10	676.6	5	6	15	1	441.3	5	5	7	0
	88.1	671.2	5	6	14	0	441.0	5	5	7	0
	88.2	671.2	5	6	14	0	441.0	5	5	7	0
	88.3	671.3	5	6	14	0	441.0	5	5	7	0
	88.4	671.3	5	6	14	0	441.0	5	5	7	0
	88.5	671.1	5	6	14	0	440.9	5	5	7	0
	88.6	671.1	5	6	14	0	441.0	5	5	7	0
	88.8	671.1	5	6	14	0	440.7	5	5	7	0
V88/2nd	N272.1	671.5	5	6	14*	0	441.0	5	5	7*	0
	N272.2	671.6	5	6	14	0	441.0	5	5	7	0
	N272.3	671.6	5	6	14	0	441.0	5	5	7	0
	N272.4	671.5	5	6	14	0	440.9	5	5	7	0
	N272.5	671.5	5	6	14	0	440.9	5	5	7	0
	N272.6	671.5	5	6	14	0	441.0	5	5	7	0
V88/3rd	N369.1	665.9	5	6	13	0	446.3	5	5	8	1
	N369.2	665.9	5	6	13	0	446.4	5	5	8	1
	N369.3	661.1	5	6	12	1	441.4	5	5	7	0
	N369.3	660.8	5	6	12	1	441.3	5	5	7	0
	N369.5	665.9	5	6	13	0	446.4	5	5	8	1
	N369.6	665.9	5	6	13	0	446.3	5	5	8	1

	369.7	661.2	5	6	12	1	441.1	5	5	7	0
	369.8	666.2	5	6	13	0	446.2	5	5	8	1
	369.9	661.2	5	6	12	0	441.2	5	5	7	0
	369,10	666.4	5	6	13	0	446.2	5	5	8	1
	369.11	666.2	5	6	13	0	446.1	5	5	8	1
	369.12	671.3	5	6	14	0	446.1	5	5	8	1
	369.14	666.2	5	6	13	0	446.1	5	5	8	1
	369.15	661.1	5	6	12	1	440.9	5	5	7	0
	369.16	666.5	5	6	13	0	446.5	5	5	8	1
	369.17	681.9	5	6	16	0	446.6	5	5	8	1
	369.18	661.6	5	6	12	1	441.5	5	5	7	0
	369.19	661.5	5	6	12	1	441.6	5	5	7	0
	369,20	661.4	5	6	12	1	441.5	5	5	7	0
V88/4th	N449.1	681.2	5	6	16	0	440.4	5	5	7	0
	449.2	676.4	5	6	15	1	440.4	5	5	7	0
	449.3	676.6	5	6	15	1	440.5	5	5	7	0
	449.4	676.4	5	6	15	1	440.5	5	5	7	0
	449.5	671.3	5	6	14*	0	440.7	5	5	7*	0
	449.6	676.5	5	6	15	1	440.6	5	5	7	0
V43/2nd	241.1	651.3	5	6	10	0	438.9	5	5	8	1
	241.2	651.3	5	6	10	0	438.9	5	5	8	1
V43/3rd	349.1	646.1	5	6	9	1	443.9	5	5	9	0
	349.2	646.1	5	6	9	1	444.0	5	5	9	0
	349.3	646.4	5	6	9	1	444.0	5	5	9	0
	349.4	646.4	5	6	9	1	444.1	5	5	9	0
	349.5	646.3	5	6	9	1	444.0	5	5	9	0
	349.6	646.3	5	6	9	1	444.0	5	5	9	0

[1]Fragments sizes were determined using a GS500LIZ size standard. [2]Opa genes contain short C and A tracts adjacent to the 5'CTCTT repeat tract, these short tracts are invariant within a given strains as detected in all Sanger sequences of PCR products spanning the Opa repeats and in whole genome sequence data. [3]Asterisks indicate repeat numbers

confirmed by dideoxy Sanger sequencing of PCR products. [4]Expression states, 1=1, 0=0 (all genes are subject to translational phase variation).
